# Evolutionary and Expression Analysis Provides Evidence for the Plant Glutamate-like Receptors Family is Involved in Woody Growth-related Function

**DOI:** 10.1038/srep32013

**Published:** 2016-08-24

**Authors:** Jianqing Chen, Yinghui Jing, Xinyue Zhang, Leiting Li, Peng Wang, Shaoling Zhang, Hongsheng Zhou, Juyou Wu

**Affiliations:** 1 Center of Pear Engineering Technology Research, State Key Laboratory of Crop Genetics and Germplasm Enhancement, College of Horticulture, Nanjing Agricultural University, Nanjing 210095, China; 2 Institute of Agricultural Products Processing, Jiangsu Academy of Agricultural Sciences, Nanjing 210014, China

## Abstract

Glutamate-like receptors (GLRs) is a highly conserved family of ligand-gated ion channels, which have been associated with various physiological and developmental processes. Here, we investigated the evolutionary pattern of *GLRs* in plants. We observed that tandem duplications occupied the largest proportion of the plant *GLR* gene family expansion. Based on a phylogenetic tree, we suggested a new subfamily, *GLR4*, which is widespread in angiosperm but absence on Brassicales. Meanwhile, because *GLR1* and *GLR2* subfamilies were potential sister clades, we combined them into the *GLR1&2* subfamily. A comparative analysis of plant *GLR* subfamilies revealed that selective forces shaped the *GLR1&2* repertoires in the stems of eudicotyledons with distinct functional preferences. Moreover, *GLR1&2* formed a species-specific highwoody-expanded subfamily, with preferential expression in the cambial-enriched and shoot apical meristem fractions of the highwood species. Together, these findings lay the foundation for evolutionary analysis of plant *GLRs* over the entire plant timescale and identified unique targets for manipulating the woody-growth behaviours of plant GLRs.

Amino acids are formed from incorporation of inorganic nitrogen, and they serve as nitrogen signaling molecules in animals and plants. Plant glutamate-like receptors (GLRs), counterparts of mammalian ionotropic glutamate receptors (iGluRs), are assumed to be the prime amino acid sensors in plants. A parsimony phylogenetic analysis showed that plant GLRs preceded the divergence of animal and protokaryotic iGluR classes, and in *Arabidopsis* can be divided into three clades, GLR1, GLR2 and GLR3^1^. Moreover, the GLR1 and GLR2 subfamilies are thought to have a sister relationship in the parsimony tree of *Arabidopsis* GLRs, which was supported by their overlapping expression patterns^1^.

Despite the classification, plant GLRs as predicted ligand-gated ion channels share the basic structures of the channel-forming domains and ligand-binding sites^2^. The channel-forming domain consists of three complete trans-membrane domains (M1, M3 and M4) plus a partial trans-membrane domain that forms a pore-loop structure (M2-Pore)^3^. The predicted pore structure has functional cation channels and transplantation of AtGLR1.1 and AtGLR1.4 pore domains into rat GluR1 and GluR6 chimeras, respectively, led to conductance of cations^4^. A ligand-binding domain consists of two lobes, GlnH1 and GlnH2, which are related to channel-gating through agonist-induced conformational changes^5^. Moreover, plant GLRs have an additional modulatory region, the amino-terminal domain (ATD), which in iGluRs can regulate channel activities by binding a wide range of ions and molecules^6^. Furthermore, the resolved crystal structure of animal iGluRs revealed that ATDs regulate channel activities through interactions between the subunits to determine the channel composition^7^.

Analogous to mammalian iGluRs, plant GLRs conduct many cations into the cell in the presence of extracellular amino acids, such as AtGLR3.4, which are highly selective to calcium ions (Ca^2+^)^8^. As Ca^2+^ is a main signal messenger in plants, GLRs could play fundamental roles in mediation of diverse cellular responses in plant cells. Indeed, *AtGLR3.1* is preferentially expressed in stomatal guard cells, and *AtGLR3.1* over-expression elicited in impaired external Ca^2+^-induced stomatal closure, suggests that *AtGLR3.1* is correlated with the cytosolic Ca^2+^ concentration that regulates stomatal movements^9^. *AtGLR3.5*-mediated Ca^2+^ signaling promotes seed germination by counteracting the inhibitory effects of abscisic acid through the repression of ABI4^10^. Applying exogenous glutathione to *Arabidopsis* leaves triggered a transient rise in cytosolic Ca^2+^ concentration, which is highly sensitive to GLR antagonists, and was also impaired in loss-of-function *atglr3.3* mutants^11^. These mutants showed enhanced susceptibility to bacterial pathogens, lower expression of pathogen-induced defence marker genes and similar phenotype to the glutathione biosynthesis defective mutant *pad2*^11^. Under D-Ser activation, *AtGLR1.2* mediated Ca^2+^ influx into pollen tube cells as they extended in the pistil. The Ca^2+^ signature is largely altered in *glr1.2* pollen tubes^12^.

Highwood species’ properties differ from those of other plant species and are mainly determined by the relative abundance of wood. Wood forms during secondary growth through activity of the vascular cambium, a layer of meristematic cells on the outer periphery of the stem. Cambial initials produce xylem mother cells^13^,^14^, differentiate into mature xylem cells, including vessels and fibres. Xylem differentiation and cambial activity are spatially and temporally regulated at the transcriptional level and involve a complex hierarchical signal transduction network^15^. Ca^2+^ has positive effects on wood formation in coniferous trees^16^. Moreover, increases in Ca^2+^ in cambium, xylem and phloem occur with increased Ca^2+^ supply in nutrient solution^17^. Likewise, stable isotope labelling showed 60–70% of Ca^2+^ content in vascular tissue, suggesting that long-distance transport Ca^2+^ into woody tissue^18^. Furthermore, under high titre Ca^2+^ stress, poplar wood formation can be strongly inhibited shown in decreased vessel sizes and wood increments, decreased specific performances in reduced layers of cambial and xylem differentiation zones in the radial direction, and decreased lengths of young libriform fibres^17^. Secondary ion mass spectrometry showed a strikingly temporary increase in Ca^2+^ concentration in the cambium during the reactivation period^19^, suggesting that Ca^2+^ acts as signaling messenger, triggering cambium activation. Indeed, phenotypic investigation of poplar stem tissues under increasing Ca^2+^ concentrations in cambium showed a much wider cambial zone with larger vacuoles, which is typical of active vascular cambium contrasting to cells filled with dense cytoplasm and decreasing cambial width with a reduced Ca^2+^ supply^17^. Additionally, Ca^2+^ reportedly increased in the apical meristem during bud flush and the beginning of cell division^20^. These results further show that cambium activation relies on Ca^2+^ signaling. Thus, we suggest that potential Ca^2+^-transporters could participate in Ca^2+^ long-distance transport and responses to wood formation.

At least two AtGLR (GLR3.4 and GLR3.2) proteins are enriched primarily in the phloem and play fundamental roles in Ca^2+^ signal transduction^21^. In parallel, using non-invasive electrodes, Mousavi *et al*. mapped surface potential changes in *Arabidopsis* after wounding leaves and found membrane depolarization domains in leaves distal to the wounds^22^. Furthermore, mutations in several *GLR3* genes (*3.2*, *3.3* and *3.6*) alleviated wound-induced surface potential changes and correlated with jasmonate-response gene expression, which was more attenuated in a *glr3.3 glr3.6* double mutant^22^. This suggests that electrical impulses act as intracellular signals and that plant *GLRs* are potent regulators in this long-distance communication in wound-induced defence responses. These results support that long-distance signaling exists in plants and indicates that plant GLRs analogous to those important for synaptic activity in animals have roles in organ-organ Ca^2+^ signal transmission.

Plant GLRs, as extracellular amino acid sensors, play fundamental roles in regulating various biological processes. However, knowledge of this gene family is confined to *Arabidopsis thaliana*. Thus, further investigations of *GLRs* in other plant species are necessary to determine more gene family profiles. Rosaceae, as a core plant lineage, and composing many highwood species, have had their genomes extensively sequenced^23^,^24^,^25^,^26^,^27^,^28^. This provides an opportunity to further analyse the *GLR* family in highwood species. We collected genome data and used Rosaceae as a model for insight into plant GLR evolution. We combined comparative genomics, molecular evolutionary studies and expression analyses to examine the evolution of plant GLRs and investigate characteristics of highwood GLRs.

## Results

### Identification, classification and characterization of plant *GLRs*

**Figure 1:**
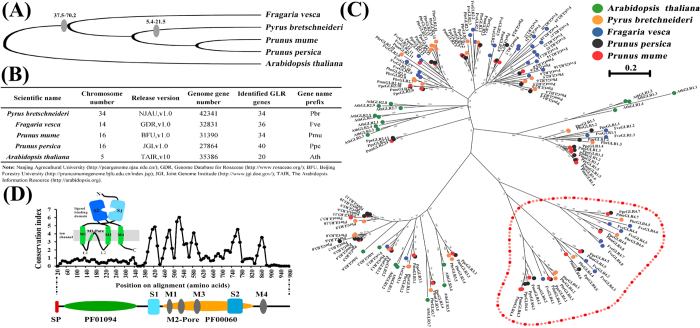
GLR gene family in four Rosaceae species. (**A**) Species tree of four Rosaceae species and *Arabidopsis*. The gray oval indicates the occurrence of WGD. Numbers in the figure show species divergence time. Unit: MYA. The data were downloaded from NCBI Taxonomy common tree (http://www.ncbi.nlm.nih.gov/Taxonomy/CommonTree/wwwcmt.cgi) and the tree was constructed by MEGA5. (**B**) Table of genome information and *GLR* genes number identified in four Rosaceae species and *Arabidopsis*. (**C**) Phylogenetic tree of four Rosaceae species and *Arabidopsis* GLRs protein. Roseceae GLRs have been given preliminary names is similar to those of *Arabidopsis*. Protein phylogenetic analysis using the Maximum Likelihood method in MEGA5 for 164 GLR amino acid sequences from pear (orange circular), strawberry (blue circular), plum (black circular), peach (red circular) and *Arabidopsis* (green circular). The sequences were aligned using MUSCLE. The bootstrap consensus tree was generated using the JTT matrix-based model with discrete gamma distribution [five categories (+G, parameter = 1.0524)] by MEGA5 from 1000 bootstraps. The scale bar is 0.2. The red dotted box shows that the subfamily group of Roseceae species more than *Arabidopsis*, we define it as GLR4 subfamily. (**D**) Histogram showing the mean conservation index (number of conserved physico-chemical properties) for sliding window (20 aa window, 10 aa slide) line chart of protein aligned from Rosaceae species and *Arabidopsis* GLRs. The protein domain organization of GLRs is shown in cartoon form above the histogram and in linear form below it.

There were 167 candidate *GLRs* identified in four Rosaceae species (Fig. 1A), and 23 were classified as pseudogenes because they contained only short gene fragments or contained frameshift mutations and/or premature stop codons. Consequently, 144 complete *GLRs* were used for analysis (Fig. 1B and Table S1). Phylogenetic analysis suggested that analogous to *Arabidopsis*, most Rosaceae *GLRs* were divided into three traditional phylogenetic clades (*GLR1*, *GLR2* and *GLR3*). Moreover, we observed a well-supported new clade (bootstrap values of 82–100%; Fig. 1C), independent of traditional clades, and named this clade as *GLR4* subfamily.

GLR4 members shared a similar modular organization to other Rosaceae and *Arabidopsis* GLRs (Fig. S1), consisting of a receptor family ligand binding region (PFAM ID: PF01094) and ligand-gated ion channel domain (PFAM ID: PF00060). In detail, it contained an extracellular ATD, four trans-membrane domains (M1-M4), a putative ligand-binding domain with two lobes (GlnH1 and GlnH2) that were separated by an ion channel domain, and a short cytoplasmic C terminus (Fig. 1D). Then, we aligned GLR4 proteins together with other subfamilies GLRs to examine the mean conservation index (number of conserved physico-chemical properties) using a sliding window (20 amino acid window, 10 amino acid slide) in four Rosaceae species and *Arabidopsis*. The results showed that the all annotated domains performed high conservation indexes, instead, the ATD (PF101904) was considerably more variable (Fig. 1D). The most conserved domain was the M2-Pore region (highest mean conservation index of 6.05), which determined the selective transduction of cations by their residues^29^. We generated a protein alignment for this region. The residues that determine the selective transduction of cations were highly uniform among Rosaceae and *Arabidopsis* GLRs included GLR4 subfamily (Fig. S2). Hence, GLR4 subfamily members are consistent with other Rosaceae GLRs that possess potential conserved plant GLR channel specificities. However, the principal issue of the GLR4 subfamily distribution in plant evolution needs to be addressed.

**Figure 2:**
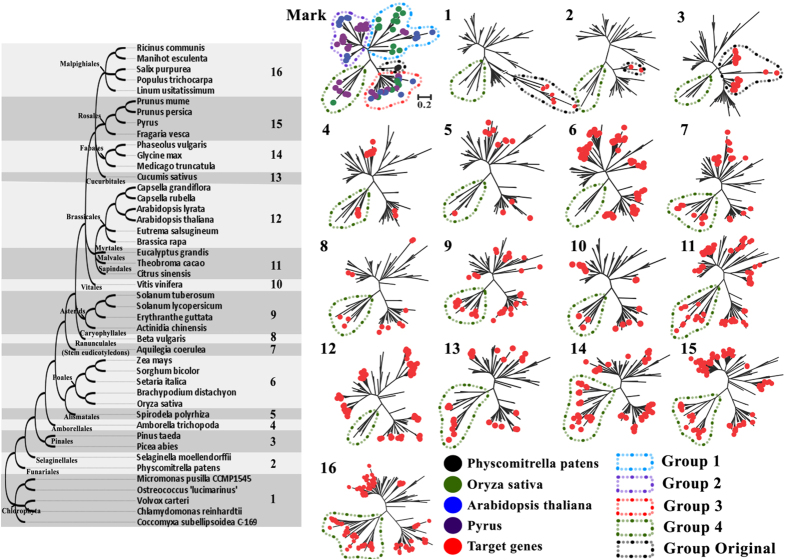
Phylogenetic analysis for plant GLRs evolutionary history. Protein phylogenetic analysis using the Maximum Likelihood, The sequences were aligned using MUSCLE. The bootstrap consensus tree was generated using the JTT matrix-based model with discrete gamma distribution by MEGA5 from 1000 bootstraps. An unscaled tree showing the phylogenetic relationships of 45 plant species was illustrated on the left, we divided this plant system into 16 parts according to the plant taxonomy. The right part is phylogenetic analysis for each part of plant species’ *GLRs*, we take moss (lower plant), rice (endogen plant), *Arabidopsis* (dicotyledonous herbs), pear (dicotyledonous woody) as marker genes to construct phylogenetic tree for each part plant species, then novel 881 sequences were collected and all plant GLR genes subfamily were classified. The scale bar is 0.2 for all phylogenetic trees. The non-circular labelled branches in 1~16 part phylogenetic trees are mark genes in Mark phylogenetic trees, all these mark genes in 1~16 part phylogenetic trees are location consistency with Mark phylogenetic trees.

For this purpose, we comparatively analysed *GLRs* in 45 plant species. We separated these plant species into 16 parts based on plant taxonomy (http://www.ncbi.nlm.nih.gov/Taxonomy/CommonTree/wwwcmt.cgi) and selected moss (lower plant), rice (monocotyledonous plant), *Arabidopsis* (dicotyledonous herbs), and pear (dicotyledonous woody) *GLRs* as anchors for construction of a phylogenetic tree to cluster the remaining plant *GLRs* (Fig. 2). Then, a collection of 881 sequences from 45 plant species was classified (Table S2). The *GLR4* genes were widespread in all analysed angiosperms. They first appeared in *Amborella*, which is the earliest known angiosperm species^30^. However, this subfamily has not been found in Brassicales (Fig. 2 and Table S2). This could explain the missing *GLR4* subfamily in the genome of *Arabidopsis*.

### Evolutionary history of plant *GLRs*

**Figure 3:**
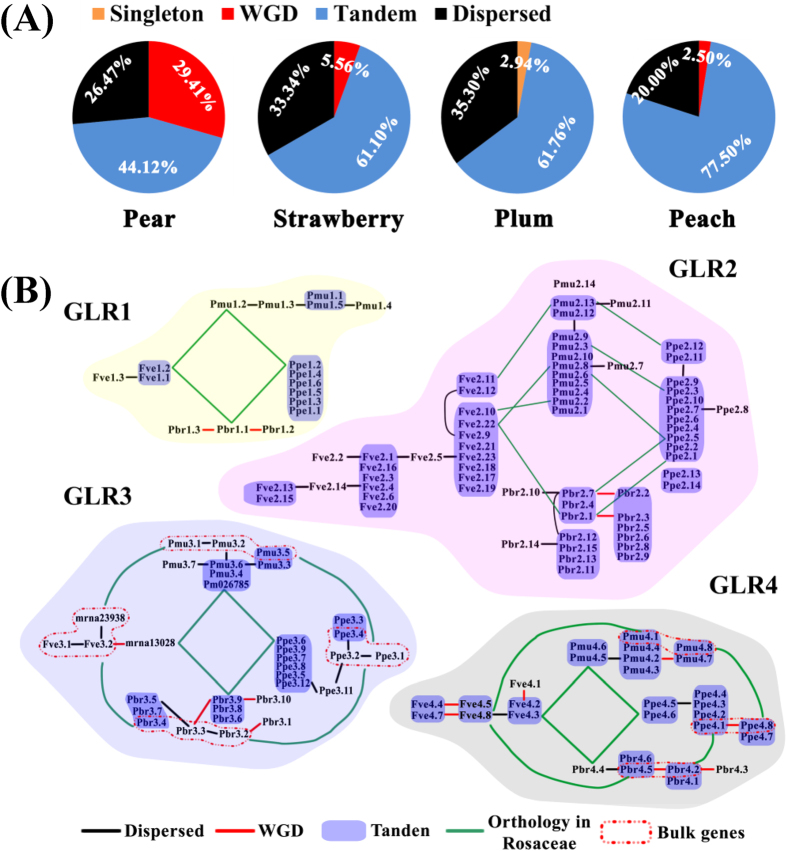
Expansion analysis of *GLR* gene family in Rosaceae genomes. (**A**) The quantification statistics for different duplication events in Roseceae *GLRs*. Initially, the BLASTP algorithm was used to search for potential homologous GLR gene pairs (E < 1 e^−5^, top five matches) across entire genome. Secondly, these homologous pairs were used and inputted into *MCScanX* to identify syntenic chains. The *GLRs* locate in a syntenic block were defined as WGD/segments duplication; The *GLRs* neighbouring less than 10 genes were defined as tanden duplication; and the remainders of homologous *GLRs* were defined dispersed duplication. (**B**) Orthology and duplicate path analysis of *GLR* genes in four Roseceae species. We assembled the duplicated gene pairs to form a linear expansion in each Rosaceae species based on hypothesis tests on the nearest duplicated gene pair’s performance characteristics, which included the highest identity (Table S4), maximum number of flanking genes in segments and the lowest synonymous mutations (Table S5). Orthology *GLRs* were identified in *MCScanX* program.

To investigate the evolutionary processes of plant GLR repertoires, we used Rosaceae *GLRs* as examples to determine the potential original, expansion and divergence of this gene family. First, we detected the duplication modes for *GLRs* in four Rosaceae genomes using the *MCScanX* package. Tandem duplications occupied the largest proportion of the *GLR* family’s expansion in Rosaceae (Fig. 3A, Fig. S3 and Table S3). Dispersed duplication was secondary contributor to expansion of this gene family in Rosaceae. Interestingly, the recent whole genome duplication (WGD) events (30–45 MYA) occurred in pear^27^ likely resulted in the observed higher proportion of WGD/segmental-type *GLR* gene duplications (Fig. 1A). Together, these observations support that *GLRs* evolved mainly from tandem duplications in Rosaceae. Then, we assembled the duplicated gene pairs to form a linear expansion in each Rosaceae species based on hypothesis tests on the nearest duplicated gene pair’s performance characteristics, which included the highest identity (Table S4), maximum number of flanking genes in segments and the lowest synonymous mutations (Table S5). Finally, we identified the ancestral genes of the Rosaceae *GLR* families by collinearity and synteny analyses (Table S6). We constructed a schematic diagram of the development history of the Rosaceae *GLR* family (Fig. 3B).

This schematic diagram illustrated that five series of Rosaceae ancestral genes, two bulk orthologous genes in *GLR3* and *GLR4* subgroups, that were ordinal linked in four species. In contrast, the *GLR2* subfamily did not complete an orthology series, but was substituted by a more abundant and shorter series among two or three Rosaceae species. Meanwhile, there were three fragmented *GLRs* (mrna13028 and mrna23938 in strawberry, and Pm026785 in plum) that participated in evolution of the Rosaceae *GLR3* subfamily, indicating that these genes contained frameshift mutations and/or premature stop codons after the duplication events. In general, Rosaceae *GLRs* were taken several series of ancestral genes as the anchors during expansion; however *GLR2* subfamily had undergone a gene elimination during evolution. Once ‘born’, new *GLRs* presumably further expanded by tandem duplications, revealing this as a tremendous driving force for Rosaceae *GLR* gene expansion.

**Figure 4:**
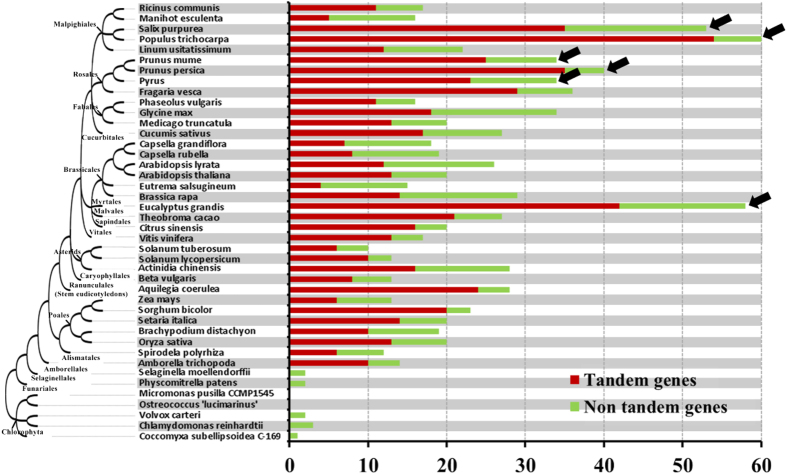
A broad number and tandem duplication event survey of GLR genes in plants. Histogram of the number of tandem genes (red) and non-tandem genes (green) sequences identified in the in the indicated plant species. An unscaled tree showing the phylogenetic relationships of 45 plant species was illustrated on the left. X axis indicated the number of genes. The black arrow indicated the highwood species in analysis plants.

Analysis of *GLR* evolutionary patterns over the entire plant timescale showed 581 *GLRs*, accounting for 65.95% of all analysed plant *GLRs*, were tandem-related genes (Fig. 4 and Table S7). It indicated that in all plant species, not just Rosaceae, tandem duplication is a major expansion force for *GLR* families. Exploring the evolutionary origin of plant *GLR*s by phylogeny analysis showed that the plant *GLR* family had an original group. We named this group *GLR0*, and it was closest to the *GLR3* group (Fig. 2). *GLR0* appeared first in algae, then began to differentiate and expand with evolution of Pinales, and finally differentiated into three stable and distinguishable subgroup structure, *GLR3*, *GLR4* and a larger and highly spread-out cluster (*GLR1* and *GLR2* subfamilies), from the Amborellales, the earliest angiosperm species known (Fig. 2).

### Plant *GLR1* and *GLR2* subfamilies formed sister clades

**Figure 5:**
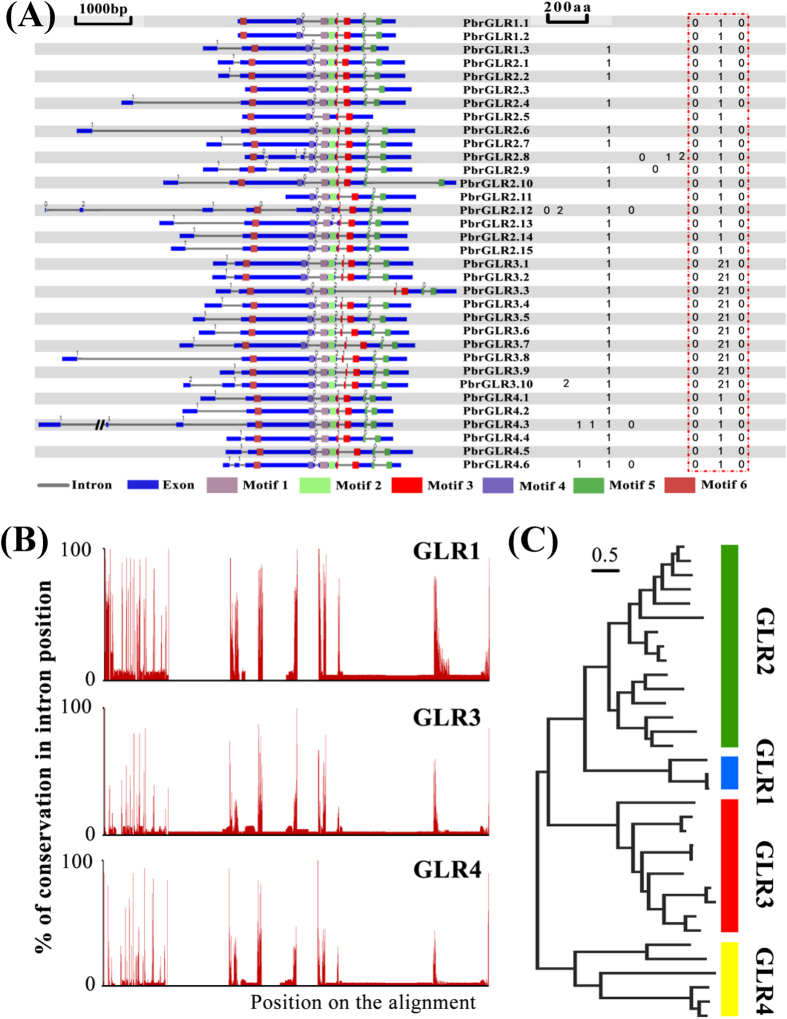
Evolutionary origin of pear GLRs. (**A**) Gene structure analysis of pear GLR genes using PIECE. The left part is pear GLRs gene structure performed by view gene structure in genomic sequences alignment with the main domain. 0, 1, 2 denote the phase of the intron. The blue part indicates the exons, the gray line indicates the intron and different colors indicate different motifs. The right part is gene structure performed by view type of gene structure in alignment protein sequences. The red dotted line represents conserved intron phase among all pear GLRs. **(B**) Map of the conserved intron sequences, which represents by red dotted line in (**A**), of pear *GLR2* subfamily with other pear *GLR* subfamilies. Selected intron sequences were aligned using PRANK, and the scattered sequences in the alignments were cleaned manually to obtain final high-quality alignments. (**C**) Phylogram based on the sequence of introns in the same subset of sequences as (**B**). Maximum likelihood tree was built by applying the JTT model with 1000 bootstraps in MEGA5.

Marker-based phylogenetic analysis shows that *GLR1* and *GLR2* combined into a distinguish cluster occurred in all of tested plant orders (Fig. 2), suggesting *GLR1* and *GLR2* subfamilies could be homologous and from a common gene. Consistent with the conservation analysis, *GLR2* had the most similar primary sequence to GLR1 in four Rosaceae species (Table S4). Taken pear *GLRs* as example, we tested the genes structure among subfamilies, the result showed the numbers of introns in *GLRs* were random (Fig. S1 and Table S1). Indeed, using the server GSDraw (Gene Structure Draw server) to detect the gene structure based on a genomic sequence alignment using the main domain, showing that these highly dynamic introns were concentrated at the N-terminus (Fig. 5A). However, the intron positions after motif 4 were conserved among pear *GLR1* and *GLR2* members (Fig. 5A). In addition, the highest identity value (43.12%) for the intron sequence after motif 4 between *GLR1* and *GLR2* subfamilies in pear (Fig. 5B). In comparison, there was only 25.37% identity between *GLR2* and *GLR3* subfamilies, and 20.23% identity between *GLR2* and *GLR4* subfamilies (Fig. 5B). Moreover, a phylogram of these intron sequences also revealed a greater similarity of *GLR2* to *GLR1* receptors than to other subfamily receptors (Fig. 5C). Likewise, similar results were obtained in other Rosaceae species (data not shown). Together, these observations demonstrated *GLR1* and *GLR2* formed sister clades and integrated into a single clade, which we named *GLR1&2*.

### *GLR1&2* subfamily has more tandem gene arrays accompanied by domain loss events

Comparative analysis of the evolutionary patterns for expansion of *GLR* subfamilies showed distinct characteristics for *GLR1&2*. The degree of tandem duplication events contributed to the expansion for *GLR1&2* and *GLR4* subfamily were similar, 75.67 and 76.02%, respectively, which significantly higher than the overall average value (65.95%) (Fig. S4 and Table S7). In contrast, tandem duplication genes only accounted for 44.44% of the *GLR3* subfamily and none of the *GLR0* subfamily. The results indicated that tandem expansion force was mainly in the *GLR1&2* and *GLR4* subfamilies. The average of 2.6 tandem gene arrays was significantly higher for the *GLR1&2* than the averages of 1.43 and 1.88 for *GLR3* and *GLR4*, respectively (Fig. S4). If duplicated genes had similar functions, then there were more functional differentiation events involved in the gene expansion process in the *GLR1&2* subfamily.

However, many domain loss events accompanied *GLR1&2* gene expansion. Surveying conserved domains showed that Rosaceae *GLRs* were missing one or more domains, with loss rates from 3.47% (GlnH1) to 15.97% (ATD) (Fig. S1 and Table S8). Among these, the missing ATD rate was significantly higher than those of other domains, especially in the strawberry. We further compared the domain loss rate in each *GLR* subfamily. The domain loss events in *GLR1&2* subgroups accounted for reach up to 78.57% of all loss events (Table S9). Meanwhile, the percentage lacking the ATD reached 11.03% in plant *GLRs*, with 74% in the *GLR1&2* subfamily (Table S10). Together, these clues indicated that during the expansion process, the *GLR1&2* subfamily incurred many domains loss event, especially in the ATD.

### Selective forces acting on the *GLR1&2* subfamily in stem eudicotyledonous species

**Figure 6:**
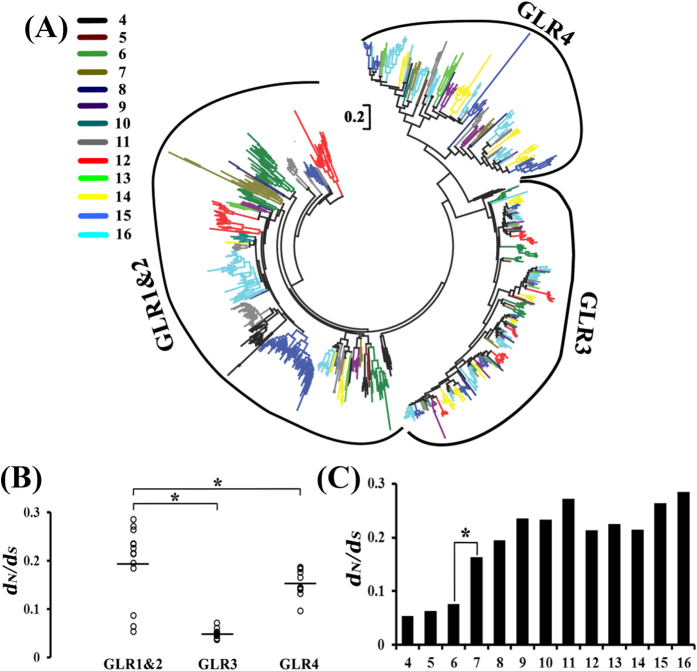
Species-specificity of *GLR1*&2 subfamily was evolved under selective forces. (**A**) Phylogenetic relationships of the all angiosperm GLRs shown in (Table S2). Each colour represents a part of GLR proteins as description in Fig. 2. The sequences were aligned with PRANK and the ML tree was built with MEGA5 under JTT matrix-based model with 1000 bootstraps. The scale bar is 0.2. *GLR1&2* subfamily showed a species-specific distribution in this phylogenetic analysis. (**B**) Distribution of selective forces of three GLR subfamilies estimated for all angiosperm species. Selective forces acting on plant *GLRs* by calculating the ratio of nonsynonymous to synonymous nucleotide substitution rates (*d*_*N*_*/d*_*S*_, ω). The median *d*_N_/*d*_S_ values of *GLR1&2* were significantly higher than *GLR3* and *GLR4* (p < 0.01, Wilcoxon rank-sum tests). The horizontal line representative the median *d*_N_/*d*_S_ values. (**C**) Histogram of *d*_N_/*d*_S_ rates of *GLR1&2* subfamily estimated for all angiosperm species which was devised into 13 parts as description in Fig. 2. The ω were remarkable elevated beginning from Ranunculales (p < 0.01, Wilcoxon rank-sum tests), which was the first stem eudicotyledonous order of plant species analysed.

*GLR1&2* subfamily showed a much broader distribution than other subfamilies (Fig. 2). Indeed, amino acid sequences of *GLR1&2* subfamily members had the lowest identities among the analysed plants (Table S11). Then, we reconciled the gene phylogeny by analysing *GLR1&2* in all the plant species. There were no obvious orthologous relationships for these genes among species, but a number of species-specific clades (Fig. 6A). Thus, we refer to the *GLR1&2* subfamily genes as species-specificity divergent GLRs.

We studied the selective forces acting on plant *GLRs* by calculating the ratio of nonsynonymous to synonymous nucleotide substitution rates (*d*_*N*_*/d*_*S*_, ω). All tested *GLR* subfamily genes evolved under strong purifying selection (ω ≪ 1) and the *GLR1&2* subfamily had the highest median ω of 0.194 (Fig. 6B), indicating that *GLR1&2* subfamily evolved under weaker purifying selection and/or contained more sites shaped by positive selection. To gain insights into species diversification of the *GLR1&2* subfamily, we compared ω of these genes in plant development lineage (Fig. 6C). Among these, Amborellales, Alismatales and Poales had the lowest ω of 0.053–0.096, consistent with their high sequence conservation levels in the plant lineage. Notably, the median ω in the following taxonomic tree was statistically higher than those of Amborellales, Alismatales and Poales. The ω were elevated beginning from Ranunculales (ω = 0.183), which was the first stem eudicotyledonous order of plant species analysed. Certain orders had more greatly diverged ω, such as Myrtales, Rosales and Malpighiales, which all are contain highwood species. Thus, we inferred that selective forces acting on *GLR1&2* were correlated with those of the stem tissue genes.

### GLR1&2 subfamily expansion in highwood species

In the present study, the majority of plant species contained ~20 GLR members. Moreover, all analysed highwood species (pear, peach, plum, salix, populous and eucalyptus) contained significantly more *GLRs* (Fig. 4), and we assessed contributions of subfamilies to *GLR* expansion in highwood species. The *GLR3* subfamily population was roughly equal in all species (Fig. S4). *Salix* and *Populous* had more *GLR4* genes than the other species. In contrast, all highwood species had more *GLR1&2* genes (Fig. S4), suggesting that this subfamily was the major contributor to expansion. Therefore, we inferred that the *GLR1&2* subfamily potentially regulated aspects of woody growth in highwoods that may be less developed in the other species.

### High transcript abundance of *GLR1&2* in cambium

**Figure 7:**
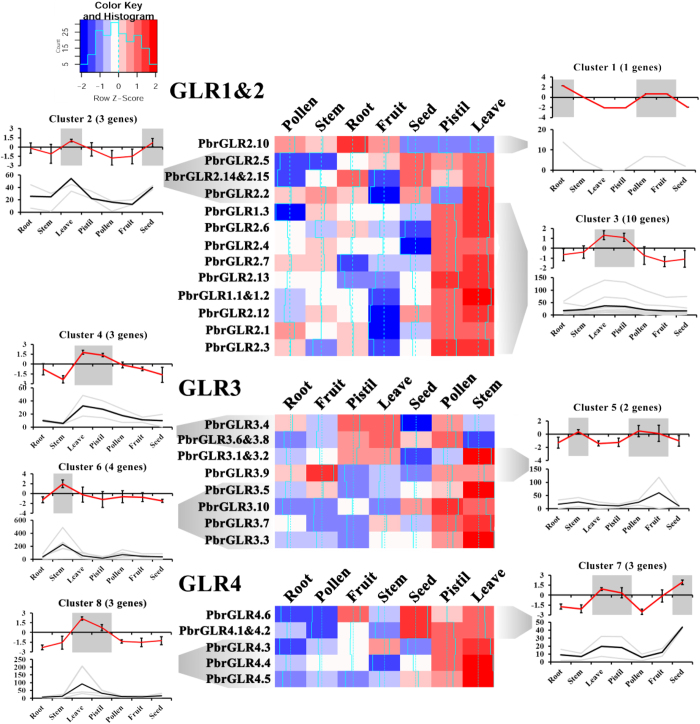
Heat map of the qRT-PCR transcript abundance pattern of the 31 *GLRs* from pear in 7 different tissues clustered in 7 expression groups using K-means. Genes and samples were hierarchically clustered according to their transcript abundance (expressed in relation to the mean of all samples and log2-transformed). For each gene, its name is shown to the left of the heatmap. Next to each cluster have two graphs that up graph with the mean transcript abundance (red line) base on standardized log_2_-relative expression value in RT-qPCR ± SD for the entire cluster, and down graph with the each genes relative expression value in RT-qPCR (gray line) and they mean transcript abundance value (black line) ± SD for the entire cluster. The Y-axis represents log_2_-relative expression value and relative expression value in RT-qPCR respectively. *PbrGLR2.8*, *PbrGLR2.9* and *PbrGLR2.11* could not be detected by RT-qPCR in 7 different tissues/organs, because their expression levels were too low to detect by normal RT-qPCR. There are 5 pair genes can’t be distinguish because of high identify in sequences. An amplified *PbrTub-2* and *PbrGADPH* were used as internal control.

To explore whether the *GLR1&2* subfamily was specifically expressed in the stem, we analysed expression profiles of the pear *GLR* family in seven tissues or organs using reverse transcription-quantitative real-time PCR (RT-qPCR). However, no *PbrGLR2.8*, *PbrGLR2.9* and *PbrGLR2.11* transcripts were detected. The remain 31 genes were classified into eight clusters based on transcript abundance patterns (Fig. 7). The gene family was predominantly expressed in leaves and pistils. Thirteen *GLRs* were highly expressed in stem tissue. Surprisingly, not only several of the *GLR1&2* genes but also other subfamilies genes were predominantly expressed in the stem tissue. We also analysed *GLRs* from poplar, another highwoody plant species, for RNAseq transcript abundance in six tissues (Fig. S5). The results were consistent with the observations in pear.

**Figure 8:**
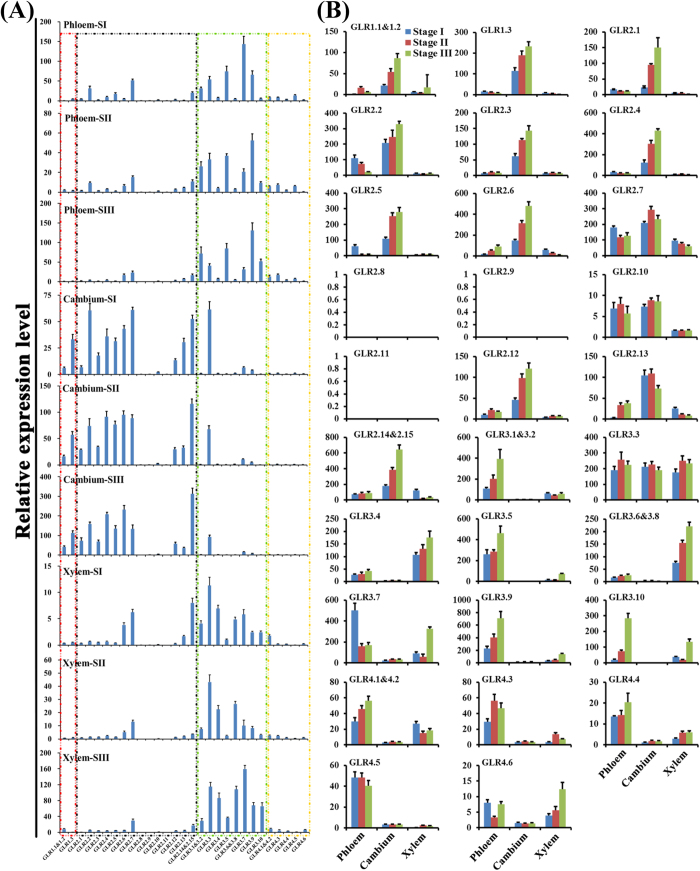
Temporal-spacial expression patterns of pear *GLRs* in stem vascular tissues by qRT-PCR. Xylem, phloem and cambium samples from three stages stems, which having increased lignification, were selected to determine different *PbrGLRs* expression levels. (**A**) The pear *GLR* gene family’s expression patterns in a single tissue. SI, SII and SIII denoted Stage I, Stage II and Stage III, respectively. Stage I: The stem above cotyledons from 90 days old pear tree; Stage II: The stem from the current growth branches of 8 years old pear tree. Stage III: The stem from the last year the branches of 8 years old pear tree. The stages were detail described in Fig. S5. The different color of dotted boxes were represented four subfamilies of pear *GLRs*. (**B**) The expression patterns of a single pear *GLR* gene in all analyzed vascular tissues. An amplified *PbrTub-2* and *PbrEF1α* were used as internal control. Samples were taken in at least triplicate reduplicate of biological, the average of which is show.

Indeed, to investigate specific temporal and spatial function of *GLRs* in stem, we selected xylem, phloem and cambium samples from three phases stems, which having increased lignification, to determine different *PbrGLR* expression levels (Fig. S6). *GLRs* expressions were grouped into three main patterns (Fig. 8), showing that *GLR1&2* members had nearly identical expression patterns, and were preferentially expressed in cambium. This was consistent with previous results showing that plant *GLR1* and *GLR2* subfamilies are sister clades. *GLR3* subfamily genes were preferentially expressed in phloem or xylem samples, and *GLR4* genes had relatively low transcript levels in analysed vascular tissues but were preferentially expressed in phloem. Indeed, *PbrGLRs*’ transcript abundance gradually increased in vascular tissues during lignification-enhanced stages. Coincidentally, we also found similar *GLR* expression patterns in hypocotyl from *Arabidopsis* which producing secondary xylem with vessels and fibres similar to those of woody species (Fig. S7A). *Arabidopsis GLR3* was prominently expressed in all phloem-type cells containing phloem companion, phloem pole pericycle, protophloem and metaphloem, and also in xylem cells. However, *GLR1* and *GLR2* of *Arabidopsis* were preferentially expressed in procambium cells. In addition, we found that most of *GLRs*’ expression levels significantly increased during *Arabidopsis* maturation (Fig. S7B). Poplar *GLRs* showed similar expression patterns to pear and *Arabidopsis* in phloem and xylem tissues, although we did not find available expression data for poplar cambium tissue (Fig. S8). These observations support the hypothesis that *GLR*s perform distinct specific temporal and spatial functional roles in vascular tissues, and that *GLR3* and *GLR4* genes participate in xylem and phloem, while *GLR1&2* genes is involved in the cambium.

Meanwhile, we detected transcript abundance profiles of *GLRs* during sprouting in the shoot tip tissues of pear and poplar. Nearly all pear *GLR1&2* genes that were highly expressed in cambium also showed increased expression in sprouting dormant shoot tips (Fig. S9), with similar results in poplar (Fig. S10). These results corroborate a possible dual role in meristematic modulation, and many genes can regulate both the shoot apical meristem and vascular cambium^31^,^32^.

## Discussion

### Evolutionary history of plant *GLRs*

Comprehensive survey of plant GLR genes allowed us to develop a model of their evolution (Fig. 2). Phylogenetic relationships, sequence similarities, gene structures and expression patterns suggested that *GLR1* and *GLR2* subfamilies were sister clades, thus we combined them into a common subfamily, *GLR1&2*. We hypothesized that *GLRs* originated from the *GLR0* plant subfamily, closely related to the *GLR3* subfamily, and that they subsequently diverged into the *GLR1&2*, *GLR3* and *GLR4* subfamilies, with the earliest occurring in Amborellales, which is the first angiosperm species^30^ (Fig. 2), estimated 160 million years ago. However, the *GLR4* subfamily was absent in Brassicales. Thus, if the *GLR4* subfamily evolved in a common ancestor of plant and existed in the ancient species, it must have subsequently been lost in Brassicales for an unknown reason.

### *GLR1&2* subfamily exhibited distinct characteristics

Comparative analysis of evolutionary patterns for *GLR* subfamily expansion in plant, revealed distinct characteristics for *GLR1&2*. Tandem duplications as the main driving force with a concomitant more tandem gene arrays were observed in expansion of this subfamily, indicating functional differentiation in the gene expansion process. Furthermore, low sequence identities, high selective forces and highly spread instead orthologous phylogenetic relationships of *GLR1&2*, suggested that this subfamily diverged in a species-specific pattern. The reason for this divergence is unknown, but it implies a reduced evolutionary constraint on co-expression, partially redundant ion channel genes undergo the selection for higher diversity in ion transport sequences to recognise more variable ligands in the environment. Indeed, the higher selective forces acting of *GLR1&2* subfamily start at stem eudicotyledonous plants and this subfamily members occurred remarkable extended in highwood plant species, inferring that the *GLR1&2* subfamily is potentially involved in regulating aspects of highwood woody growth.

Meanwhile, many plant GLRs had loss events in the ATD and 74% occurred in the GLR1&2 subfamily. The ATD is a discernible and divergent domain preceding the GlnH1 domain in the N-terminus and is involved in the assembly of GLR subunits into heteromeric complexes in animal iGluRs and *Arabidopsis* GLRs (Table S10)^2^,^7^,^33^. Moreover, expression of the lack-ATD gene (*PbrGLR2.11*) was not detected in any pear samples (Figs 7 and 8), indicating that the ATD lesion may be an elimination mechanism for gene redundancy in the *GLR1&2* repertoire. Additionally, 80.22% of this type of *GLR* was involved in tandem duplication (Table S2). Thus, these results led to the hypothesis that retroposition events eliminated the gene redundancy to meet the frequent tandem events. However, 3.23% of highwood *GLR* repertoires performed retropositioning, significantly lower than the overall 11.03% (Table S10). It highlighted that family expansion and retain more functional *GLRs* was required in highwood species. In this light, we excluded ATD-lacking *GLRs* and recounted the number of plant GLR members (Fig. S11). There were more distinctly for *GLR* members in highwood higher than other plant species. This addressed why herbaceous strawberry had so many *GLRs* (Fig. 4).

### *GLRs* perform distinct specialization functions in vascular tissues, and *GLR1&2* is involved in cambium activate functions

Although there is no direct experimental evidence that plants GLRs have a role in xylem, ample evidence demonstrates that transcription and post-transcription modification of key enzymes in the nitrogen assimilation pathway are influenced by the xylem flow of amino acids^34^,^35^,^36^. Moreover, many factors, such as light cycle, nitrogen supply and stress, can induce the amino acid profile changes in xylem sap, which is a continuum of the apoplasmic space^33^,^37^,^38^. It is tempting to assume that plant *GLRs* may be involved in Ca^2+^ signaling responses in amino acid profiles in the xylem. Coincidentally, results in this study corroborate previous work in expression profiles of *Arabidopsis*, poplar and pear (Fig. 8, Figs S7 and S8), in which *GLR3* genes were preferentially expressed in phloem or xylem cells, indicating that GLR3 genes could be involved in regulation of long-distance signaling in the vasculature, particularly phloem and xylem.

Preferential expression of *GLR1&2* in cambium cells is a plausible explanation for why *GLR1&2* channels function in the cambium and promote wood formation. In this scenario, this is a feedback loop invoked to expand the *GLR1&2* subfamily in highwood species. In fact, over their long lifespans, highwood plants require more sophisticated mechanisms and more genetic redundant-copy control to adapt to seasonal variations and expansive length of cambial activity, and to separate the juvenile and mature wood during secondary tree growth. This does not occur in vascular transmission regions, such as phloem and xylem, nor in short-lived annuals like *Arabidopsis*. Thus, it also aids understanding why highwood plants contain a high percentage of retained tandem repeats during evolution for the purpose of responding to variety of environmental stimuli^39^. Indeed, a possibility is that cambial activation through GLR channels affects wood development via Ca^2+^ signaling in the cambium. The functions of *GLR1&2* subgroups should, however, be further investigated using reverse genetic approaches in woody model plants to characterize their roles.

Studies conducted thus far suggest that GLR subfamily genes perform distinct specialized functions in vascular tissues. *GLR3* and *GLR4* having a role in ion transport in xylem and phloem, and *GLR1&2* in the activate functions of cambium.

### Co-expression is attributed to functional diversity via hereotetramer in plant *GLRs*

The resolved crystal structures of animal iGluRs are well characterized and unequivocally show that functional ligand-gated channels are formed from either four heterologous or homologous subunits within the same agonist class^40^. Although the picture of subunit composition in plant *GLRs* is far from complete, a preliminary analysis of *GLR* compositions is being elucidated. Co-expression analysis using single-cell sampling documented at least five *GLRs* co-expressed in *Arabidopsis* leaf epidermal or mesophyll cells^41^. Therefore, heterologous tetramer formation is quite likely. Moreover, a biophotonic assay demonstrated that *AtGLR3.2/AtGLR3.4* heteromeric interactions were stronger than homomeric interactions. Additionally, the heterogenous expression of *AtGLR3.2/AtGLR3.4* channels in human embryonic kidney cells resulted in a more sensitive current voltage compared with *AtGLR3.4*-only channels^21^. These results corroborate the previous hypothesis. In this scenario, the large scale of plant *GLR* co-expression in vascular tissues (Fig. 8, Figs S7 and S8) should be considered a model mechanism to defend against various environmental stresses via incorporation of a third or fourth species in the tetramer, which creates the type of highly dynamic functional diversity attributable to hereotetramer in plant *GLRs*. In this hypothesized mechanism, having different subunits strengthens the complex.

We analysed the genomes of 45 plant species to determine the comprehensive evolution of *GLRs*. This led to identification of the *GLR1&2* subfamily expansion in the highwood plants analyzed by tandem duplication. Moreover, species-specificity and selective forces shaped the *GLR1&2* repertoires’ distinct functional preferences in the stems of eudicotyledons. Indeed, expression studies showed that this subfamily was preferentially expressed in the cambium. This study represents a starting point for characterization of newly described GLR functions to better understand their roles in plants, especially highwood species.

## Materials and Methods

### Gene identification and annotation

Genomic and available annotated protein databases for pear (*Pyrus bretschneideri*) were downloaded from the pear genome project (http://peargenome.njau.edu.cn/). Conifer data were downloaded from the conifer genome project (http://congenie.org/). Data for other plant species were downloaded from Phytozome (http://phytozome.jgi.doe.gov/pz/portal.html). Multiple strategies were used to search for members of the GLR family in plants. First, the keyword ‘Glutamate Receptor’ was used to search available annotated protein databases. Secondly, a Hidden Markov Model (HMM) search was performed with the GLR domain’s HMM profile (PF00060), which has a ligand-gated ion channel-based function for the GLRs, and all significant hits (HMMER E value < e-5) were subsequently used as query. Finally, *A. thaliana* GLR protein sequences were used as queries in exhaustive BLAST algorithm-based searches with standard parameters until convergence occurred for each plant species. All identified sequences (below an arbitrary threshold E value < e-5) were then used as queries in TBLASTN searches of genomic DNA databases. Furthermore, all of the obtained GLR protein sequences were again analysed in the InterProScan database (http://www.ebi.ac.uk/interpro/search/sequence-search)^42^ to verify the presence of ligand-gated ion channel domains, and protein sequences lacking the PF00060 domain were removed.

### Phylogenetic analyses

#### Protein tree building

The selected GLRs’ amino acid sequences were aligned using MUSCLE^43^ and examined in Jalview^44^. ProtTest^45^ was used to evaluate the best model of substitution to infer the optimal phylogeny. The trees were calculated with MEGA5^46^, applying the best model of amino acid substitution. Approximate likelihood ratio tests were used for estimating bootstrap values. The trees were viewed and graphically edited with Mesquite, FigTree or Firework.

### Intron sequence tree building

Selected intron sequences were aligned using PRANK^47^, and the scattered sequences in the alignments were cleaned manually to obtain final high-quality alignments. A maximum likelihood tree was built by applying the JTT model with 1000 bootstraps.

### Taxonomy tree building

The taxonomy tree of analysed plant species was obtained by downloading data from the NCBI (http://www.ncbi.nlm.nih.gov/). The tree was constructed using MEGA5.

### Chromosomal locations and gene structures of Rosaceae *GLRs*

The chromosomal location of *GLRs* was obtained from Rosaceae genome annotation documents. Then, the data were plotted using Circos software^48^. The gene structures of the Rosaceae GLRs were drawn by Gene Structure Display Server (http://gsds.cbi.pku.edu.cn/). Website Plant Intron Exon Comparison and Evolution database^49^ (PIECE, http://wheat.pw.usda.gov/piece/FAQ.php) was used to conduct a motif analysis of the Rosaceae GLR genes and proteins. The figure, with six motifs, was plotted using coding sequences against the genomic sequence.

### Duplication model and synteny analysis

The analysis of synteny among the four Rosaceae genomes was conducted locally using a method analogous to that developed for the plant genome duplication database (http://chibba.agtec.uga.edu/duplication/)^50^. Initially, the BLASTP algorithm was used to search for potential homologous GLR gene pairs (E < 1 e^−5^, top five matches) across multiple genomes. Secondly, these homologous pairs were used and inputted into *MCScanX*^51^ to identify syntenic chains among the four Rosaceae genomes. Further, *MCScanX* also was used to identify singletons, WGD/segments, and tandem and dispersed duplications in the *GLR* families. Then, the information from the duplication model was plotted using Circos software.

### Calculating *d*_*N*_/*d*_*S*_

We estimated the *d*_*N*_*/d*_*S*_ ratio (ω) using maximum likelihood as performed in PAML^52^. All PAML analyses were implemented three times using different input parameters to refrain from local optima. We used MUSCLE to create multiple amino acid sequence alignments of orthologous genes. Then, these results were used to guide the nucleotide coding region alignments implemented by custom-written software. The columns with gaps were deleted for ω calculations. We selected model M0 to calculate the global selective pressure acting on the different subfamilies of *GLRs* based on their phylogenetic tree.

### RNAseq expression analysis

RNAseq data of *Arabidopsis* and poplar were obtained from the *Arabidopsis* eFP Browser (http://bar.utoronto.ca/efp/cgi-bin/efpWeb.cgi) and PopGenIE (http://popgenie.org/), respectively, except for the expression data of poplar xylem, which was obtained from the Poplar eFP Browser (http://bar.utoronto.ca/efppop/cgi-bin/efpWeb.cgi). Absolute expression data were normalized by the GeneChip operating software method in these databases, with a target intensity value of 100. Most tissues were sampled in triplicate. Then, these values were log_2_ transformed and standardized using EXPANDER 6 53. The K-means clustering in EXPANDER 6 grouped the genes into different main clusters with unique expression patterns.

### RT-qPCR

The confirmation of primers were shown in Table S12. Total RNA was extracted using TRIzol reagent (TaKaRa, Japan) according to the manufacturer’s method. RNA samples were assessed with OD 260/280 > 2.0 and OD 260/230 > 1.8. Equal amounts of total RNA (2 μg) from all of the samples were treated with DNase I to eliminate genomic DNA contamination and were then used for cDNA synthesis with a PrimeScript^TM^ RT Reagent Kit (Perfect Real Time; TaKaRa). Purified cDNA samples were diluted 1:20 with RNase-free water before use as templates in the RT-qPCR process. RNA extraction and cDNA synthesis from all of the samples was performed with four biological replicates.

Then, 20 μl solution system, corresponding to 5 ng of total cDNA, was used in per PCR (TaKaRa SYBR PrimeScript RT-PCR Kit for Perfect Real Time). The reaction was carried out in a RT-qPCR cycler (Roche LightCycler^®^ 480II) according to the manufacturer’s method (TaKaRa). Reaction mixtures were incubated for 10 min at 95 °C for pre-incubation, followed by 45 amplification cycles of 15 s at 9 °C, 15 s at 60 °C and 20 s at 72 °C. After that, a dissociation curve was generated (at 60–95 °C) to check the specificity of the amplicon. Three biological replicates were amplified for all samples. Lin-RegPCR was used to calculate the efficiency of the RT-qPCR primers. An electrophoresis analysis of the PCR product sizes was also carried out to determine their amplicon specificity. The expression levels of the *GLRs* in all samples were determined by their quantification cycle values (*C*qs). Then, these expression values transformed into log_2_ values and were standardized using EXPANDER 6. The K-means clustering in EXPANDER 6 grouped the genes into different main clusters with unique expression patterns. An MIQE checklist contains the essential information about qPCR analysis in this study provided (MIQE checklist). All indexes met the MIQE guidelines^54^.

### Plant growth conditions

All materials were collected from *P. bretschneideri* Rehd. cultivar ‘Dangshansu’ growing in a natural environment in Nanjing, China. The harvested seeds were germinated for 3 d and transferred to pots containing soil and vermiculite. Roots, stems and leaves were harvested 90 d after transfer. The mature seeds were obtained 13 weeks after flowering. Flowers were harvested a few days before anthesis, and the styles were then detached, weighed and stored in liquid nitrogen. Anthers were also dehisced, dried in bottles containing desiccant, and stored at −20 °C in coated silica gel until needed. Mature pear pollen was cultivated in liquid for germination and growth. The incubation medium contained the following components (mM): 5 2-(*N*-morpholino) ethanesulfonic acid hydrate, 440 sucrose, 0.55 Ca(NO_3_)_2_, 1.60 MgSO_4_, 1.60 H_3_BO_3_, and 1.00 KNO_3_ at pH 6.0–6.2 (adjusted with Tris). The pollen was incubated in small Petri dishes at 25 ± 1 °C for 3 h. Then, the samples were centrifuged, the supernatants removed and the pollen tube precipitates frozen in liquid nitrogen. The three typical stages of phloem, cambium and xylem samples were described in Fig. S6. Stage I: stem above cotyledons from 90-d-old pear trees; Stage II: stem from the current growth branches of 8-year-old pear trees. Stage III: stem from last year’s branches of 8-year-old pear trees. All shoot tip tissues were harvested from 8-year-old pear trees and divided into two phases; dormant (February) and sprouted (April). All materials harvested were used for extraction of total RNA.

### Data access

Genomic and available annotated protein databases for pear (*Pyrus bretschneideri*) was downloaded from the pear genome project (http://peargenome.njau.edu.cn/). Conifers were downloaded from conifer genome project (http://congenie.org/). The other plant species were downloaded from the sources phytozome (http://phytozome.jgi.doe.gov/pz/portal.html). And also, the accession number was listed at the supplemental data document (Table S2).

## Additional Information

**How to cite this article**: Chen, J. *et al*. Evolutionary and Expression Analysis Provides Evidence for the Plant Glutamate-like Receptors Family is Involved in Woody Growth-related Function. *Sci. Rep*. **6**, 32013; doi: 10.1038/srep32013 (2016).

## Supplementary Material

Supplementary Information
