# Supplementary material for: Evolutionary and Expression Analysis Provides Evidence for the Plant Glutamate-like Receptors Family is Involved in Woody Growth-related Function
Source: Sci Rep. 2016 Aug 24;6:32013. doi: 10.1038/srep32013 (PMC4995503; doi:10.1038/srep32013)
Supplement: Supplementary Information [file srep32013-s1.pdf]

Evolutionary and Expression Analysis Provides Evidence for the Plant Glutamate-like Receptors Family is Involved in Woody Growth-related Function

Jianqing Chen[1†], Yinghui Jing[1†], Xingyue Zhang[1], Leiting Li[1], Peng Wang[1], Shaoling Zhang[1], Hongsheng Zhou[2], Juyou Wu[1]*

† Equal contributors

* Corresponding author

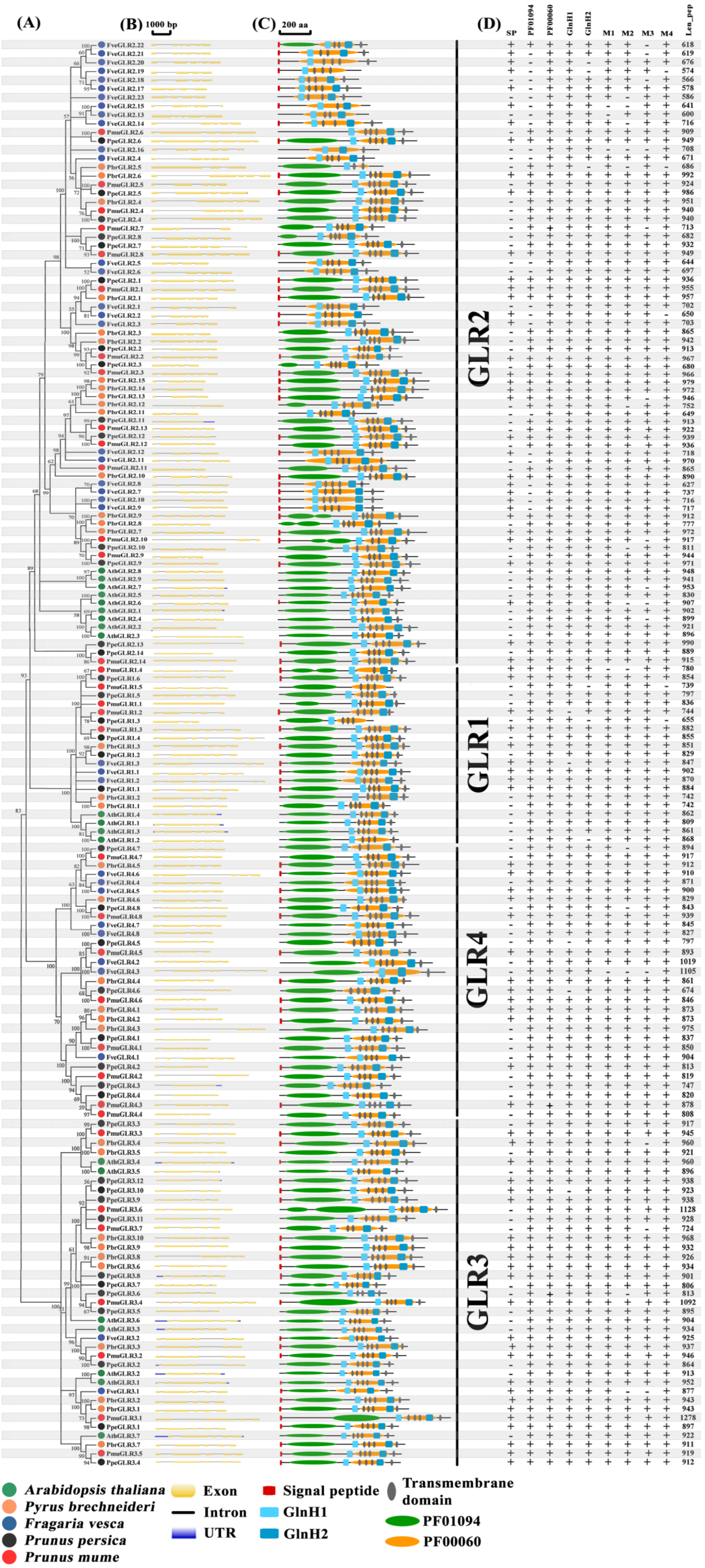
(A)
(B) 1000 bp
(C) 200 aa
(D)
SP
PF01094
PF00060
GlnH1
GlnH2
M1
M2
M3
M4
Len_pep
GLR2
GLR1
GLR4
GLR3
Arabidopsis thaliana
Pyrus brechneideri
Fragaria vesca
Prunus persica
Prunus mume
Exon
Intron
UTR
Signal peptide
GlnH1
GlnH2
Transmembrane domain
PF01094
PF00060

Fig. S1 Domain and gene structures analysis of GLR genes in Rosaceae and *Arabidopsis*.

(A) Phylogenetic relationship of GLRs protein in Rosaceae and *Arabidopsis.* using the Maximum Likelihood method in MEGA5 for 164 GLR amino acid sequences from pear (orange circular), strawberry (blue circular), plum (black circular), peach (red circular) and *Arabidopsis* (green circular). The sequences were aligned using MUSCLE. The bootstrap consensus tree was generated using the JTT matrix-based model with discrete gamma distribution [five categories (+G, parameter=1.0524)] by MEGA5 from 1000 bootstraps. **(B)** Schematic diagram for gene structures of *GLRs* in Rosaceae and *Arabidopsis.* The gene structures were drawn by Gene Structure Display Server (http://gsds.cbi.pku.edu.cn/). **(C)** Schematic diagram for conserved domain structures of GLR proteins in Rosaceae and *Arabidopsis.* The conserved domains were obtained from the protein alinement and InterProScan5 program search. **(D)** Statistics on conserve domain for each GLR protein in Rosaceae species and *Arabidopsis*. '+' expressed as domain appeared on the protein, '-' expressed as domain absented on the protein. Len_pep expressed as length of GLR protein (aa).

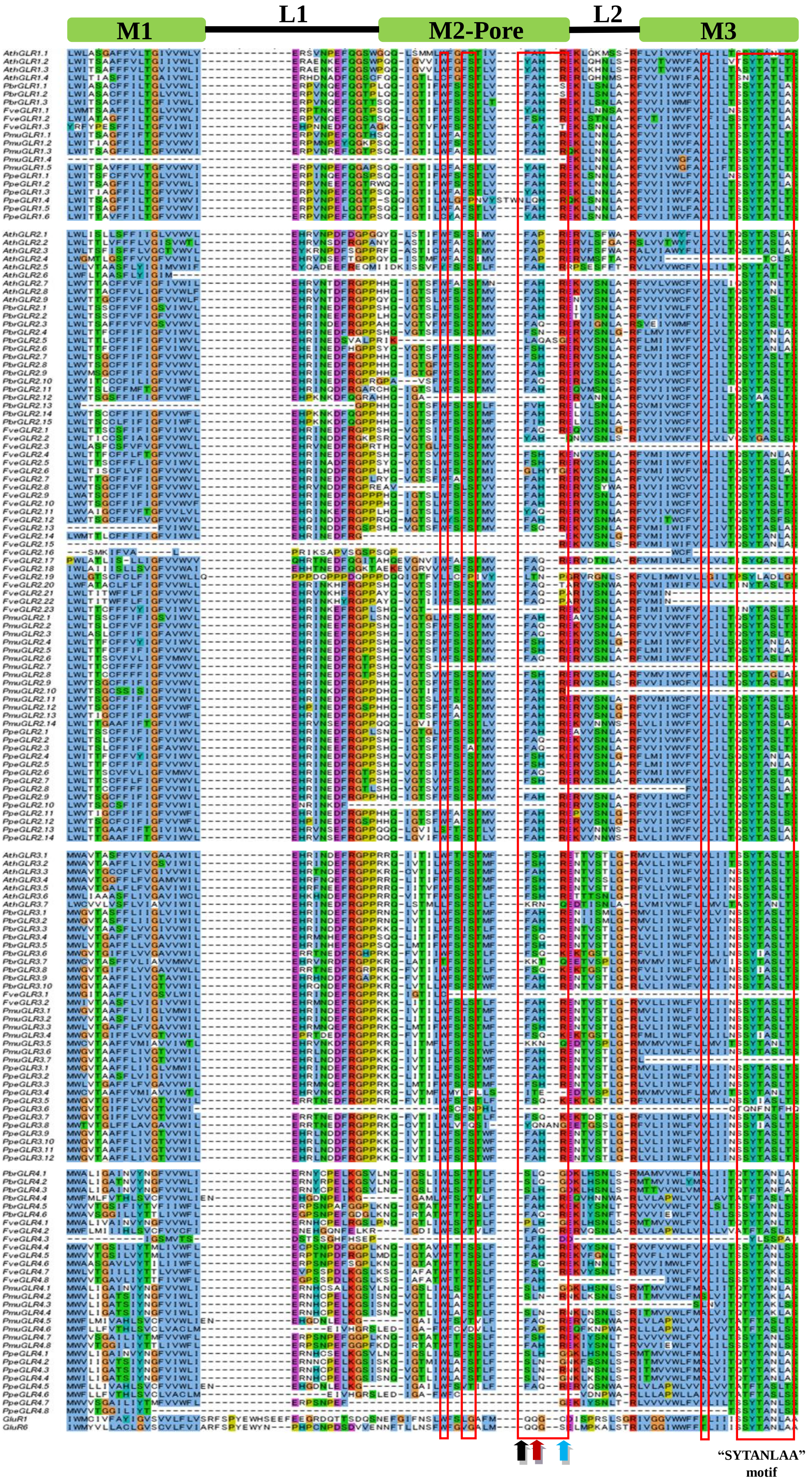
M1
L1
M2-Pore
L2
M3
"SYTANLAA"
motif

Fig. S2 Alignments of the amino acid sequences about ion channel domain from four Roseceae species, *Arabidopsis* GLRs as well as two rat iGluR subunits GluR1 and GluR6, which served as acceptors for pore transplantation in animal. The sequences were aligned using MUSCLE. The sex red outlined boxes represented channel-lining positions as determined for AMPA receptors. The red arrow denotes the narrow constriction of the channel, the black arrow denotes the tip of the pore loop (Q/R site), and the blue arrow denotes an important position on the internal side of the narrow constriction. The residues that determine the selective transduction of cations were highly uniform among Rosaceae and *Arabidopsis* GLRs included GLR4 subfamily

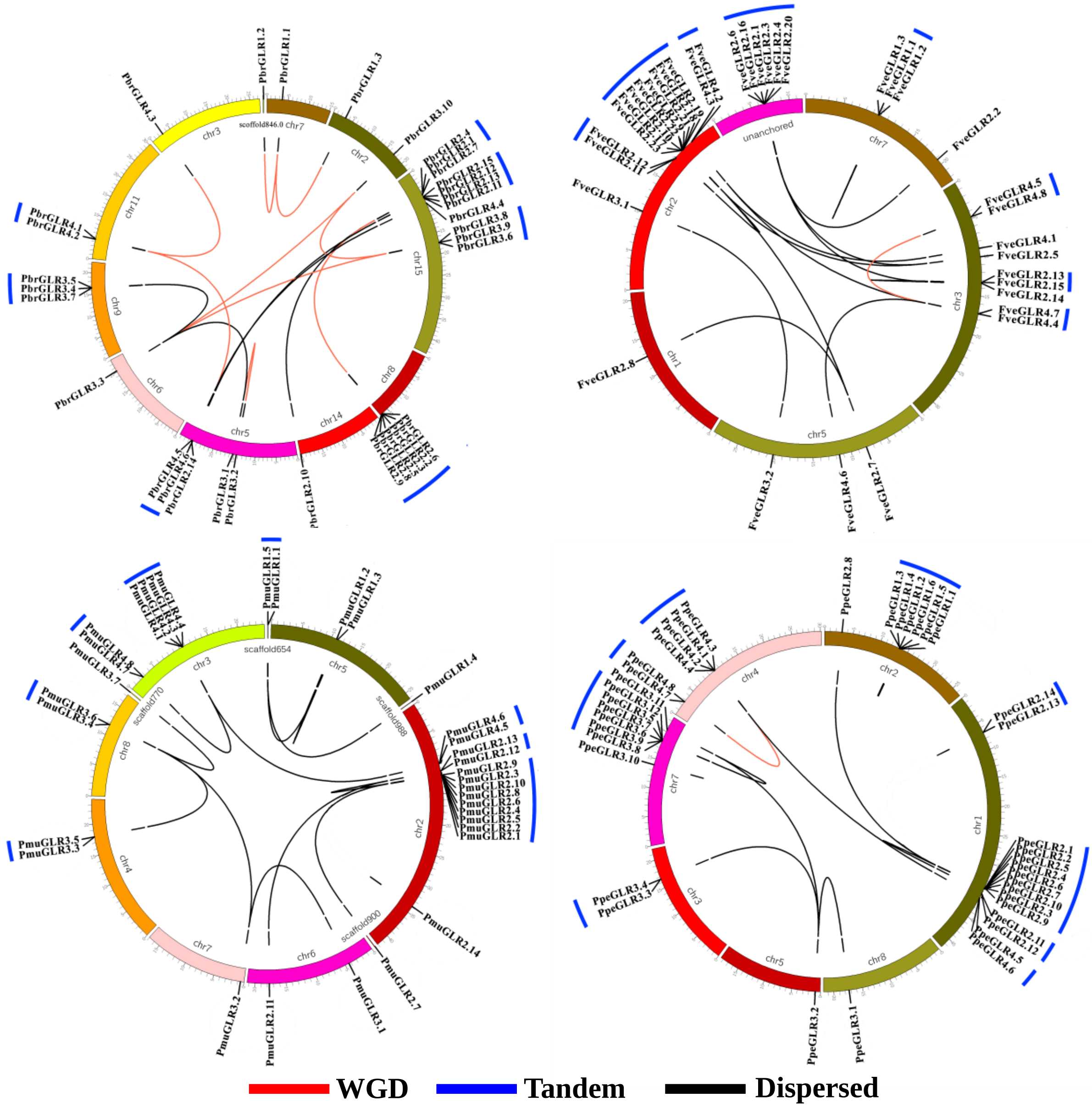

Fig. S3 Localization and duplication model of the *GLRs* in four Rosaceae genomes. *MCScanX* was used to identify singletons, WGD/segments, and tandem and dispersed duplications in the *GLR* families. Then, the information from the duplication model was plotted using Circos software.

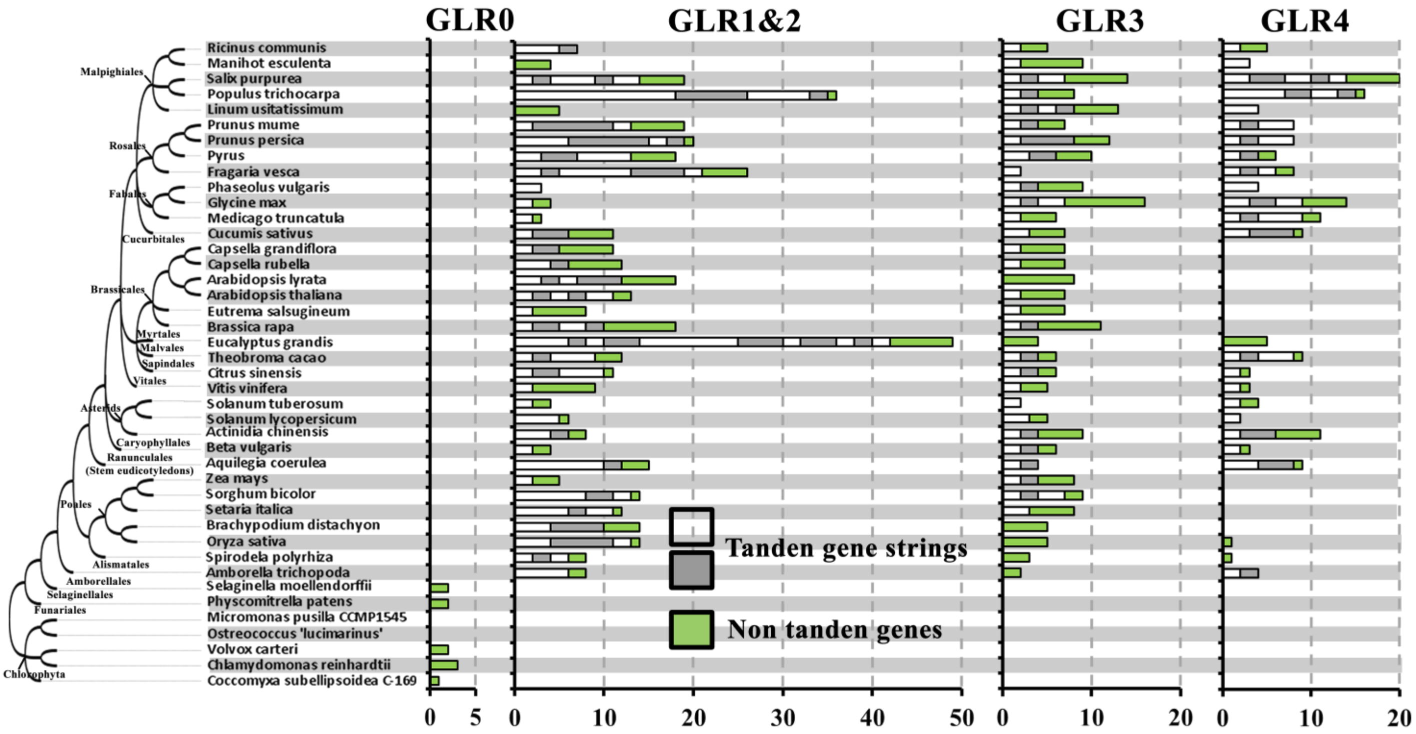

Fig. S4 A broad number and tandem duplication event survey of plant GLR genes in subfamily.

Histogram of the number of tandem genes (white and gray) and non-tandem genes (green) sequences identified in the indicated plant species, each box with white or gray represents a tandem gene string, to alternate with these two colors to improve the visibility of each tandem gene strings. An unscaled tree showing the phylogenetic relationships between these species is illustrated on the left.

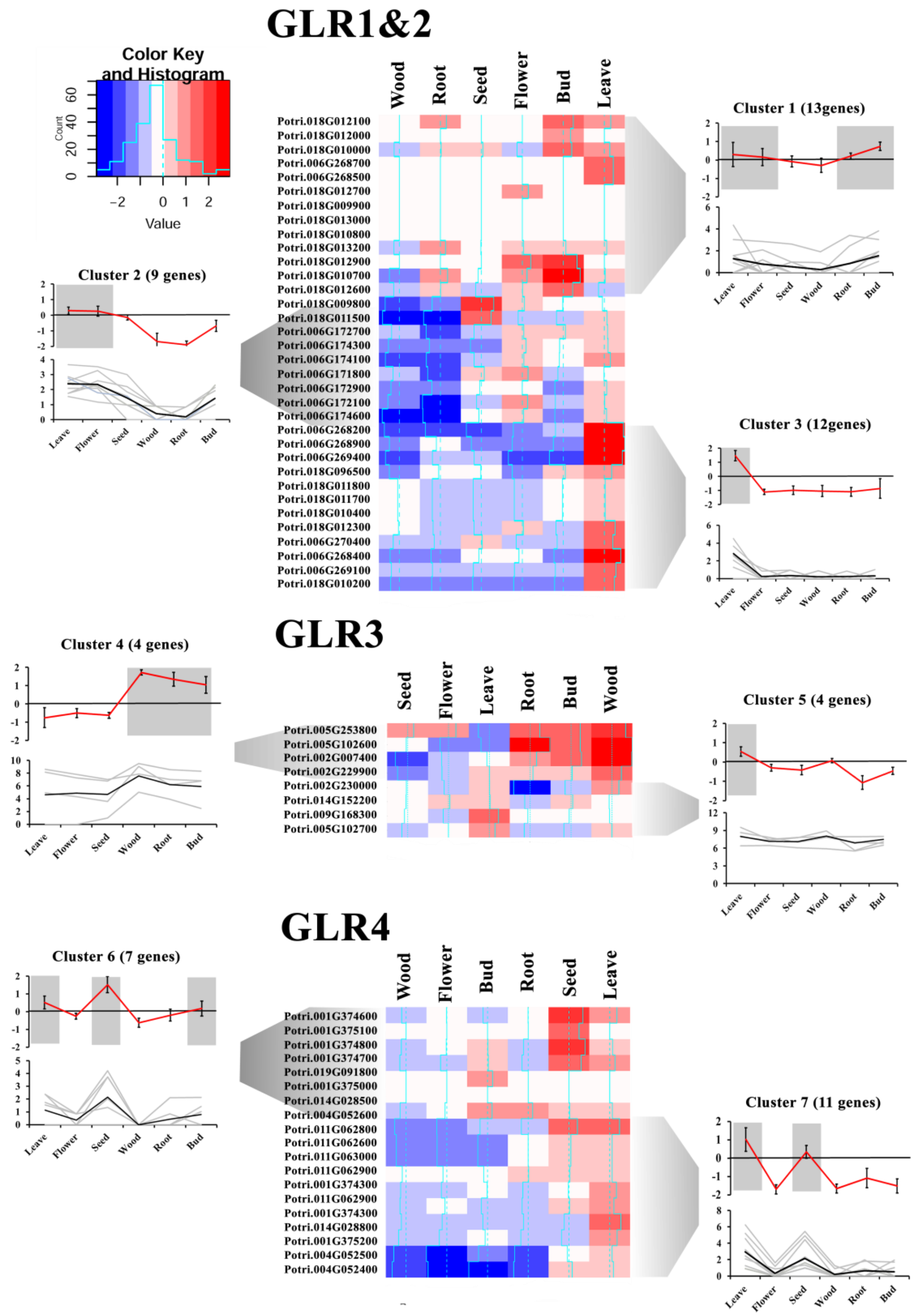

Fig. S5. Heat map of the RNAseq transcript abundance pattern of the 60 GLR genes from poplar in 6 different tissues clustered in 7 expression groups using K-means.

Genes and samples were hierarchically clustered according to their transcript abundance (expressed in relation to the mean of all samples and log2-transformed). For each gene, its

name is shown to the left of the heatmap. Next to each cluster have two graphs that up graph with the mean transcript abundance (red line) base on standardized log2-relative expression value in RNAseq ± SD for the entire cluster, and down graph with the each genes normalized expression value in RNAseq (gray line) and they mean transcript abundance value (black line) ± SD for the entire cluster. The Y-axis represents log2-relative expression value and normalized expression value in RNAseq respectively.

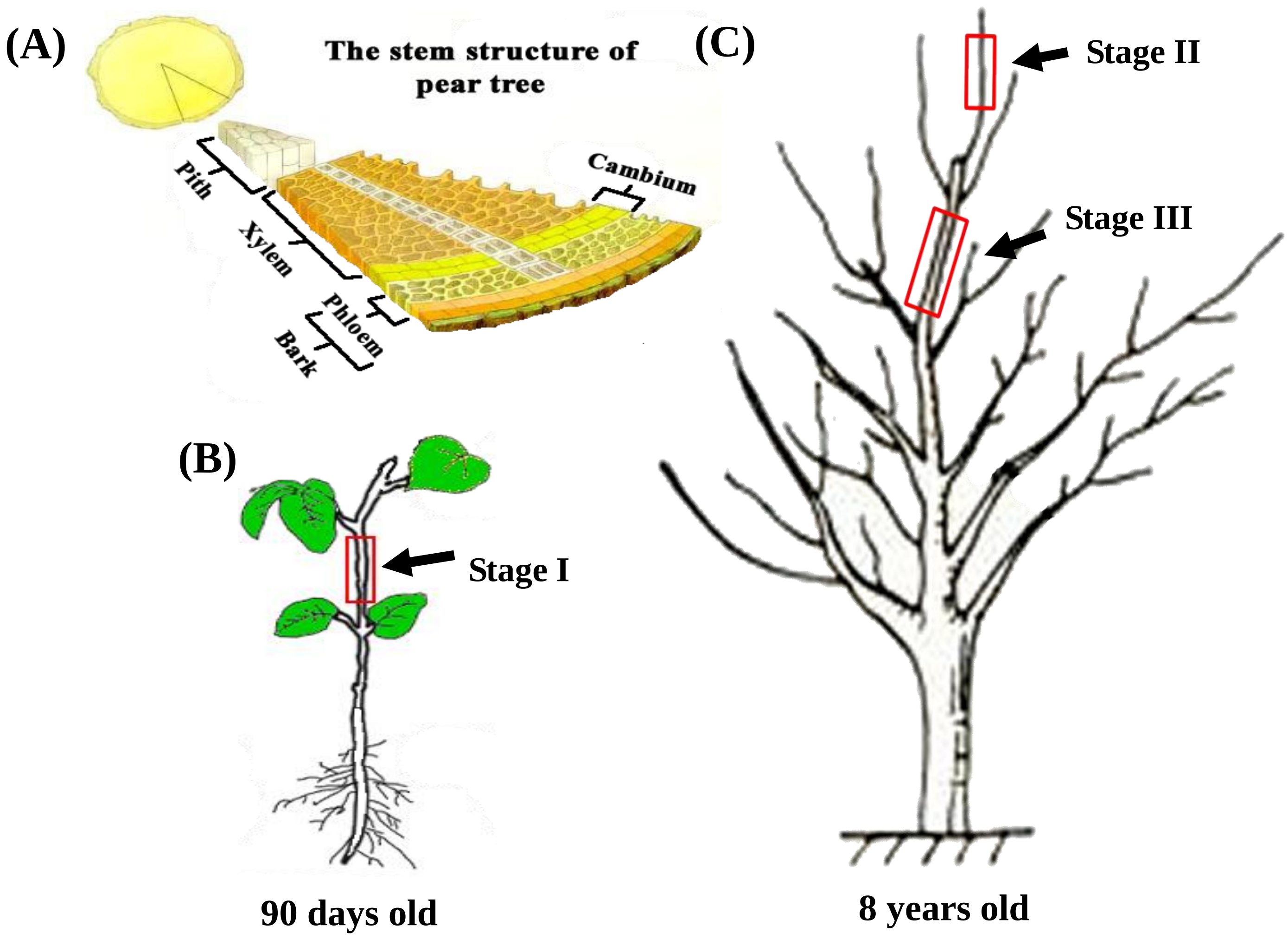

Fig. S6 Schematic diagram for selected samples in pear tree.

(A) Schematic diagram for the stem structure of pear tree and we choose three tissues, phloem, cambium and xylem, as targets were analyzed in this study. The selected of three typical stages samples were described in **(B)** and **(C)**. Stage I: The stem above cotyledons from 90 days old pear tree; Stage II: The stem from the current growth branches of 8 years old pear tree. Stage III: The stem from the last year the branches of 8 years old pear tree.

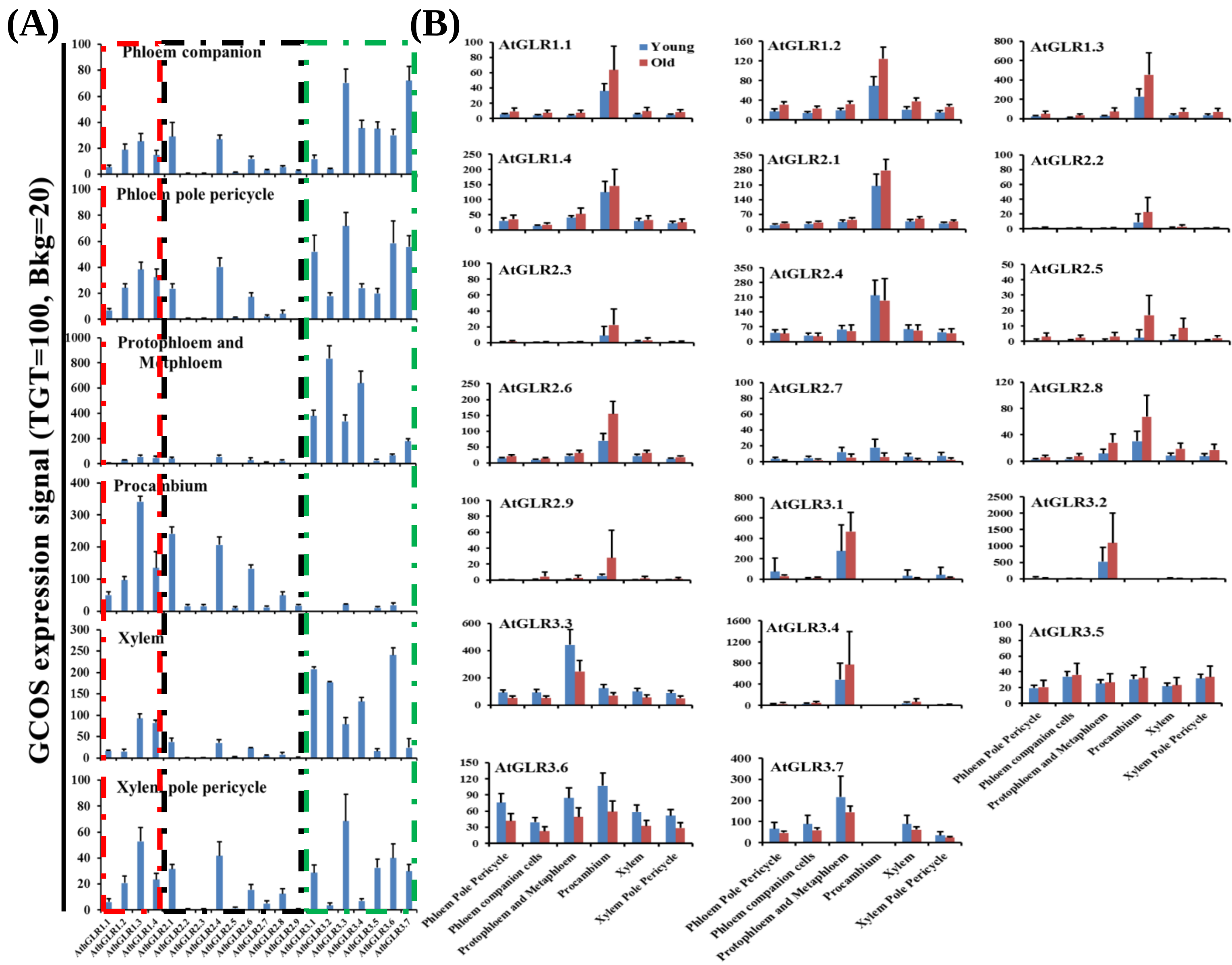

Fig. S7 Expression patterns of the *Arabidopsis* GLR gene family during vascular tissue system.

(A) Expression pattern of GLR genes with in six different vascular tissues, (phloem pole pericycle, phloem companion cells, protophloem&metaphloem, procambium, xylem and xylem pole pericycle) of *Arabidopsis***.** The different color of dotted boxes were represented three subfamily of GLRs. (B) For each gene transcript abundance pattern of the GLR genes form *Arabidopsis* in six different vascular tissues during young and old stages. Young stages at average levels in sections 1-6, old stages at average levels in sections 7-12. Expression data for selected genes for specific organs were retrieved from *Arabidopsis* eFP Browser (http://bar.utoronto.ca/efp/cgi-bin/efpWeb.cgi). ATH1 data are normalized by the

GCOS method, TGT value of 100. Samples were mostly taken in duplicate or triplicate, the average of which is shown.

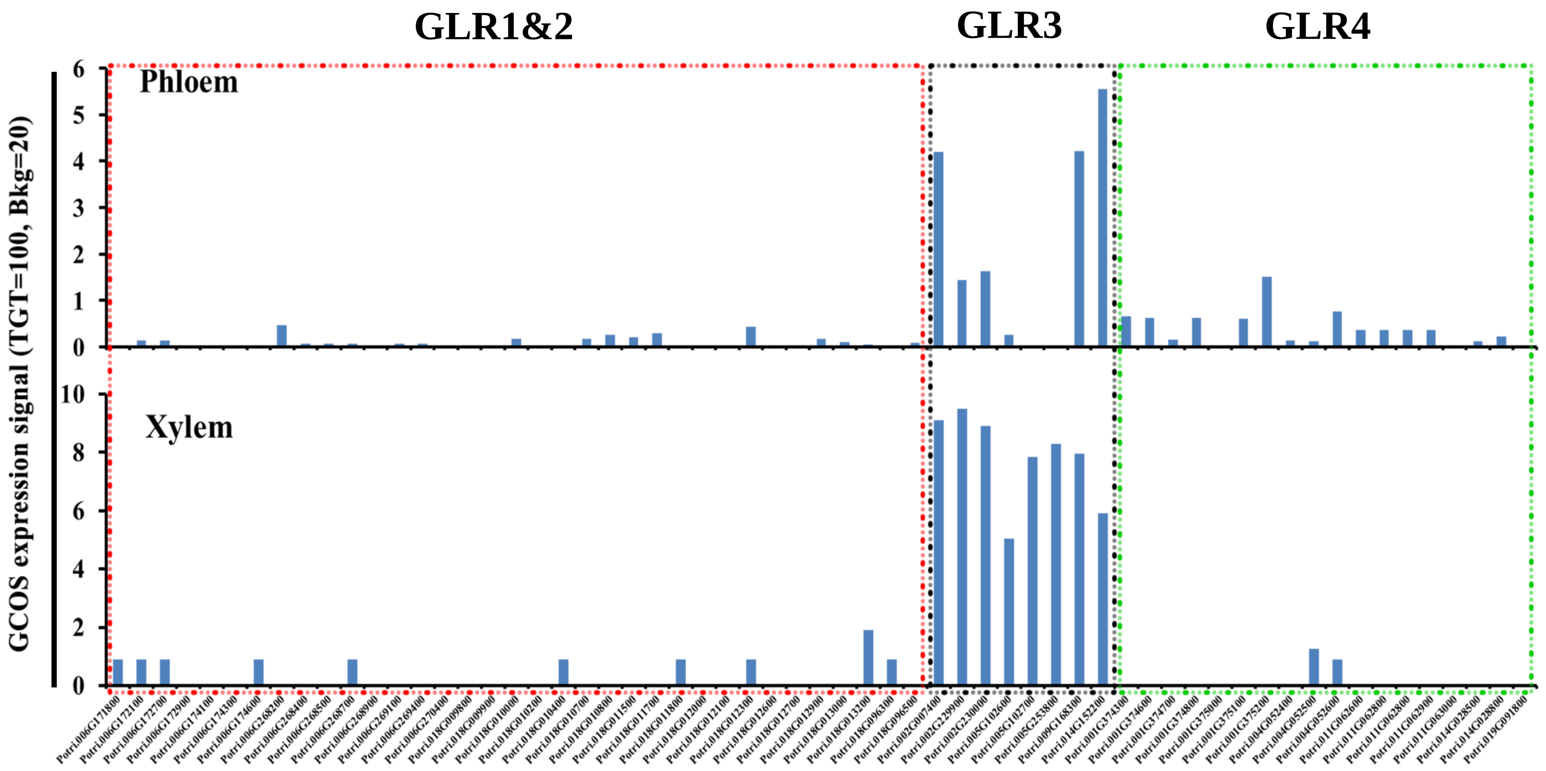

Fig. S8 Expression patterns of the Poplar GLR gene family during vascular tissue system.

Expression pattern of GLR genes in two vascular tissues, phloem and xylem, of Poplar**.** The different color of dotted boxes were represented three subfamily of GLRs. Expression data for selected genes for phloem and xylem were retrieved from The Populus Genome Integrative Explorer database (http://popgenie.org/) and poplar eFP Browser (http://bar.utoronto.ca/efp/cgi-bin/efpWeb.cgi), respectively. ATH1 data are normalized by the GCOS method, TGT value of 100.

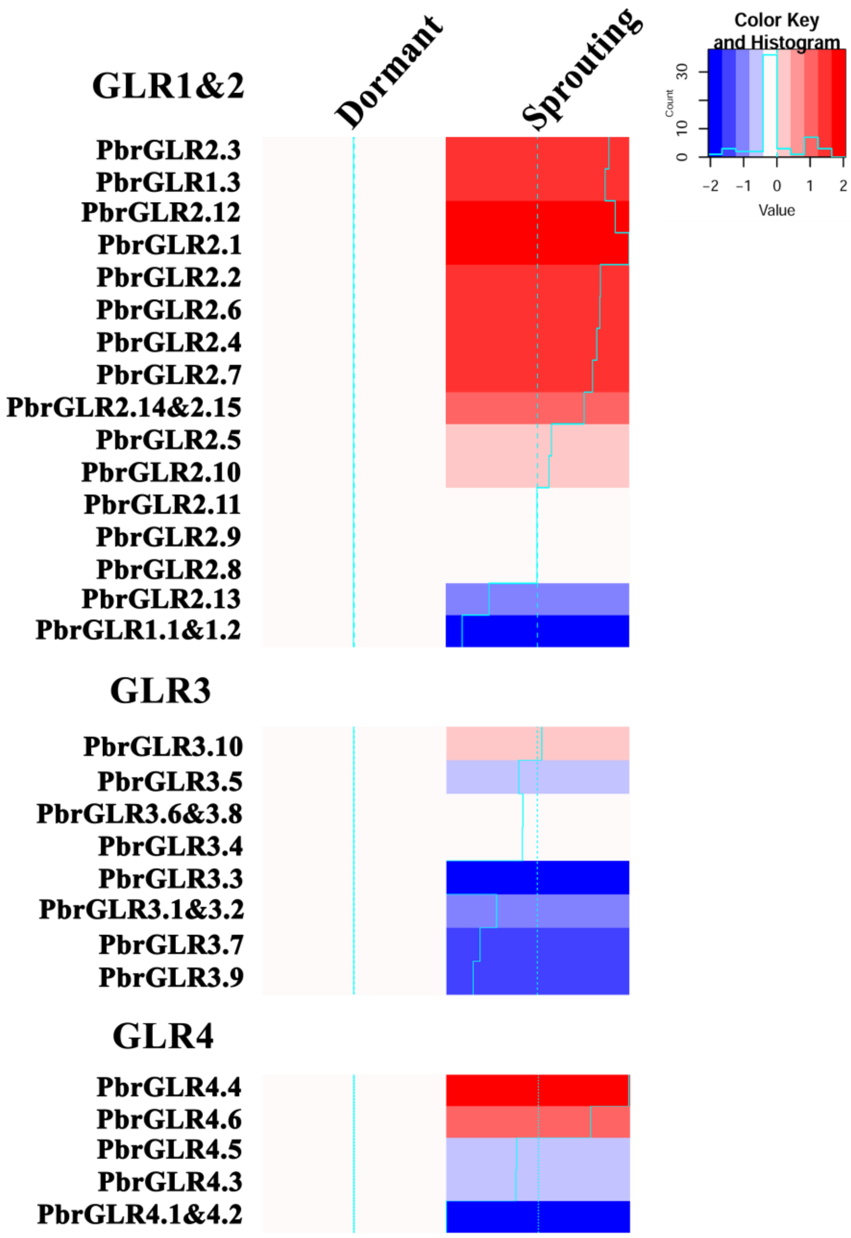

Fig. S9 Heat map of the qRT-PCR transcript abundance pattern of the 34 GLR genes from pear during sprouting of shoot tip.

Genes and samples were hierarchically clustered according to their transcript abundance (expressed in relation to the expression of each genes in dormant sample and log2-transformed). For each gene, its name is shown to the right of the heatmap. *PbrGLR2.8*, *PbrGLR2.9* and *PbrGLR2.11* could not be detected by RT-qPCR in the samples, because their expression levels were too low to detect by normal RT-PCR. There are 5 pair genes can't be distinguish because of high identify in sequences. An amplified *PbrACT and PbrCYP* were used as internal control.

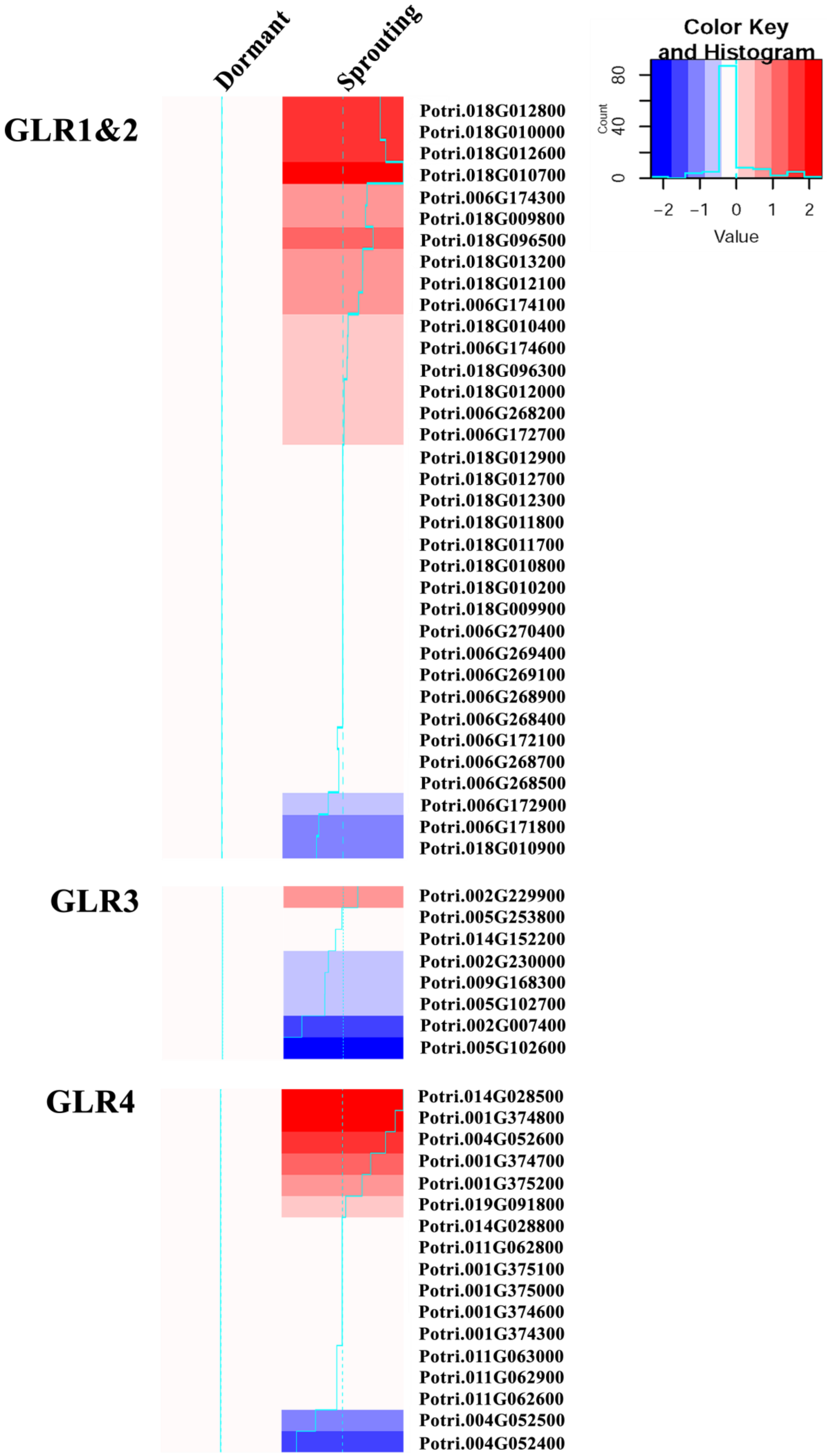
Dormant
Sprouting
Color Key and Histogram
Count
Value
GLR1&2
Potri.018G012800
Potri.018G010000
Potri.018G012600
Potri.018G010700
Potri.006G174300
Potri.018G009800
Potri.018G096500
Potri.018G013200
Potri.018G012100
Potri.006G174100
Potri.018G010400
Potri.006G174600
Potri.018G096300
Potri.018G012000
Potri.006G268200
Potri.006G172700
Potri.018G012900
Potri.018G012700
Potri.018G012300
Potri.018G011800
Potri.018G011700
Potri.018G010800
Potri.018G010200
Potri.018G009900
Potri.006G270400
Potri.006G269400
Potri.006G269100
Potri.006G268900
Potri.006G268400
Potri.006G172100
Potri.006G268700
Potri.006G268500
Potri.006G172900
Potri.006G171800
Potri.018G010900
GLR3
Potri.002G229900
Potri.005G253800
Potri.014G152200
Potri.002G230000
Potri.009G168300
Potri.005G102700
Potri.002G007400
Potri.005G102600
GLR4
Potri.014G028500
Potri.001G374800
Potri.004G052600
Potri.001G374700
Potri.001G375200
Potri.019G091800
Potri.014G028800
Potri.011G062800
Potri.001G375100
Potri.001G375000
Potri.001G374600
Potri.001G374300
Potri.011G063000
Potri.011G062900
Potri.011G062600
Potri.004G052500
Potri.004G052400

Fig. S10 Heat map of the RNAseq transcript abundance pattern of the 60 GLR genes from poplar during bud sprouting.

Genes and samples were hierarchically clustered according to their transcript abundance (expressed in relation to the expression of each genes in buds dormant sample and log2-transformed). For each gene, its name is shown to the right of the heatmap.

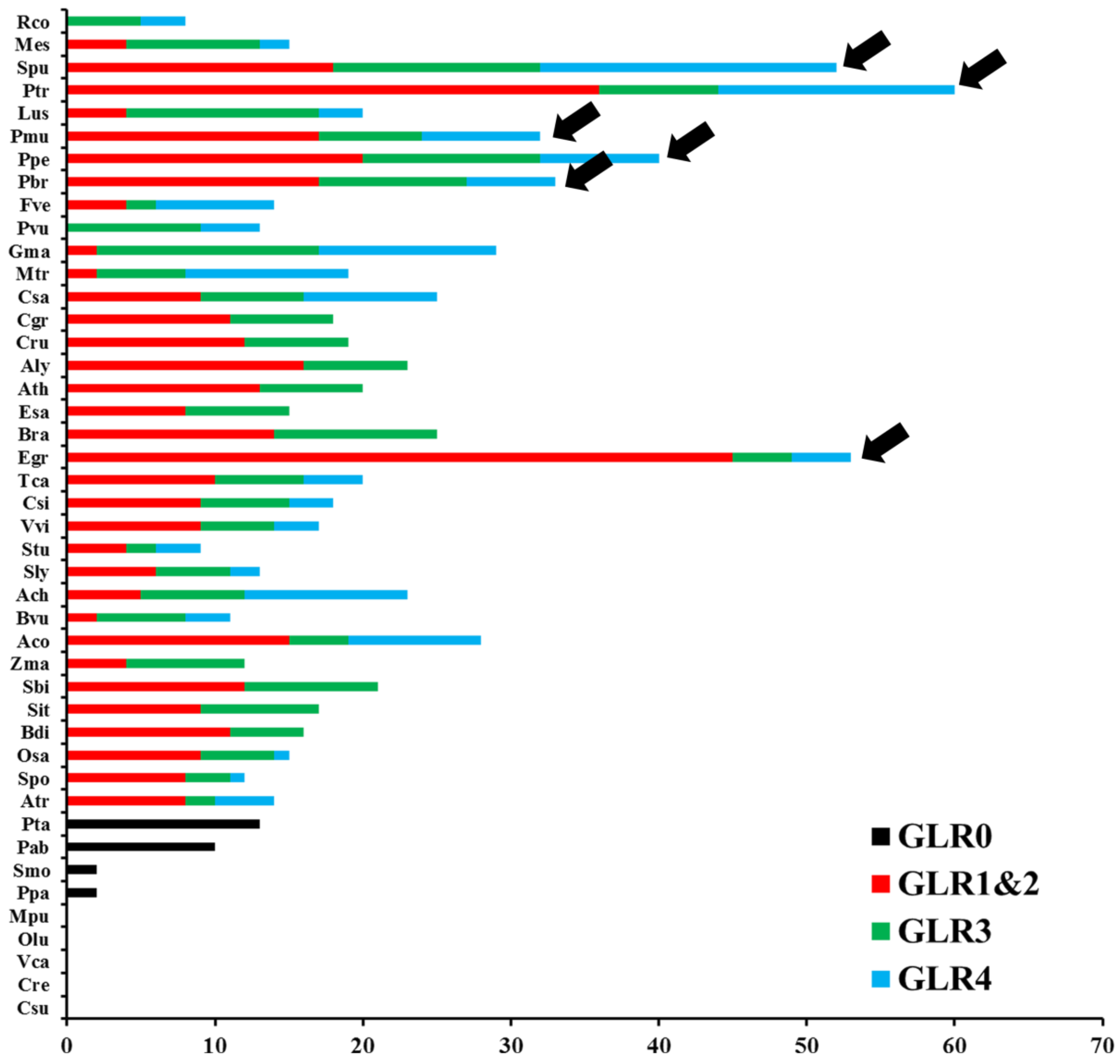

Fig. S11 Histogram of number of plant GLR genes in subfamily exclude lack ATD genes. Different color represent different GLR subfamilies. X axis indicated the number of genes. The black arrow indicated the highwood species.

Table S1. Identification of *GLR* genes in four Roseceae speices

Gene name	Gene_ID	Chromosome	Start	End	Strand	CDs length (bp)	Protein length(aa)	Exon number	Intron number
PbrGLR1.1	Pbr040724.1	Chr7	3592924	3595789	-	2226	742	4	3
PbrGLR1.2	Pbr039474.1	scaffold846.0	148401	151266	+	2226	742	4	3
PbrGLR1.3	Pbr033522.1	Chr2	3111137	3114503	-	2553	851	5	4
PbrGLR2.1	Pbr019949.1	Chr15	5773503	5776893	+	2871	957	5	4
PbrGLR2.2	Pbr036458.1	Chr8	16628537	16631927	+	2826	942	5	4
PbrGLR2.3	Pbr036459.1	Chr8	16617019	16620040	+	2595	865	4	3
PbrGLR2.4	Pbr019948.1	Chr15	5781572	5786726	+	2853	951	5	4
PbrGLR2.5	Pbr036457.1	Chr8	16634772	16637144	-	2058	686	3	2
PbrGLR2.6	Pbr036455.1	Chr8	16665631	16671765	+	2976	992	5	4
PbrGLR2.7	Pbr019946.4	Chr15	5813182	5816916	+	2916	972	5	4
PbrGLR2.8	Pbr036451.1	Chr8	16697153	16700171	+	2331	777	7	6
PbrGLR2.9	Pbr036450.1	Chr8	16708199	16711991	+	2736	912	6	5
PbrGLR2.10	Pbr038727.1	Chr14	20217054	20222367	-	2670	890	5	4
PbrGLR2.11	Pbr019841.2	Chr15	6539896	6542261	-	1947	649	4	3
PbrGLR2.12	Pbr019845.1	Chr15	6494988	6501622	-	2256	752	8	7
PbrGLR2.13	Pbr019842.1	Chr15	6528383	6532899	-	2838	946	6	5
PbrGLR2.14	Pbr000481.1	Chr5	25130433	25134565	+	2916	972	5	4
PbrGLR2.15	Pbr019843.1	Chr15	6513470	6517783	-	2937	979	5	4
PbrGLR3.1	Pbr023968.1	Chr5	15203742	15207372	-	2829	943	6	5
PbrGLR3.2	Pbr033289.1	Chr5	14364503	14368135	+	2829	943	6	5
PbrGLR3.3	Pbr015081.1	Chr6	20226225	20230590	+	2811	937	6	5
PbrGLR3.4	Pbr018096.3	Chr9	16476646	16480393	+	2880	960	6	5
PbrGLR3.5	Pbr018094.1	Chr9	16510920	16514801	+	2763	921	6	5
PbrGLR3.6	Pbr037336.1	Chr15	17534647	17538459	-	2802	934	6	5
PbrGLR3.7	Pbr018095.1	Chr9	16495385	16499659	+	2733	911	6	5
PbrGLR3.8	Pbr037340.1	Chr15	17491578	17497832	-	2778	926	6	5
PbrGLR3.9	Pbr037341.1	Chr15	17484242	17487667	-	2796	932	6	5
PbrGLR3.10	Pbr011753.1	Chr2	17205137	17209213	+	2904	968	7	6
PbrGLR4.1	Pbr038248.1	Chr11	4612910	4616377	+	2619	873	5	4
PbrGLR4.2	Pbr038245.1	Chr11	4555030	4558851	+	2619	873	5	4
PbrGLR4.3	Pbr026738.1	Chr3	2941735	2951990	+	2925	975	7	6
PbrGLR4.4	Pbr019641.1	Chr15	7925800	7928821	+	2583	861	5	4
PbrGLR4.5	Pbr000443.1	Chr5	25361347	25364742	+	2736	912	5	4
PbrGLR4.6	Pbr000439.1	Chr5	25383902	25387132	-	2487	829	6	5
FveGLR1.1	mrna07476.1-v1.0-hybrid	LG7	9760915	9765112	+	2706	902	6	5
FveGLR1.2	mrna07475.1-v1.0-hybrid	LG7	9751264	9758697	+	2610	870	8	7
FveGLR1.3	mrna07468.1-v1.0-hybrid	LG7	9700862	9706228	+	2541	847	6	5
FveGLR2.1	mrna25604.1-v1.0-hybrid	unanchored	6743044	6747915	+	2106	702	7	6
FveGLR2.2	mrna21273.1-v1.0-hybrid	LG7	19263916	19267164	-	1950	650	6	5
FveGLR2.3	mrna25608.1-v1.0-hybrid	unanchored	6784081	6787832	+	2109	703	5	4
FveGLR2.4	mrna25613.1-v1.0-hybrid	unanchored	6797735	6802147	+	2013	671	5	4
FveGLR2.5	mrna03026.1-v1.0-hybrid	LG3	9973934	9976266	+	1932	644	4	3
FveGLR2.6	mrna25614.1-v1.0-hybrid	unanchored	6805082	6808895	+	2091	697	5	4
FveGLR2.7	mrna26123.1-v1.0-hybrid	LG5	7936047	7939876	+	2211	737	6	5
FveGLR2.8	mrna17834.1-v1.0-hybrid	LG1	12584404	12588915	+	1881	627	6	5
FveGLR2.9	mrna08666.1-v1.0-hybrid	LG2	19603842	19608163	+	2151	717	5	4
FveGLR2.10	mrna08662.1-v1.0-hybrid	LG2	19584450	19588324	+	2148	716	5	4
FveGLR2.11	mrna02680.1-v1.0-hybrid	LG2	18302727	18308894	-	2910	970	10	9
FveGLR2.12	mrna02681.1-v1.0-hybrid	LG2	18311393	18315262	-	2154	718	5	4
FveGLR2.13	mrna03400.1-v1.0-hybrid	LG3	13253496	13255755	-	1800	600	5	4
FveGLR2.14	mrna03314.1-v1.0-hybrid	LG3	13352301	13357625	-	2148	716	5	4
FveGLR2.15	mrna03399.1-v1.0-hybrid	LG3	13241224	13246737	-	1929	643	5	4
FveGLR2.16	mrna25605.1-v1.0-hybrid	unanchored	6761333	6765627	-	1923	641	7	6
FveGLR2.17	mrna08674.1-v1.0-hybrid	LG2	19645465	19649859	+	2124	708	7	6
FveGLR2.18	mrna08673.1-v1.0-hybrid	LG2	19639891	19642270	+	1734	578	4	3
FveGLR2.19	mrna08675.1-v1.0-hybrid	LG2	19651318	19654380	+	1698	566	5	4
FveGLR2.20	mrna25615.1-v1.0-hybrid	unanchored	6813774	6816666	+	1722	574	4	3
FveGLR2.21	mrna08667.1-v1.0-hybrid	LG2	19609277	19612577	+	2028	676	6	5
FveGLR2.22	mrna08664.1-v1.0-hybrid	LG2	19594931	19598400	+	1857	619	7	6
FveGLR2.23	mrna08669.1-v1.0-hybrid	LG2	19615607	19617870	+	1761	587	4	3
FveGLR3.1	mrna17332.1-v1.0-hybrid	LG2	10138827	10142329	+	2631	877	5	4
FveGLR3.2	mrna31504.1-v1.0-hybrid	LG5	20200730	20205009	-	2775	925	6	5
FveGLR4.1	mrna00166.1-v1.0-hybrid	LG3	8887035	8891172	-	2712	904	6	5
FveGLR4.2	mrna09108.1-v1.0-hybrid	LG2	21175798	21179659	-	3057	1019	7	6
FveGLR4.3	mrna09106.1-v1.0-hybrid	LG2	21164308	21171903	-	3315	1105	11	10
FveGLR4.4	mrna28556.1-v1.0-hybrid	LG3	17241697	17244883	+	2613	871	5	4
FveGLR4.5	mrna29797.1-v1.0-hybrid	LG3	4781993	4785097	-	2700	900	5	4
FveGLR4.6	mrna10719.1-v1.0-hybrid	LG5	11580234	11585482	-	2730	910	5	4
FveGLR4.7	mrna28558.1-v1.0-hybrid	LG3	17247679	17250714	+	2535	845	5	4
FveGLR4.8	mrna29796.1-v1.0-hybrid	LG3	4777660	4780698	-	2481	827	5	4
PmuGLR1.1	Pm030928	scaffold654	55912	60593	+	2508	836	8	7
PmuGLR1.2	Pm017396	Pm5	10819527	10823576	+	2232	744	5	4
PmuGLR1.3	Pm017430	Pm5	11067622	11071763	-	2646	882	5	4
PmuGLR1.4	Pm031378	scaffold988	5327	8824	+	2340	780	6	5
PmuGLR1.5	Pm030925	scaffold654	32327	35352	+	2217	739	6	5
PmuGLR2.1	Pm005630	Pm2	12063516	12066899	-	2865	955	5	4
PmuGLR2.2	Pm005629	Pm2	12055346	12058775	-	2901	967	5	4
PmuGLR2.3	Pm005622	Pm2	12005728	12009173	+	2898	966	5	4
PmuGLR2.4	Pm005627	Pm2	12041476	12048301	-	2820	940	5	4
PmuGLR2.5	Pm005628	Pm2	12049275	12054231	-	2772	924	6	5
PmuGLR2.6	Pm005626	Pm2	12037185	12040362	-	2727	909	5	4
PmuGLR2.7	Pm031292	scaffold900	2181	6721	+	2139	713	4	3
PmuGLR2.8	Pm005625	Pm2	12032019	12035488	-	2847	949	5	4
PmuGLR2.9	Pm005621	Pm2	11991600	11995094	-	2832	944	5	4
PmuGLR2.10	Pm005624	Pm2	12018294	12031271	-	2751	917	12	11
PmuGLR2.11	Pm022455	Pm6	17701385	17704320	-	2595	865	4	3
PmuGLR2.12	Pm005497	Pm2	11345161	11348619	-	2808	936	5	4
PmuGLR2.13	Pm005495	Pm2	11339491	11342897	-	2766	922	5	4
PmuGLR2.14	Pm008541	Pm2	33868554	33871776	+	2745	915	5	4
PmuGLR3.1	Pm020680	Pm6	4376365	4382699	+	3834	1278	8	7
PmuGLR3.2	Pm022937	Pm7	567432	572001	-	2838	946	6	5
PmuGLR3.3	Pm015185	Pm4	17968624	17973025	+	2835	945	6	5
PmuGLR3.4	Pm026779	Pm8	11714206	11719541	-	3276	1092	11	10
PmuGLR3.5	Pm015184	Pm4	17957321	17961549	+	2757	919	6	5
PmuGLR3.6	Pm026778	Pm8	11706188	11710750	-	3384	1128	8	7
PmuGLR3.7	Pm031114	scaffold770	47377	50962	-	2172	724	8	7
PmuGLR4.1	Pm011362	Pm3	10575768	10578881	-	2550	850	5	4
PmuGLR4.2	Pm011356	Pm3	10543981	10550899	-	2457	819	5	4
PmuGLR4.3	Pm011354	Pm3	10534885	10538670	-	2634	878	6	5
PmuGLR4.4	Pm011358	Pm3	10557391	10560143	-	2424	808	4	3
PmuGLR4.5	Pm005285	Pm2	10194951	10198259	-	2679	893	6	5
PmuGLR4.6	Pm005286	Pm2	10199067	10202054	-	2538	846	5	4
PmuGLR4.7	Pm010115	Pm3	2629762	2632906	-	2751	917	5	4
PmuGLR4.8	Pm010111	Pm3	2610871	2614112	+	2817	939	5	4
PpeGLR1.1	ppa016169m	2	13604097	13608614	+	2652	884	6	5
PpeGLR1.2	ppa024348m	2	13429187	13432914	+	2487	829	5	4
PpeGLR1.3	ppa014655m	2	13570424	13572703	+	1965	655	3	2
PpeGLR1.4	ppa016897m	2	13437296	13442574	+	2565	855	7	6
PpeGLR1.5	ppa020480m	2	13534350	13537805	+	2391	797	8	7
PpeGLR1.6	ppa025893m	2	13504003	13507392	+	2562	854	5	4
PpeGLR2.1	ppa016908m	1	34336951	34340330	+	2808	936	6	5
PpeGLR2.2	ppa017927m	1	34347504	34352094	+	2739	913	6	5
PpeGLR2.3	ppa024352m	1	34384003	34386407	-	2040	680	4	3
PpeGLR2.4	ppa022588m	1	34358273	34362790	+	2820	940	5	4
PpeGLR2.5	ppa000839m	1	34353391	34357305	+	2958	986	5	4
PpeGLR2.6	ppa019874m	1	34363789	34367129	+	2847	949	6	5
PpeGLR2.7	ppa019950m	1	34368867	34373416	+	2796	932	6	5
PpeGLR2.8	ppa020965m	2	3032378	3035632	+	2046	682	8	7
PpeGLR2.9	ppa014659m	1	34410399	34413972	+	2913	971	5	4
PpeGLR2.10	ppa014704m	1	34378756	34381948	+	2433	811	7	6
PpeGLR2.11	ppa021995m	1	35019395	35023666	+	2739	913	7	6
PpeGLR2.12	ppa019144m	1	35013898	35017696	+	2817	939	6	5
PpeGLR2.13	ppa1027121m	1	6912799	6917648	+	2970	990	6	5
PpeGLR2.14	ppa026853m	1	6919329	6922474	+	2667	889	5	4
PpeGLR3.1	ppa021130m	8	17359034	17362481	+	2691	897	6	5
PpeGLR3.2	ppa001283m	5	868146	872617	-	2592	864	6	5
PpeGLR3.3	ppa001079m	3	16635990	16640329	+	2751	917	6	5
PpeGLR3.4	ppa001093m	3	16625236	16629452	+	2736	912	5	4
PpeGLR3.5	ppa022019m	7	18294528	18297811	+	2685	895	6	5
PpeGLR3.6	ppa025247m	7	18251688	18254895	+	2439	813	7	6
PpeGLR3.7	ppa014700m	7	18269482	18272497	+	2418	806	7	6
PpeGLR3.8	ppa020695m	7	18289005	18292724	+	2703	901	8	7
PpeGLR3.9	ppa023817m	7	18263599	18267114	+	2814	938	6	5
PpeGLR3.10	ppa001054m	7	13952451	13955971	+	2769	923	6	5
PpeGLR3.11	ppa001033m	7	20277270	20280749	-	2784	928	6	5
PpeGLR3.12	ppa000995m	7	18299631	18303237	+	2814	938	6	5
PpeGLR4.1	ppa017547m	4	10143585	10146697	+	2511	837	5	4
PpeGLR4.2	ppa024563m	4	10149650	10152813	+	2439	813	7	6
PpeGLR4.3	ppa001891m	4	10157769	10161693	+	2241	747	5	4
PpeGLR4.4	ppa022623m	4	10171960	10175565	+	2460	820	5	4
PpeGLR4.5	ppa020816m	1	36185954	36189006	+	2391	797	5	4
PpeGLR4.6	ppa017457m	1	36180957	36184991	+	2022	674	9	8
PpeGLR4.7	ppa001159m	4	2710639	2713781	-	2682	894	5	4
PpeGLR4.8	ppa021128m	4	2692319	2695924	+	2529	843	6	5

Table S2. The members of GLR family in plants

Orders/Families	Scientific name	Short name	Group 0	Group 1&2	Group 3	Group 4
Chlorophyta	Chlamydomonas reinhardtii	Cre	Cre13.g585400|Cre13.g585400.t2.1			
			Cre16.g685650|Cre16.g685650.t1.3			
			g13719|g13719.t2			
	Volvox carteri	Vca	Vocar20009035m			
			Vocar20009561m			
	Coccomyxa subellipsoidea C-169	Csu	62302			
Funariales	Physcomitrella patens	Ppa	Phpat.012G018400.1			
			Phpat.015G095400.1			
Selaginellales	Selaginella moellendorffii	Smo	92810			
			166047			
Pinales	Picea abies	Pab	MA_10426337g0010			
			MA_10427025g0010			
			MA_10430853g0010			
			MA_10434458g0010			
			MA_110963g0010			
			MA_141267g0010			
			MA_171056g0010			
			MA_196396g0010			
			MA_32301g0010			
			MA_354015g0010			
			MA_46902g0010			
			MA_88153g0010			
			MA_958834g0010			
	Pinus taeda	Pta	PITA_000049921			
			PITA_000013877			
			PITA_000061541			
			PITA_000012013			
			PITA_000037561			
			PITA_000058962			
			PITA_000030247			
			PITA_000056308			
			PITA_000023005			
			PITA_000040862			
			PITA_000011768			
			PITA_000020746			
			PITA_000031042			
Amborellales	Amborella trichopoda	Atr		evm_27.TU.AmTr_v1.0_scaffold00019.182	evm_27.TU.AmTr_v1.0_scaffold00062.28	evm_27.TU.AmTr_v1.0_scaffold00021.125
				evm_27.TU.AmTr_v1.0_scaffold00019.183	evm_27.TU.AmTr_v1.0_scaffold00044.128	evm_27.TU.AmTr_v1.0_scaffold00021.128
				evm_27.TU.AmTr_v1.0_scaffold00019.192		
				evm_27.TU.AmTr_v1.0_scaffold00019.197		evm_27.TU.AmTr_v1.0_scaffold00010.100
				evm_27.TU.AmTr_v1.0_scaffold00019.200		evm_27.TU.AmTr_v1.0_scaffold00010.102
				evm_27.TU.AmTr_v1.0_scaffold00019.204		
				evm_27.TU.AmTr_v1.0_scaffold00023.215		
				evm_27.TU.AmTr_v1.0_scaffold00055.178		
Alismatales	Spirodela Polyrhiza	Spo		Spipo12G0052500	Spipo14G0052100	Spipo26G0021700
				Spipo12G0052600	Spipo17G0033500	
				Spipo7G0047500	Spipo7G0016000	
				Spipo28G0017400		
				Spipo28G0017500		
				Spipo8G0005400		
				Spipo0G0098300		
				Spipo0G0098400		
Poales	Oryza sativa	Osa		LOC_Os09g25960.1	LOC_Os06g46670.2	LOC_Os06g06130.1
				LOC_Os09g25980.1	LOC_Os07g01310.1	
				LOC_Os09g25990.1	LOC_Os07g33790.1	
				LOC_Os09g26000.1	LOC_Os04g49570.2	
				LOC_Os09g26144.1	LOC_Os02g02540.1	
				LOC_Os09g26160.1		
				LOC_Os02g54640.1		
				LOC_Os06g08890.1		
				LOC_Os06g08900.1		
				LOC_Os06g08930.1		
				LOC_Os06g09050.1		
				LOC_Os06g09090.1		
				LOC_Os06g09120.1		
				LOC_Os06g09130.1		
	Brachypodium distachyon	Bdi		Bradi1g46910.2	Bradi1g59600.1	
				Bradi1g46920.1	Bradi1g32800.1	
				Bradi1g46940.1	Bradi3g01620.1	
				Bradi1g46947.2	Bradi1g26030.1	
				Bradi3g51890.1	Bradi5g19560.1	
				Bradi4g30810.1		
				Bradi4g30820.1		
				Bradi4g30840.1		
				Bradi4g30850.1		
				Bradi4g30860.1		
				Bradi4g30880.1		
				Bradi3g53690.1		
				Bradi2g41790.1		
				Bradi1g31350.1		
	Setaria italica	Sit		Si028879m	Si005801m	
				Si028880m	Si005804m	
				Si031875m	Si005805m	
				Si032907m	Si009407m	
				Si033011m	Si016244m	
				Si033384m	Si020680m	
				Si019519m	Si028848m	
				Si019713m	Si006014m	
				Si016208m		
				Si008039m		
				Si008207m		
				Si008309m		
	Sorghum bicolor	Sbi		Sobic.002G208300.1	Sobic.002G002700.1	
				Sobic.002G208500.1	Sobic.002G002800.1	
				Sobic.002G208700.1	Sobic.002G321400.1	
				Sobic.002G208800.1	Sobic.010G231100.1	
				Sobic.002G208900.1	Sobic.010G231300.1	
				Sobic.002G209200.1	Sobic.010G231500.1	
				Sobic.002G209000.1	Sobic.006G193200.1	
				Sobic.002G209100.1	Sobic.004G013200.1	
				Sobic.004G118000.1	Sobic.004G013300.1	
				Sobic.010G068600.1		
				Sobic.010G068800.1		
				Sobic.010G068900.1		
				Sobic.004G324000.1		
				Sobic.004G324400.1		
	Zea mays	Zma		GRMZM2G428379	GRMZM2G020104	
				GRMZM2G139933	GRMZM2G165828	

<table>
<tr><th></th><th></th><th></th><th></th><th></th><th></th></tr>
<tr><td></td><td></td><td></td><td>GRMZM2G391487</td><td>GRMZM2G125495</td><td></td></tr>
<tr><td></td><td></td><td></td><td>GRMZM2G302673</td><td>GRMZM2G148807</td><td></td></tr>
<tr><td></td><td></td><td></td><td>GRMZM2G341499</td><td>GRMZM2G057459</td><td></td></tr>
<tr><td></td><td></td><td></td><td></td><td>GRMZM2G066489</td><td></td></tr>
<tr><td></td><td></td><td></td><td></td><td>GRMZM2G098301</td><td></td></tr>
<tr><td></td><td></td><td></td><td></td><td>GRMZM2G150337</td><td></td></tr>
<tr><td>Ranunculales (Stem eudicotyledons)</td><td>Aquilegia coerulea</td><td>Aco</td><td>Aquca_018_00018.1</td><td>Aquca_077_00053.1</td><td>Aquca_055_00121.1</td></tr>
<tr><td></td><td></td><td></td><td>Aquca_018_00019.1</td><td>Aquca_077_00054.1</td><td>Aquca_055_00120.1</td></tr>
<tr><td></td><td></td><td></td><td>Aquca_018_00022.1</td><td></td><td>Aquca_055_00119.1</td></tr>
<tr><td></td><td></td><td></td><td>Aquca_018_00023.1</td><td>Aquca_015_00319.1</td><td>Aquca_055_00118.1</td></tr>
<tr><td></td><td></td><td></td><td>Aquca_018_00024.1</td><td>Aquca_015_00318.1</td><td></td></tr>
<tr><td></td><td></td><td></td><td>Aquca_018_00026.1</td><td></td><td>Aquca_005_00117.1</td></tr>
<tr><td></td><td></td><td></td><td>Aquca_018_00027.1</td><td></td><td>Aquca_005_00116.1</td></tr>
<tr><td></td><td></td><td></td><td>Aquca_018_00031.1</td><td></td><td>Aquca_005_00118.1</td></tr>
<tr><td></td><td></td><td></td><td>Aquca_018_00032.1</td><td></td><td>Aquca_005_00113.1</td></tr>
<tr><td></td><td></td><td></td><td>Aquca_018_00033.1</td><td></td><td></td></tr>
<tr><td></td><td></td><td></td><td>Aquca_005_00092.1</td><td></td><td>Aquca_012_00035.1</td></tr>
<tr><td></td><td></td><td></td><td>Aquca_031_00069.1</td><td></td><td></td></tr>
<tr><td></td><td></td><td></td><td>Aquca_031_00071.1</td><td></td><td></td></tr>
<tr><td></td><td></td><td></td><td>Aquca_016_00015.1</td><td></td><td></td></tr>
<tr><td></td><td></td><td></td><td>Aquca_005_00233.1</td><td></td><td></td></tr>
<tr><td>Caryophyllales</td><td>Beta vulgaris</td><td>Bvu</td><td>Bv6_148780_pnnc.t1</td><td>Bv6_132650_aphq.t1</td><td>Bv8_191200_zmwh.t1</td></tr>
<tr><td></td><td></td><td></td><td>Bv6_148820_wmiy.t1</td><td>Bv6_132660_xasr.t1</td><td>Bv8_191220_uiui.t1</td></tr>
<tr><td></td><td></td><td></td><td>Bv5_101990_yewj.t1</td><td>Bv1_016700_iccf.t1</td><td>Bv1_005950_zdui.t1</td></tr>
<tr><td></td><td></td><td></td><td>Bv2u_047600_tarh.t1</td><td>Bv9_221740_pjxx.t1</td><td></td></tr>
<tr><td></td><td></td><td></td><td></td><td>Bv9_221750_xswf.t1</td><td></td></tr>
<tr><td></td><td></td><td></td><td></td><td>Bv9_213320_ctxw.t1</td><td></td></tr>
<tr><td>Asterids</td><td>Actinidia chinensis</td><td>Ach</td><td>Achn034951</td><td>Achn362491</td><td>Achn227401</td></tr>
<tr><td></td><td></td><td></td><td>Achn013741</td><td>Achn362701</td><td>Achn227411</td></tr>
<tr><td></td><td></td><td></td><td>Achn013861</td><td>Achn375281</td><td>Achn227431</td></tr>
<tr><td></td><td></td><td></td><td>Achn013881</td><td>Achn255411</td><td>Achn227441</td></tr>
<tr><td></td><td></td><td></td><td>Achn013891</td><td>Achn255761</td><td>Achn221651</td></tr>
<tr><td></td><td></td><td></td><td>Achn301241</td><td>Achn075951</td><td>Achn181031</td></tr>
<tr><td></td><td></td><td></td><td>Achn282371</td><td>Achn019671</td><td>Achn181051</td></tr>
<tr><td></td><td></td><td></td><td>Achn282391</td><td>Achn272181</td><td>Achn344661</td></tr>
<tr><td></td><td></td><td></td><td></td><td>Achn255431</td><td>Achn044191</td></tr>
<tr><td></td><td></td><td></td><td></td><td></td><td>Achn095021</td></tr>
<tr><td></td><td></td><td></td><td></td><td></td><td>Achn343251</td></tr>
<tr><td></td><td>Solanum lycopersicum</td><td>Sly</td><td>Solyc06g063170.2.1</td><td>Solyc07g052390.2.1</td><td>Solyc02g077290.1.1</td></tr>
<tr><td></td><td></td><td></td><td>Solyc06g063180.1.1</td><td>Solyc07g052400.2.1</td><td>Solyc04g078860.2.1</td></tr>
<tr><td></td><td></td><td></td><td>Solyc06g063190.2.1</td><td>Solyc05g045650.2.1</td><td></td></tr>
<tr><td></td><td></td><td></td><td>Solyc06g063200.1.1</td><td>Solyc04g082610.2.1</td><td></td></tr>
<tr><td></td><td></td><td></td><td>Solyc06g063210.2.1</td><td>Solyc02g082480.2.1</td><td></td></tr>
<tr><td></td><td></td><td></td><td>Solyc08g006500.2.1</td><td></td><td></td></tr>
<tr><td></td><td>Solanum tuberosum</td><td>Stu</td><td>PGSC0003DMT400023744</td><td>PGSC0003DMT400013497</td><td>PGSC0003DMT400020569</td></tr>
<tr><td></td><td></td><td></td><td>PGSC0003DMT400023746</td><td>PGSC0003DMT400025675</td><td>PGSC0003DMT400020576</td></tr>
<tr><td></td><td></td><td></td><td>PGSC0003DMT400067658</td><td></td><td>PGSC0003DMT400027830</td></tr>
<tr><td></td><td></td><td></td><td>PGSC0003DMT400068189</td><td></td><td>PGSC0003DMT400059098</td></tr>
<tr><td>Vitales</td><td>Vitis vinifera</td><td>Vvi</td><td>GSVIVT01029195001</td><td>GSVIVT01030600001</td><td>GSVIVT01014244001</td></tr>
<tr><td></td><td></td><td></td><td>GSVIVT01029198001</td><td>GSVIVT01030602001</td><td>GSVIVT01014251001</td></tr>
<tr><td></td><td></td><td></td><td></td><td>GSVIVT01013400001</td><td>GSVIVT01021161001</td></tr>
<tr><td></td><td></td><td></td><td>GSVIVT01033127001</td><td>GSVIVT01022297001</td><td></td></tr>
<tr><td></td><td></td><td></td><td>GSVIVT01033129001</td><td>GSVIVT01023870001</td><td></td></tr>
<tr><td></td><td></td><td></td><td>GSVIVT01033133001</td><td></td><td></td></tr>
<tr><td></td><td></td><td></td><td>GSVIVT01033142001</td><td></td><td></td></tr>
<tr><td></td><td></td><td></td><td>GSVIVT01033150001</td><td></td><td></td></tr>
<tr><td></td><td></td><td></td><td>GSVIVT01033156001</td><td></td><td></td></tr>
<tr><td></td><td></td><td></td><td>GSVIVT01033160001</td><td></td><td></td></tr>
<tr><td>Sapindales</td><td>Citrus sinensis</td><td>Csi</td><td>orange1.1g041608m</td><td>orange1.1g003633m</td><td>orange1.1g002454m</td></tr>
<tr><td></td><td></td><td></td><td>orange1.1g047109m</td><td>orange1.1g002267m</td><td>orange1.1g003821m</td></tr>
<tr><td></td><td></td><td></td><td>orange1.1g044527m</td><td>orange1.1g002211m</td><td>orange1.1g043468m</td></tr>
<tr><td></td><td></td><td></td><td>orange1.1g037761m</td><td>orange1.1g002301m</td><td></td></tr>
<tr><td></td><td></td><td></td><td>orange1.1g040871m</td><td>orange1.1g002505m</td><td></td></tr>
<tr><td></td><td></td><td></td><td>orange1.1g005717m</td><td>orange1.1g002309m</td><td></td></tr>
<tr><td></td><td></td><td></td><td>orange1.1g036525m</td><td></td><td></td></tr>
<tr><td></td><td></td><td></td><td>orange1.1g003054m</td><td></td><td></td></tr>
<tr><td></td><td></td><td></td><td>orange1.1g037841m</td><td></td><td></td></tr>
<tr><td></td><td></td><td></td><td>orange1.1g043276m</td><td></td><td></td></tr>
<tr><td></td><td></td><td></td><td>orange1.1g002352m</td><td></td><td></td></tr>
<tr><td>Malvales</td><td>Theobroma cacao</td><td>Tca</td><td>Tcacao|Thecc1EG002626|Thecc1EG002626t1</td><td>Tcacao|Thecc1EG011335|Thecc1EG011335t1</td><td>Tcacao|Thecc1EG029763|Thecc1EG029763t1</td></tr>
<tr><td></td><td></td><td></td><td>Tcacao|Thecc1EG002627|Thecc1EG002627t1</td><td>Tcacao|Thecc1EG011336|Thecc1EG011336t1</td><td>Tcacao|Thecc1EG029764|Thecc1EG029764t1</td></tr>
<tr><td></td><td></td><td></td><td>Tcacao|Thecc1EG038866|Thecc1EG038866t1</td><td>Tcacao|Thecc1EG007573|Thecc1EG007573t1</td><td></td></tr>
<tr><td></td><td></td><td></td><td>Tcacao|Thecc1EG009437|Thecc1EG009437t1</td><td>Tcacao|Thecc1EG034421|Thecc1EG034421t3</td><td>Tcacao|Thecc1EG031691|Thecc1EG031691t1</td></tr>
<tr><td></td><td></td><td></td><td>Tcacao|Thecc1EG046331|Thecc1EG046331t1</td><td>Tcacao|Thecc1EG000045|Thecc1EG000045t1</td><td>Tcacao|Thecc1EG031700|Thecc1EG031700t1</td></tr>
<tr><td></td><td></td><td></td><td>Tcacao|Thecc1EG046345|Thecc1EG046345t1</td><td>Tcacao|Thecc1EG000047|Thecc1EG000047t1</td><td>Tcacao|Thecc1EG033827|Thecc1EG033827t1</td></tr>
<tr><td></td><td></td><td></td><td>Tcacao|Thecc1EG046900|Thecc1EG046900t1</td><td></td><td>Tcacao|Thecc1EG000615|Thecc1EG000615t1</td></tr>
<tr><td></td><td></td><td></td><td>Tcacao|Thecc1EG036848|Thecc1EG036848t1</td><td></td><td>Tcacao|Thecc1EG000616|Thecc1EG000616t1</td></tr>
<tr><td></td><td></td><td></td><td>Tcacao|Thecc1EG036849|Thecc1EG036849t1</td><td></td><td>Tcacao|Thecc1EG000617|Thecc1EG000617t1</td></tr>
<tr><td></td><td></td><td></td><td>Tcacao|Thecc1EG036850|Thecc1EG036850t1</td><td></td><td>Tcacao|Thecc1EG000618|Thecc1EG000618t1</td></tr>
<tr><td></td><td></td><td></td><td>Tcacao|Thecc1EG036851|Thecc1EG036851t1</td><td></td><td></td></tr>
<tr><td></td><td></td><td></td><td>Tcacao|Thecc1EG036854|Thecc1EG036854t1</td><td></td><td></td></tr>
<tr><td>Myrtales</td><td>Eucalyptus grandis</td><td>Egr</td><td>Eucgr.C00158.1</td><td>Eucgr.K00799.1</td><td>Eucgr.E02656.1</td></tr>
<tr><td></td><td></td><td></td><td>Eucgr.C00159.1</td><td>Eucgr.F03574.1</td><td>Eucgr.E02657.1</td></tr>
<tr><td></td><td></td><td></td><td>Eucgr.C00160.1</td><td>Eucgr.I01532.1</td><td>Eucgr.E02658.1</td></tr>
<tr><td></td><td></td><td></td><td>Eucgr.C00161.1</td><td>Eucgr.I02216.1</td><td>Eucgr.E02659.1</td></tr>
<tr><td></td><td></td><td></td><td>Eucgr.C00162.1</td><td></td><td>Eucgr.E02661.1</td></tr>
<tr><td></td><td></td><td></td><td>Eucgr.C00163.1</td><td></td><td></td></tr>
<tr><td></td><td></td><td></td><td>Eucgr.C00260.1</td><td></td><td></td></tr>
<tr><td></td><td></td><td></td><td>Eucgr.C00262.1</td><td></td><td></td></tr>
<tr><td></td><td></td><td></td><td>Eucgr.C00423.1</td><td></td><td></td></tr>
<tr><td></td><td></td><td></td><td>Eucgr.C00425.1</td><td></td><td></td></tr>
<tr><td></td><td></td><td></td><td>Eucgr.C01608.1</td><td></td><td></td></tr>
<tr><td></td><td></td><td></td><td>Eucgr.C01612.1</td><td></td><td></td></tr>
<tr><td></td><td></td><td></td><td>Eucgr.C01616.1</td><td></td><td></td></tr>
<tr><td></td><td></td><td></td><td>Eucgr.C01622.1</td><td></td><td></td></tr>
<tr><td></td><td></td><td></td><td>Eucgr.C01849.1</td><td></td><td></td></tr>
<tr><td></td><td></td><td></td><td>Eucgr.C01852.1</td><td></td><td></td></tr>
<tr><td></td><td></td><td></td><td>Eucgr.C01854.1</td><td></td><td></td></tr>
<tr><td></td><td></td><td></td><td>Eucgr.C01857.1</td><td></td><td></td></tr>
<tr><td></td><td></td><td></td><td>Eucgr.C01861.1</td><td></td><td></td></tr>
<tr><td></td><td></td><td></td><td>Eucgr.C01862.1</td><td></td><td></td></tr>
<tr><td></td><td></td><td></td><td>Eucgr.C01869.1</td><td></td><td></td></tr>
<tr><td></td><td></td><td></td><td>Eucgr.C01870.1</td><td></td><td></td></tr>
<tr><td></td><td></td><td></td><td>Eucgr.C01871.1</td><td></td><td></td></tr>
<tr><td></td><td></td><td></td><td>Eucgr.C01873.1</td><td></td><td></td></tr>
<tr><td></td><td></td><td></td><td>Eucgr.C01874.1</td><td></td><td></td></tr>
</table>

			Eucgr.C01908.1		
			Eucgr.C02002.1		
			Eucgr.C02003.1		
			Eucgr.C02004.1		
			Eucgr.C02006.1		
			Eucgr.C02433.1		
			Eucgr.C02434.1		
			Eucgr.L00722.1		
			Eucgr.L00724.1		
			Eucgr.L00725.1		
			Eucgr.L00726.1		
			Eucgr.L00861.1		
			Eucgr.L00903.1		
			Eucgr.L00908.1		
			Eucgr.L02191.1		
			Eucgr.L02214.1		
			Eucgr.L02423.1		
			Eucgr.L02424.1		
			Eucgr.L02629.1		
			Eucgr.L02750.1		
			Eucgr.L03144.1		
			Eucgr.L03706.1		
			Eucgr.J03149.1		
			Eucgr.J03152.1		
**Brassicales**	**Brassica rapa FPsc v1.3**	**Bra**	Brara.A03780.1	Brara.E01142.1	
			Brara.B00398.1	Brara.E01143.1	
			Brara.B03434.1	Brara.C01649.1	
			Brara.B03435.1	Brara.C01650.1	
				Brara.A00302.1	
			Brara.D01493.1	Brara.H00536.1	
			Brara.D01494.1	Brara.H01149.1	
			Brara.D01500.1	Brara.I03484.1	
				Brara.I05466.1	
			Brara.D01744.1	Brara.J00340.1	
			Brara.D01745.1		
			Brara.D01746.1		
			Brara.E01369.1	Brara.G00725.1	
			Brara.F03008.1		
			Brara.G03376.1		
			Brara.I00394.1		
			Brara.I00396.1		
			Brara.J02775.1		
			Brara.K00757.1		
			Brara.K01640.1		
	**Eutrema salsugineum**	**Esa**	Thhalv10000589m	Thhalv10003614m	
			Thhalv10003642m	Thhalv10006709m	
			Thhalv10012669m	Thhalv10010111m	
			Thhalv10016191m	Thhalv10016204m	
			Thhalv10016194m	Thhalv10016205m	
			Thhalv10020081m	Thhalv10022533m	
			Thhalv10024356m	Thhalv10024351m	
			TThhalv10016027m		
	**Arabidopsis thaliana**	**Ath**	AT5G48400.2(AtGLR1.2)	AT2G17260.1(AtGLR3.1)	
			AT5G48410.1(AtGLR1.3)	AT2G32390.3(AtGLR3.5)	
				AT2G32400.1(AtGLR3.7)	
			AT3G04110.1(AtGLR1.1)	AT4G35290.2(AtGLR3.2)	
			AT3G07520.1(AtGLR1.4)	AT1G42540.1(AtGLR3.3)	
			AT5G27100.1(AtGLR2.1)	AT1G05200.1(AtGLR3.4)	
			AT5G11210.1(AtGLR2.5)	AT3G51480.1(AtGLR3.6)	
			AT5G11180.1(AtGLR2.6)		
			AT4G31710.1(AtGLR2.4)		
			AT2G24720.1(AtGLR2.2)		
			AT2G24710.1(AtGLR2.3)		
			AT2G29120.1(AtGLR2.7)		
			AT2G29110.1(AtGLR2.8)		
			AT2G29100.1(AtGLR2.9)		
	**Arabidopsis lyrata v1.0**	**Aly**	357044	906549	
			494895	913525	
			949254	947434	
			477672	344999	
			315594	470513	
			484562	482168	
			893036	485499	
			325650	491120	
			491571		
			320279		
			344051		
			899853		
			899847		
			932546		
			903647		
			887883		
			887884		
			481829		
	**Capsella rubella**	**Cru**	Crubella|Carubv10003344m.g|Carubv10003344m	Crubella|Carubv10022588m.g|Carubv10022588m	
			Crubella|Carubv10015911m.g|Carubv10015911m	Crubella|Carubv10022600m.g|Carubv10022600m	
			Crubella|Carubv10016736m.g|Carubv10016736m	Crubella|Carubv10004138m.g|Carubv10004138m	
			Crubella|Carubv10022322m.g|Carubv10022322m	Crubella|Carubv10008205m.g|Carubv10008205m	
			Crubella|Carubv10022586m.g|Carubv10022586m	Crubella|Carubv10012075m.g|Carubv10012075m	
			Crubella|Carubv10022590m.g|Carubv10022590m	Crubella|Carubv10012924m.g|Carubv10012924m	
			Crubella|Carubv10022594m.g|Carubv10022594m	Crubella|Carubv10018389m.g|Carubv10018389m	
			Crubella|Carubv10022601m.g|Carubv10022601m		
			Crubella|Carubv10024663m.g|Carubv10024663m		
			Crubella|Carubv10025603m.g|Carubv10025603m		
			Crubella|Carubv10025869m.g|Carubv10025869m		
			Crubella|Carubv10025872m.g|Carubv10025872m		
	**Capsella grandiflora**	**Cgr**	Cagra.0133s0095|Cagra.0133s0095.1	Cagra.0352s0027|Cagra.0352s0027.1	
			Cagra.10702s0007|Cagra.10702s0007.1	Cagra.0352s0028|Cagra.0352s0028.1	
			Cagra.14254s0004|Cagra.14254s0004.1	Cagra.6119s0002|Cagra.6119s0002.1	
			Cagra.15158s0003|Cagra.15158s0003.1	Cagra.6420s0005|Cagra.6420s0005.1	
			Cagra.1731s0001|Cagra.1731s0001.1	Cagra.2350s0073.1	
			Cagra.1731s0002|Cagra.1731s0002.1	Cagra.1671s0141|Cagra.1671s0141.1	
			Cagra.3030s0001|Cagra.3030s0001.1	Cagra.0926s0110|Cagra.0926s0110.1	
			Cagra.3264s0011|Cagra.3264s0011.1		
			Cagra.3264s0012|Cagra.3264s0012.1		
			Cagra.3264s0013|Cagra.3264s0013.1		
			Cagra.9632s0001|Cagra.9632s0001.1		
**Cucurbitales**	**Cucumis sativus**	**Csa**	Cucsa.063680.1	Cucsa.044290.1	Cucsa.122760.1

			Cucsa.063790.1	Cucsa.072730.1	Cucsa.122770.1
			Cucsa.055890.1	Cucsa.072740.1	Cucsa.122780.1
			Cucsa.092130.1	Cucsa.072750.1	Cucsa.232820.1
			Cucsa.163040.1	Cucsa.099750.1	Cucsa.396080.1
			Cucsa.163050.1	Cucsa.148460.1	Cucsa.396090.1
			Cucsa.163060.1	Cucsa.272280.1	Cucsa.396100.1
			Cucsa.163070.1		Cucsa.396110.1
			Cucsa.231790.1		Cucsa.396120.1
			Cucsa.325220.1		
			Cucsa.341300.1		
**Fabales**	**Medicago truncatula**	**Mtr**	Medtr6g045087.1	Medtr8g073210.1	Medtr0018s0230.1
			Medtr3g105610.1	Medtr8g073490.1	Medtr0018s0240.1
			Medtr3g105595.1	Medtr5g024350.1	
				Medtr3g115910.1	Medtr5g059900.1
				Medtr2g088430.1	Medtr5g059920.1
				Medtr2g088450.1	Medtr5g060330.1
					Medtr2g015260.1
					Medtr2g015270.1
					Medtr2g015280.1
					Medtr2g015290.1
					Medtr2g015310.1
					Medtr4g087925.1
	**Glycine max**	**Gma**	Glyma.07G226400.1	Glyma.13G308400.1	Glyma.13G172100.1
			Glyma.07G226500.1	Glyma.13G308500.1	Glyma.07G203700.1
			Glyma.13G093300.1		Glyma.13G233000.1
			Glyma.17G067200.1	Glyma.12G194100.1	Glyma.13G233300.1
				Glyma.12G194200.1	Glyma.13G233400.1
				Glyma.12G099500.1	
				Glyma.06G305000.1	Glyma.16G061600.1
				Glyma.14G001100.1	Glyma.16G061700.1
				Glyma.02G311500.1	Glyma.16G061800.1
				Glyma.06G016000.1	
				Glyma.04G016000.1	Glyma.06G233700.1
				Glyma.09G197100.1	Glyma.06G233900.1
				Glyma.09G197200.1	Glyma.06G233600.1
				Glyma.09G197400.1	Glyma.13G272400.1
				Glyma.11G087100.1	Glyma.14G083200.1
				Glyma.01G157900.1	Glyma.17G241900.1
				Glyma.16G111700.1	
	**Phaseolus vulgaris**	**Pvu**	Phvul.003G149500.1	Phvul.003G270800.1	Phvul.001G022400.1
			Phvul.003G149600.1	Phvul.003G270900.1	Phvul.006G179300.1
			Phvul.003G149700.1	Phvul.002G117500.1	Phvul.008G150400.1
				Phvul.003G057300.1	Phvul.003G270600.1
				Phvul.009G006500.1	
				Phvul.008G292200.1	
				Phvul.005G114800.1	
				Phvul.005G114900.1	
				Phvul.011G104300.1	
**Rosales**	**Fragaria vesca**	**Fve**	mrna07476.1-v1.0-hybrid（FveGLR1.1）	mrna17332.1-v1.0-hybrid(FveGLR3.1)	mrna09108.1-v1.0-hybrid(FveGLR4.2)
			mrna07475.1-v1.0-hybrid（FveGLR1.2）	mrna31504.1-v1.0-hybrid(FveGLR3.2)	mrna09106.1-v1.0-hybrid(FveGLR4.3)
			mrna07468.1-v1.0-hybrid（FveGLR1.3）		
			mrna21273.1-v1.0-hybrid(FveGLR2.2)		mrna29797.1-v1.0-hybrid(FveGLR4.5)
			mrna02680.1-v1.0-hybrid（FveGLR2.11）		mrna29796.1-v1.0-hybrid(FveGLR4.8)
			mrna02681.1-v1.0-hybrid（FveGLR2.12）		
			mrna03314.1-v1.0-hybrid(FveGLR2.14)		mrna28556.1-v1.0-hybrid(FveGLR4.4)
			mrna08666.1-v1.0-hybrid(FveGLR2.9)		mrna28558.1-v1.0-hybrid（FveGLR4.7）
			mrna08662.1-v1.0-hybrid(FveGLR2.10)		mrna10719.1-v1.0-hybrid（FveGLR4.6）
			mrna08673.1-v1.0-hybrid(FveGLR2.17)		mrna00166.1-v1.0-hybrid(FveGLR4.1)
			mrna08675.1-v1.0-hybrid(FveGLR2.18)		
			mrna25615.1-v1.0-hybrid(FveGLR2.19)		
			mrna08664.1-v1.0-hybrid(FveGLR2.21)		
			mrna08664.1-v1.0-hybrid(FveGLR2.22)		
			mrna08669.1-v1.0-hybrid(FveGLR2.23)		
			mrna03026.1-v1.0-hybrid(FveGLR2.5)		
			mrna25604.1-v1.0-hybrid(FveGLR2.1)		
			mrna25608.1-v1.0-hybrid(FveGLR2.3)		
			mrna25613.1-v1.0-hybrid(FveGLR2.4)		
			mrna25614.1-v1.0-hybrid(FveGLR2.6)		
			mrna08674.1-v1.0-hybrid(FveGLR2.16)		
			mrna08667.1-v1.0-hybrid(FveGLR2.20)		
			mrna26123.1-v1.0-hybrid(FveGLR2.7)		
			mrna03400.1-v1.0-hybrid(FveGLR2.13)		
			mrna25605.1-v1.0-hybrid(FveGLR2.15)		
			mrna17834.1-v1.0-hybrid(FveGLR2.8)		
	**Pyrus bretchneideri**	**Pbr**	Pbr040724.1(PbrGLR1.1)	Pbr037336.1(PbrGLR3.6)	Pbr038248.1(PbrGLR4.1)
			Pbr039474.1(PbrGLR1.2)	Pbr037340.1(PbrGLR3.8)	Pbr038245.1(PbrGLR4.2)
			Pbr033522.1(PbrGLR1.3)	Pbr037341.1(PbrGLR3.9)	
			Pbr019949.1(PbrGLR2.1)		Pbr026738.1(PbrGLR4.3)
			Pbr019948.1(PbrGLR2.4)	Pbr018096.3(PbrGLR3.4)	Pbr019641.1(PbrGLR4.4)
			Pbr019946.4(PbrGLR2.7)	Pbr018094.1(PbrGLR3.5)	Pbr000439.1(PbrGLR4.6)
			Pbr038727.1(PbrGLR2.10)	Pbr018095.1(PbrGLR3.7)	Pbr000443.1(PbrGLR4.5)
			Pbr019841.2(PbrGLR2.11)	Pbr023968.1(PbrGLR3.1)	
			Pbr019845.1(PbrGLR2.12)	Pbr033289.1(PbrGLR3.2)	
			Pbr019842.1(PbrGLR2.13)	Pbr015081.1(PbrGLR3.3)	
			Pbr019843.1(PbrGLR2.15)	Pbr011753.1(PbrGLR3.10)	
			Pbr000481.1(PbrGLR2.14)		
			Pbr036458.1(PbrGLR2.2)		
			Pbr036459.1(PbrGLR2.3)		
			Pbr036457.1(PbrGLR2.5)		
			Pbr036455.1(PbrGLR2.6)		
			Pbr036451.1(PbrGLR2.8)		
			Pbr036450.1(PbrGLR2.9)		
	**Prunus persica**	**Ppe**	Ppa016169m(PpeGLR1.1)	Ppa001079m(PpeGLR3.3)	Ppa020816m(PpeGLR4.5)
			Ppa024348m(PpeGLR1.2)	Ppa001093m(PpeGLR3.4)	Ppa017457m(PpeGLR4.6)
			Ppa014655m(PpeGLR1.3)	Ppa021130m(PpeGLR3.1)	
			Ppa016897m(PpeGLR1.4)	Ppa001283m(PpeGLR3.2)	Ppa017547m(PpeGLR4.1)
			Ppa020480m(PpeGLR1.5)	Ppa022019m(PpeGLR3.5)	Ppa024563m(PpeGLR4.2)
			Ppa025893m(PpeGLR1.6)	Ppa025247m(PpeGLR3.6)	Ppa001891m(PpeGLR4.3)
				Ppa014700m(PpeGLR3.7)	Ppa022623m(PpeGLR4.4)
			Ppa016908m(PpeGLR2.1)	Ppa020695m(PpeGLR3.8)	
			Ppa017927m(PpeGLR2.2)	Ppa023817m(PpeGLR3.9)	Ppa001159m(PpeGLR4.7)
			Ppa024352m(PpeGLR2.3)	Ppa000995m(PpeGLR3.12)	Ppa021128m(PpeGLR4.8)
			Ppa022588m(PpeGLR2.4)	Ppa001054m(PpeGLR3.10)	
			Ppa000839m(PpeGLR2.5)	Ppa001033m(PpeGLR3.11)	
			Ppa019874m(PpeGLR2.6)		
			Ppa019950m(PpeGLR2.7)		
			Ppa014659m(PpeGLR2.9)		
			Ppa014704m(PpeGLR2.10)		
			Ppa020965m(PpeGLR2.8)		
			Ppa021995m(PpeGLR2.11)		
			Ppa019144m(PpeGLR2.12)		
			Ppa1027121m(PpeGLR2.13)		

			Ppa026853m(PpeGLR2.14)		
	**Prunus mume**	**Pmu**	Pm030928(PmuGLR1.1)	Pm020680(PmuGLR3.1)	Pm011362(PmuGLR4.1)
			Pm030925(PmuGLR1.5)	Pm022937(PmuGLR3.2)	Pm011356(PmuGLR4.2)
			Pm017396(PmuGLR1.2)	Pm015185(PmuGLR3.3)	Pm011354(PmuGLR4.3)
			Pm017430(PmuGLR1.3)	Pm015184(PmuGLR3.5)	Pm011358(PmuGLR4.4)
			Pm031378(PmuGLR1.4)	Pm031114(PmuGLR3.7)	
			Pm031292(PmuGLR2.7)	Pm026779(PmuGLR3.4)	Pm005285(PmuGLR4.5)
			Pm005630(PmuGLR2.1)	Pm026778(PmuGLR3.6)	Pm005286(PmuGLR4.6)
			Pm005629(PmuGLR2.2)		
			Pm005622(PmuGLR2.3)		Pm010115(PmuGLR4.7)
			Pm005627(PmuGLR2.4)		Pm010111(PmuGLR4.8)
			Pm005628(PmuGLR2.5)		
			Pm005626(PmuGLR2.6)		
			Pm005625(PmuGLR2.8)		
			Pm005621(PmuGLR2.9)		
			Pm005624(PmuGLR2.10)		
			Pm022455(PmuGLR2.11)		
			Pm005497(PmuGLR2.12)		
			Pm005495(PmuGLR2.13)		
			Pm008541(PmuGLR2.14)		
**Malpighiales**	**Linum usitatissimum**	**Luc**	Lus10020109	Lus10012245	Lus10005276
			Lus10026913	Lus10013837	Lus10013952
			Lus10026876	Lus10013838	Lus10013976
			Lus10003436	Lus10015118	Lus10038670
			Lus10026235	Lus10016031	
				Lus10026552	
				Lus10026553	
				Lus10027170	
				Lus10027171	
				Lus10031560	
				Lus10035980	
				Lus10039671	
				Lus10039672	
	**Populus trichocarpa**	**Ptr**	Potri.018G009800.1	Potri.014G152200.1	Potri.001G374300.1
			Potri.018G009900.1	Potri.002G229900.1	Potri.001G374600.1
			Potri.018G010000.1	Potri.002G230000.1	Potri.001G374700.1
			Potri.018G010200.1	Potri.009G168300.1	Potri.001G374800.1
			Potri.018G010400.1	Potri.005G253800.1	Potri.001G375000.1
			Potri.018G010700.1	Potri.002G007400.1	Potri.001G375100.1
			Potri.018G010800.1	Potri.005G102600.1	Potri.001G375200.1
			Potri.018G011500.1	Potri.005G102700.1	Potri.007G044000.1
			Potri.018G011700.1		Potri.004G052400.1
			Potri.018G011800.1		Potri.004G052500.1
			Potri.018G012000.1		Potri.004G052600.1
			Potri.018G012100.1		
			Potri.018G012300.1		Potri.011G062600.1
			Potri.018G012600.1		Potri.011G062800.1
			Potri.018G012700.1		Potri.011G062900.1
			Potri.018G012900.1		
			Potri.018G013000.1		Potri.014G028500.1
			Potri.018G013200.1		Potri.014G028800.1
			Potri.006G268200.1		
			Potri.006G268400.1		
			Potri.006G268500.1		
			Potri.006G268700.1		
			Potri.006G268900.1		
			Potri.006G269100.1		
			Potri.006G269400.1		
			Potri.006G270400.1		
			Potri.006G171800.1		
			Potri.006G172100.1		
			Potri.006G172700.1		
			Potri.006G172900.1		
			Potri.006G174100.1		
			Potri.006G174300.1		
			Potri.006G174600.1		
			Potri.019G091800.1		
			Potri.018G096300.1		
			Potri.018G096500.1		
	**Salix purpurea**	**Spu**	SapurV1A.0183s0100.1	SapurV1A.1981s0010.1	SapurV1A.0270s0160.1
			SapurV1A.0183s0110.1	SapurV1A.1981s0030.1	SapurV1A.0270s0170.1
			SapurV1A.1546s0010.1	SapurV1A.1981s0040.1	SapurV1A.0270s0180.1
			SapurV1A.2475s0030.1	SapurV1A.1610s0040.1	SapurV1A.4334s0010.1
			SapurV1A.2475s0040.1	SapurV1A.0016s0450.1	SapurV1A.0083s0440.1
			SapurV1A.2997s0010.1	SapurV1A.0605s0040.1	SapurV1A.0083s0460.1
			SapurV1A.1126s0010.1	SapurV1A.0266s0230.1	SapurV1A.0083s0470.1
			SapurV1A.1126s0020.1	SapurV1A.0374s0350.1	SapurV1A.0083s0480.1
			SapurV1A.1126s0030.1	SapurV1A.1212s0010.1	
			SapurV1A.1126s0040.1	SapurV1A.3387s0010.1	SapurV1A.0012s0950.1
			SapurV1A.1126s0050.1	SapurV1A.1440s0030.1	SapurV1A.0012s0960.1
			SapurV1A.0419s0340.1	SapurV1A.1440s0050.1	SapurV1A.0012s0970.1
			SapurV1A.0967s0060.1		
			SapurV1A.0967s0070.1	SapurV1A.0402s0030.1	SapurV1A.0878s0110.1
			SapurV1A.0184s0440.1	SapurV1A.0402s0040.1	SapurV1A.0878s0140.1
			SapurV1A.0314s0330.1		
			SapurV1A.0314s0340.1		SapurV1A.2399s0010.1
			SapurV1A.0314s0350.1		SapurV1A.2399s0020.1
			SapurV1A.3310s0010.1		SapurV1A.0474s0280.1
					SapurV1A.0623s0070.1
					SapurV1A.1568s0090.1
					SapurV1A.1977s0030.1
					SapurV1A.4169s0010.1
	**Manihot esculenta**	**Mes**	Mesculenta|cassava4.1_027954m.g|cassava4.1_027954	Mesculenta|cassava4.1_029502m.g|cassava4.1_029502m	Mesculenta|cassava4.1_008029m.g|cassava4.1_008029m
			Mesculenta|cassava4.1_032905m.g|cassava4.1_032905	Mesculenta|cassava4.1_030019m.g|cassava4.1_030019m	Mesculenta|cassava4.1_023716m.g|cassava4.1_023716m
			Mesculenta|cassava4.1_033384m.g|cassava4.1_033384	Mesculenta|cassava4.1_001914m.g|cassava4.1_001914m	Mesculenta|cassava4.1_026507m.g|cassava4.1_026507m
			Mesculenta|cassava4.1_027103m.g|cassava4.1_027103	Mesculenta|cassava4.1_026419m.g|cassava4.1_026419m	
				Mesculenta|cassava4.1_028827m.g|cassava4.1_028827m	
				Mesculenta|cassava4.1_031195m.g|cassava4.1_031195m	
				Mesculenta|cassava4.1_001235m.g|cassava4.1_001235m	
				Mesculenta|cassava4.1_001288m.g|cassava4.1_001288m	
				Mesculenta|cassava4.1_001290m.g|cassava4.1_001290m	
	**Ricinus communis**	**Rco**	29848.m004483	29600.m000545	30209.m001537
			29848.m004485	29600.m000546	30209.m001539
			29848.m004487	30100.m000785	29646.m001106
			29848.m004488	30170.m013795	29900.m001569
			29848.m004489	29830.m001466	30115.m001203
			30131.m007169		
			30131.m007170		

Note: The blue background in the table representative the tandem duplication string. The red font genes representative the receptor absence ATD region. Picea abies and Pinus taeda unable to detect duplicate model, because they have not assembled genome data. all sequence came from Phytozome data base (http://phytozome.jgi.doe.gov/pz/portal.html)

Table S3. The duplicate mode in Rosaceae GLRs

Subfamily	Duplicated Pair (Pear)	Duplicated model	Duplicated Pair (Strawberry)	Duplicated model	Duplicated Pair (Plum)	Duplicated model	Duplicated Pair (Peach)	Duplicated model
GLR1	PbrGLR1.1-PbrGLR1.2	Segmental	FveGLR1.1-FveGLR1.2	Tandem	PmuGLR1.1-PmuGLR1.5	Tandem	PpeGLR1.1-PpeGLR1.2-PpeGLR1.3-PpeGLR1.4-PpeGLR1.5-PpeGLR1.6	Tandem
	PbrGLR1.3-PbrGLR1.1	Segmental	FveGLR1.3-FveGLR1.1	Dispersed	PmuGLR1.2-PmuGLR1.3	Dispersed		
					PmuGLR1.3-PmuGLR1.5	Dispersed		
					PmuGLR1.4-PmuGLR1.5	Dispersed		
GLR2	PbrGLR2.1-PbrGLR2.4-PbrGLR2.7	Tandem	FveGLR2.1-FveGLR2.3-FveGLR2.4-FveGLR2.6-FveGLR2.16-FveGLR2.20	Tandem	PmuGLR2.1-PmuGLR2.2-PmuGLR2.3-PmuGLR4-PmuGLR2.5-PmuGLR2.6-PmuGLR2.8-PmuGLR2.9-PmuGLR2.10	Tandem	PpeGLR2.1-PpeGLR2.2-PpeGLR2.3-PpeGLR2.4-PpeGLR2.5-PpeGLR2.6-PpeGLR2.7-PpeGLR2.9-PpeGLR2.10	Tandem
	PbrGLR2.2-PbrGLR2.3-PbrGLR2.5-PbrGLR2.6-PbrGLR2.8-PbrGLR2.9	Tandem	FveGLR2.9-FveGLR2.10-FveGLR2.17-FveGLR2.18-FveGLR2.19-FveGLR2.21-FveGLR2.22-FveGLR2.23	Tandem	PmuGLR2.8-PmuGLR2.7	Dispersed	PpeGLR2.11-PpeGLR2.12	Tandem
	PbrGLR2.11-PbrGLR2.12-PbrGLR2.13-PbrGLR2.15	Tandem	FveGLR2.11-FveGLR2.12	Tandem	PmuGLR2.12-PmuGLR2.13	Tandem	PpeGLR2.13-PpeGLR2.14	Tandem
	PbrGLR2.2-PbrGLR2.1	Segmental	FveGLR2.15-FveGLR2.3	Tandem	PmuGLR2.9-PmuGLR2.12	Dispersed	PpeGLR2.7-PpeGLR2.8	Dispersed
	PbrGLR2.3-PbrGLR2.7	Segmental	FveGLR2.2-FveGLR2.1	Dispersed	PmuGLR2.11-PmuGLR2.13	Dispersed	PpeGLR2.9-PpeGLR2.11	Dispersed
	PbrGLR2.10-PbrGLR2.7	Dispersed	FveGLR2.5-FveGLR2.1	Dispersed				
	PbrGLR2.12-PbrGLR2.7	Dispersed	FveGLR2.23-FveGLR2.5	Dispersed				
	PbrGLR2.14-PbrGLR2.5	Dispersed	FveGLR2.9-FveGLR2.12	Dispersed				
			FveGLR2.14-FveGLR2.4	Dispersed				
			FveGLR2.13-FveGLR2.4	Dispersed				
GLR3	PbrGLR3.4-PbrGLR3.5-PbrGLR3.7	Tandem	mrna13028-FveGLR3.2	Segmental	PmuGLR3.4-PmuGLR3.6-Pm026785	Tandem	PpeGLR3.3-PpeGLR3.4	Tandem
	PbrGLR3.4-PbrGLR3.5-PbrGLR3.7	Tandem	FveGLR3.1-FveGLR3.2	Dispersed	PmuGLR3.3-PmuGLR3.5	Tandem	PpeGLR3.5-PpeGLR3.6-PpeGLR3.7-PpeGLR3.8-PpeGLR3.9-PpeGLR3.12	Tandem
	PbrGLR3.1-PbrGLR3.2	Segmental	FveGLR3.2-mrna23938	Dispersed	PmuGLR3.1-PmuGLR3.2	Dispersed	PpeGLR3.1-PpeGLR3.2	Dispersed
	PbrGLR3.3-PbrGLR3.9	Segmental			PmuGLR3.2-PmuGLR3.6	Dispersed	PpeGLR3.2-PpeGLR3.4	Dispersed
	PbrGLR3.9-PbrGLR3.10	Segmental			PmuGLR3.3-PmuGLR3.6	Dispersed	PpeGLR3.2-PpeGLR3.11	Dispersed
	PbrGLR3.3-PbrGLR3.2	Dispersed			PmuGLR3.7-PmuGLR3.6	Dispersed	PpeGLR3.12-PpeGLR3.11	Dispersed
	PbrGLR3.3-PbrGLR3.5	Dispersed						
GLR4	PbrGLR4.1-PbrGLR4.2	Tandem	FveGLR4.2-FveGLR4.3	Tandem	PmuGLR4.1-PmuGLR4.2-PmuGLR4.3-PmuGLR4.4	Tandem	PpeGLR4.1-PpeGLR4.2-PpeGLR4.3-PpeGLR4.4	Tandem
	PbrGLR4.5-PbrGLR4.6	Tandem	FveGLR4.5-FveGLR4.8	Tandem	PmuGLR4.5-PmuGLR4.6	Tandem	PpeGLR4.5-PpeGLR4.6	Tandem
	PbrGLR4.3-PbrGLR4.2	Segmental	FveGLR4.4-FveGLR4.7	Tandem	PmuGLR4.7-PmuGLR4.8	Tandem	PpeGLR4.7-PpeGLR4.8	Tandem
	PbrGLR4.2-PbrGLR4.5	Segmental	FveGLR4.1-FveGLR4.2	Segmental	PmuGLR4.2-PmuGLR4.7	Segmental	PpeGLR4.1-PpeGLR4.8	Segmental
	PbrGLR4.4-PbrGLR4.5	Dispersed	FveGLR4.4-FveGLR4.5	Segmental	PmuGLR4.2-PmuGLR4.5	Dispersed	PpeGLR4.3-PpeGLR4.5	Dispersed
			FveGLR4.7-FveGLR4.8	Segmental				
			FveGLR4.1-FveGLR4.2	Dispersed				

Table S4. GLRs amino acid sequence identities among four Rosaceae species.

yrus bretschneideri GLR	PbrGLR1.1	PbrGLR1.2	PbrGLR1.3	PbrGLR2.1	PbrGLR2.2	PbrGLR2.3	PbrGLR2.4	PbrGLR2.5	PbrGLR2.6	PbrGLR2.7	PbrGLR2.8	PbrGLR2.9	PbrGLR2.10	PbrGLR2.11	PbrGLR2.12	PbrGLR2.13	PbrGLR2.14	PbrGLR2.15	PbrGLR3.1	PbrGLR3.2	PbrGLR3.3	PbrGLR3.4	PbrGLR3.5	PbrGLR3.6	PbrGLR3.7	PbrGLR3.8	PbrGLR3.9	PbrGLR3.10	PbrGLR4.1	PbrGLR4.2	PbrGLR4.3	PbrGLR4.4	PbrGLR4.5	PbrGLR4.6
PbrGLR1.1	100	99.6	67.14	40.93	42.74	40.22	39.23	40.92	41.14	42.92	41.25	41.43	39.69	41.82	41.22	41.45	41.9	40.38	33.99	33.99	32.68	33.43	33.84	33.61	30.64	33.24	32.54	33.05	30.03	29.89	29.51	30.66	28.16	31.24
PbrGLR1.2	99.6	100	67	40.8	42.6	40.36	39.37	40.76	41.01	42.78	41.1	41.28	39.69	42.03	41.22	41.45	41.9	40.38	34.13	34.13	32.82	33.15	33.57	33.75	30.5	33.38	32.26	32.77	30.17	30.03	29.66	30.66	28.45	31.39
PbrGLR1.3	67.14	67	100	38.92	41.1	39.09	39.88	39.25	38.07	40.24	41.01	39.87	37.73	41.02	40.97	39.78	40.82	39.61	30.07	30.07	29.48	28.31	29.16	29.3	28.75	28.73	30.5	30.53	28.05	28.18	26.86	26.76	28.07	29.21
PbrGLR2.1	40.93	40.8	38.92	100	69.83	66.79	59.46	59.82	57.14	59.04	59.71	58.07	54.97	49.67	52.74	56.17	56.75	56.73	32.51	32.51	34.12	32.67	33.1	32.13	33.26	31.91	32.51	31.6	32.06	31.82	31.44	30.43	29.52	29.5
PbrGLR2.2	42.74	42.6	41.1	69.83	100	73.04	57.89	59.68	58.82	60.44	59.55	59.3	57.93	47.71	52.94	55.12	56.39	55.83	33.18	33.3	33.45	33.15	32.95	31.47	32.1	31.28	32.16	32.39	30.89	30.53	31.06	30.48	29.4	31.83
PbrGLR2.3	40.22	40.36	39.09	66.79	73.04	100	56.51	57.86	55.94	56.53	56.65	56.67	54.12	46.67	49.84	52.37	53.47	52.76	34.4	34.28	34.54	34.82	34.25	32.75	33.16	32.03	33.38	33.37	31.61	30.94	31.44	30.52	28.93	31.04
PbrGLR2.4	39.23	39.37	39.88	59.46	57.89	56.51	100	70.28	74.53	56.26	55.48	54.51	54.33	45.62	50.14	53.49	53.71	53.17	30.99	31.1	31.98	32.96	32.43	31.07	33.33	30.99	29.7	29.61	32.89	32.15	31.43	32.14	30.01	29.93
PbrGLR2.5	40.92	40.76	39.25	59.82	59.68	57.86	70.28	100	72.77	55.47	55.5	55.07	50.88	52.35	52.4	54.32	54.3	53.86	34.4	34.4	34.24	36.75	36.14	34.68	35.04	34.43	33.98	34.08	34.5	33.7	33.39	30.28	31.83	28.98
PbrGLR2.6	41.14	41.01	38.07	57.14	58.82	55.94	74.53	72.77	100	53.58	54.06	53.07	51.31	44.07	48.02	51.13	50.89	50.99	30.17	30.07	30.78	32.68	33.07	30.95	29.76	30.68	29.19	27.81	32.03	31.44	31.1	31.53	28.41	28.77
PbrGLR2.7	42.92	42.78	40.24	59.04	60.44	56.53	56.26	55.47	53.58	100	84.79	82.51	62.87	56.54	58.96	61.51	63.62	62.45	33.82	33.82	33.07	33.04	33.33	32.66	32.41	32.47	32.88	31.64	31.7	31.58	31	31.23	31.84	33.94
PbrGLR2.8	41.25	41.1	41.01	59.71	59.55	56.65	55.48	55.5	54.06	84.79	100	93.01	62.87	57.71	59.79	61.4	63.62	61.82	34.61	34.61	34.03	34.58	34.12	34.04	33.14	33.76	33.38	32.96	31.06	31.21	30.82	31.23	32.84	33.7
PbrGLR2.9	41.43	41.28	39.87	58.07	59.3	56.67	54.51	55.07	53.07	82.51	93.01	100	59.26	55.27	57.51	60.18	61.79	60.4	32.78	32.78	32.34	32.9	32.97	32.13	31.13	31.93	32.33	31.57	29.8	29.92	29.57	30.82	31.33	32.87
PbrGLR2.10	39.69	39.69	37.73	54.97	57.93	54.12	54.33	50.88	51.31	62.87	62.87	59.26	100	54.72	56.68	60.05	62.28	61.82	32.46	32.46	31.61	30.47	31.59	32.9	30.55	32.83	31.25	31.43	30.18	29.94	29.52	28.62	30.95	30.14
PbrGLR2.11	41.82	42.03	41.02	49.67	47.71	46.67	45.62	52.35	44.07	56.54	57.71	55.27	54.72	100	63.29	63.64	66.24	63.17	32.59	32.59	33.22	33.51	33.39	32.8	31.64	32.14	32.92	31.77	31.32	32.08	31.58	32.28	31.48	35.61
PbrGLR2.12	41.22	41.22	40.97	52.74	52.94	49.84	50.14	52.4	48.02	58.96	59.79	57.51	56.68	63.29	100	78.77	81.13	79.19	31.49	31.49	30.98	31.75	32.63	31.55	30.43	30.99	31.55	31.01	28.39	29.02	28.9	28.85	31.53	33.63
PbrGLR2.13	41.45	41.45	39.78	56.17	55.12	52.37	53.49	54.32	51.13	61.51	61.4	60.18	60.05	63.64	78.77	100	90.37	89.21	30.8	30.8	30.47	31.19	31.9	30.61	30.08	30.53	30.69	29.98	31.1	31.23	31.24	30.6	30.88	32.56
PbrGLR2.14	41.9	41.9	40.82	56.75	56.39	53.47	53.71	54.3	50.89	63.62	63.62	61.79	62.28	66.24	81.13	90.37	100	92.89	31.57	31.57	30.37	31.83	32.43	30.73	30.56	30.87	31.14	30.44	29.77	30.01	30.15	29.88	30.41	32.37
PbrGLR2.15	40.38	40.38	39.61	56.73	55.83	52.76	53.17	53.86	50.99	62.45	61.82	60.4	61.82	63.17	79.19	89.21	92.89	100	31.24	31.24	30.37	31.28	31.98	29.94	29.42	30.43	30.92	30.22	29.77	29.89	29.68	30.24	30.41	31.59
PbrGLR3.1	33.99	34.13	30.07	32.51	33.18	34.4	30.99	34.4	30.17	33.82	34.61	32.78	32.46	32.59	31.49	30.8	31.57	31.24	100	99.26	56.49	52.07	53.84	54.27	49.27	54.11	57.25	56.43	29.71	29.59	28.4	28.43	29.12	28.46
PbrGLR3.2	33.99	34.13	30.07	32.51	33.3	34.28	31.1	34.4	30.07	33.82	34.61	32.78	32.46	32.59	31.49	30.8	31.57	31.24	99.26	100	56.82	51.96	53.73	54.38	49.61	54.22	57.59	56.75	29.59	29.47	28.28	28.55	29.23	28.46
PbrGLR3.3	32.68	32.82	29.48	34.12	33.45	34.54	31.98	34.24	30.78	33.07	34.03	32.34	31.61	33.22	30.98	30.47	30.37	30.37	56.49	56.82	100	54.67	56.83	55.71	50.61	55.46	60.8	58.67	29.5	29.62	27.96	27.65	28.14	27.24
PbrGLR3.4	33.43	33.15	28.31	32.67	33.15	34.82	32.96	36.75	32.68	33.04	34.58	32.9	30.47	33.51	31.75	31.19	31.83	31.28	52.07	51.96	54.67	100	92.93	52.54	48.07	52.45	53.51	52.59	30.3	30.3	27.82	29.94	27.29	27.95
PbrGLR3.5	33.84	33.57	29.16	33.1	32.95	34.25	32.43	36.14	33.07	33.33	34.12	32.97	31.59	33.39	32.63	31.9	32.43	31.98	53.84	53.73	56.83	92.93	100	54.56	49.38	54.06	55.79	55.52	30.7	30.83	28.9	30.24	27.61	27.93
PbrGLR3.6	33.61	33.75	29.3	32.13	31.47	32.75	31.07	34.68	30.95	32.66	34.04	32.13	32.9	32.8	31.55	30.61	30.73	29.94	54.27	54.38	55.71	52.54	54.56	100	47.21	92.65	62.21	60.41	29.98	29.86	27.43	27.75	25.76	25.77
PbrGLR3.7	30.64	30.5	28.75	33.26	32.1	33.16	33.33	35.04	29.76	32.41	33.14	31.13	30.55	31.64	30.43	30.08	30.56	29.42	49.27	49.61	50.61	48.07	49.38	47.21	100	47.26	48.04	47.05	28.06	27.82	27.24	26.25	26.01	24.61
PbrGLR3.8	33.24	33.38	28.73	31.91	31.28	32.03	30.99	34.43	30.68	32.47	33.76	31.93	32.83	32.14	30.99	30.53	30.87	30.43	54.11	54.22	55.46	52.45	54.06	92.65	47.26	100	62.51	60.28	29.72	29.48	27.43	27.14	25.65	25.39
PbrGLR3.9	32.54	32.26	30.5	32.51	32.16	33.38	29.7	33.98	29.19	32.88	33.38	32.33	31.25	32.92	31.55	30.69	31.14	30.92	57.25	57.59	60.8	53.51	55.79	62.21	48.04	62.51	100	87.82	29.31	28.95	27.31	27.98	27.36	27.47
PbrGLR3.10	33.05	32.77	30.53	31.6	32.39	33.37	29.61	34.08	27.81	31.64	32.96	31.57	31.43	31.77	31.01	29.98	30.44	30.22	56.43	56.75	58.67	52.59	55.52	60.41	47.05	60.28	87.82	100	29.7	29.34	26.42	27.28	25.67	26.88
PbrGLR4.1	30.03	30.17	28.05	32.06	30.89	31.61	32.89	34.5	32.03	31.7	31.06	29.8	30.18	31.32	28.39	31.1	29.77	29.77	29.71	29.59	29.5	30.3	30.7	29.98	28.06	29.72	29.31	29.7	100	93.35	81.19	38.85	34.28	34.06
PbrGLR4.2	29.89	30.03	28.18	31.82	30.53	30.94	32.15	33.7	31.44	31.58	31.21	29.92	29.94	32.08	29.02	31.23	30.01	29.89	29.59	29.47	29.62	30.3	30.83	29.86	27.82	29.48	28.95	29.34	93.35	100	82	39.33	34.16	33.94
PbrGLR4.3	29.51	29.66	26.86	31.44	31.06	31.44	31.43	33.39	31.1	31	30.82	29.57	29.52	31.58	28.9	31.24	30.15	29.68	28.4	28.28	27.96	27.82	28.9	27.43	27.24	27.43	27.31	26.42	81.19	82	100	37.99	31.66	32.91
PbrGLR4.4	30.66	30.66	26.76	30.43	30.48	30.52	32.14	30.28	31.53	31.23	31.23	30.82	28.62	32.28	28.85	30.6	29.88	30.24	28.43	28.55	27.65	29.94	30.24	27.75	26.25	27.14	27.98	27.28	38.85	39.33	37.99	100	34.75	33.03
PbrGLR4.5	28.16	28.45	28.07	29.52	29.4	28.93	30.01	31.83	28.41	31.84	32.84	31.33	30.95	31.48	31.53	30.88	30.41	30.41	29.12	29.23	28.14	27.29	27.61	25.76	26.01	25.65	27.36	25.67	34.28	34.16	31.66	34.75	100	61.49
PbrGLR4.6	31.24	31.39	29.21	29.5	31.83	31.04	29.93	28.98	28.77	33.94	33.7	32.87	30.14	35.61	33.63	32.56	32.37	31.59	28.46	28.46	27.24	27.95	27.93	25.77	24.61	25.39	27.47	26.88	34.06	33.94	32.91	33.03	61.49	100

Fragaria vesca GLRs	FveGLR1.1	FveGLR1.2	FveGLR1.3	FveGLR2.1	FveGLR2.2	FveGLR2.3	FveGLR2.4	FveGLR2.5	FveGLR2.6	FveGLR2.7	FveGLR2.8	FveGLR2.9	FveGLR2.10	FveGLR2.11	FveGLR2.12	FveGLR2.13	FveGLR2.14	FveGLR2.15	FveGLR2.16	FveGLR2.17	FveGLR2.18	FveGLR2.19	FveGLR2.20	FveGLR2.21	FveGLR2.22	FveGLR2.23	FveGLR3.1	FveGLR3.2	FveGLR4.1	FveGLR4.2	FveGLR4.3	FveGLR4.4	FveGLR4.5	FveGLR4.6	FveGLR4.7	FveGLR4.8
FveGLR1.1	100	57.86	57.19	37.09	35.95	34.09	38.44	37.57	37.36	37.21	35.94	35.42	35.22	35.63	35.84	34.11	35.31	33.33	32.14	33.06	33.79	30.58	35.99	31.63	29.51	34.94	24.1	27.7	23	22.33	20.41	22.36	22.41	24.21	22.73	22.14
FveGLR1.2	57.86	100	53.2	35.53	35.73	33.87	35.65	35.35	36.04	34.06	32.98	33.44	33.12	35.11	34.76	32.06	33.59	30.7	31.03	32.3	30.43	32.94	38.33	30.39	26.45	31.52	21.48	25.1	21.61	21.66	19.49	20.44	21.39	22.32	21.56	20.57
FveGLR1.3	57.19	53.2	100	38.66	37.1	34.51	37.54	37.43	36.82	34.97	34.56	36.78	36.39	34.65	35.18	33.82	34.26	33.33	32.95	32.68	33.26	31.22	35.5	30.77	27.58	35.15	22.04	24.74	21.2	21.83	20.54	18.91	17.96	20.49	18.94	19.14
FveGLR2.1	37.09	35.53	38.66	100	69.43	55.27	52.01	50.32	52.38	46.89	49.23	50.3	49.03	49.47	47.54	44.56	49.25	44.58	43.78	41.38	44.66	45.13	54.06	44.67	37.7	48.08	28.84	32.01	28.45	28.16	24.61	27.43	26.91	27.48	28.72	26.78
FveGLR2.2	35.95	35.73	37.1	69.43	100	55.43	54.83	53.62	56.58	49.05	49.73	52.14	50.56	50.47	50.72	46.84	50.16	47.71	49.82	44.26	48.9	44.8	51.88	50.17	42.59	50.28	29.03	30.66	28.19	27.87	24.67	26.38	26.12	26.94	26.61	25.49
FveGLR2.3	34.09	33.87	34.51	55.27	55.43	100	49.92	47.2	48.54	44.74	43.46	48.59	47.39	46.41	46.69	43.22	43.27	40.03	46.23	41.33	44.32	41.72	47.79	45.66	38.5	44.32	29.04	28.21	25.93	25.43	24.79	22.76	23.52	24.14	23.36	24.43
FveGLR2.4	38.44	35.65	37.54	52.01	54.83	49.92	100	60.94	67.07	51.92	50.89	54.63	53.53	50.93	50.63	56.7	62.5	57.69	58.11	46.94	51.48	50	56.84	54.58	49.64	58.94	29.97	31.26	28.45	28.29	25.46	29.31	29.35	27.75	29.35	29.55
FveGLR2.5	37.57	35.35	37.43	50.32	53.62	47.2	60.94	100	63.17	48.72	47.41	50.91	49.09	46.57	47.6	53.2	57.26	49.64	54.74	44.83	48.59	48.97	57.83	53.42	45.35	61.41	31.36	33.86	30.55	29.32	26.37	27.92	28.01	28.19	28.88	29.1
FveGLR2.6	37.36	36.04	36.82	52.38	56.58	48.54	67.07	63.17	100	49.85	48.55	52.57	50.6	49.62	49.55	56.48	61.14	59.66	57.77	47.97	52.66	52.38	59.47	56.96	47.99	57.84	28.76	31.24	28.29	28.66	25.39	26.7	27.55	27.07	27	26.12
FveGLR2.7	37.21	34.06	34.97	46.89	49.05	44.74	51.92	48.72	49.85	100	66.83	64.72	63.58	56.13	56.01	43.27	46.96	42.97	41.77	38.16	39.72	39.42	47.01	41.19	37.37	46.45	29.79	30.23	27.26	29.15	24.35	28.24	29.23	28.35	29.01	27.5
FveGLR2.8	35.41	32.98	34.56	49.23	49.73	43.46	50.89	47.41	48.55	66.83	100	60.23	59.67	53.14	54.11	42.6	46.3	43.4	41.92	37.85	38.46	38.41	44.24	40.74	40.04	43.47	28.08	30.53	28.47	30.34	25.24	29.3	30.89	30.09	29.78	28.54
FveGLR2.9	35.42	33.44	36.78	50.3	52.14	48.59	54.63	50.91	52.57	64.72	60.23	100	93.97	58.37	59.47	46.42	48.45	44.83	44.37	39.42	44.14	42.48	48.58	45.26	45.56	49.21	28.69	31.7	29.23	28.62	22.64	28.41	29.23	28.97	29.15	27.84
FveGLR2.10	35.22	33.12	36.39	49.03	50.56	47.39	53.53	49.09	50.6	63.58	59.67	93.97	100	57.66	58.17	46.07	47.63	44.31	42.88	38.15	42.42	41.13	46.96	43.68	43.76	48.23	28.79	31.18	28.37	28.39	22.17	28.17	28.7	28.44	28.52	27.37
FveGLR2.11	35.63	35.11	34.65	49.47	50.47	46.41	50.93	46.57	49.62	56.13	53.14	58.37	57.66	100	69.14	44.64	45.44	43.41	42.88	38.58	43.14	40.68	49.47	43.51	38.21	44.77	23.72	24.14	22.7	21.1	17.32	24.22	25.72	23.36	23.55	22.38
FveGLR2.12	35.84	34.76	35.18	47.54	50.72	46.69	50.63	47.6	49.55	56.01	54.11	59.47	58.17	69.14	100	43.92	47.07	44.3	43.8	38.72	42.13	41.44	50.28	41.55	35.65	44.17	28.95	31.27	26.4	27.13	22.37	27.61	28.07	27.34	30.36	28.87
FveGLR2.13	34.11	32.06	33.82	44.56	46.84	43.22	56.7	53.2	56.48	43.27	42.6	46.42	46.07	44.64	43.92	100	60.8	76.11	51.63	40.78	44.57	44.44	50	47.39	40.12	53.9	26.87	30.04	29.26	28.57	24.85	29.18	29.57	29.25	29.36	28.73
FveGLR2.14	35.31	33.59	34.26	49.25	50.16	43.27	62.5	57.26	61.14	46.96	46.3	48.45	47.63	45.44	47.07	60.8	100	63.48	53.61	43.9	47.9	48.71	50.84	50.98	42.81	57.42	28.64	29.68	27.07	25.79	21.98	28.02	28.46	27.67	27.43	26.87
FveGLR2.15	33.33	30.7	33.33	44.58	47.71	40.03	57.69	49.64	59.66	42.97	43.4	44.83	44.31	43.41	44.3	76.11	63.48	100	51.97	40.43	45.73	47.49	49.68	48.22	40.71	52.57	23.75	27.68	31.25	27.6	22.65	28.78	29.63	27.82	30.52	29.67
FveGLR2.16	32.14	31.03	32.95	43.78	49.82	46.23	58.11	54.74	57.77	41.77	41.92	44.37	42.88	42.88	43.8	51.63	53.61	51.97	100	46.95	51.42	47.05	52.79	52.84	44.1	54.11	27.82	26.52	24.32	25.31	22.68	23.41	23.65	23.56	24.02	24.95
FveGLR2.17	33.06	32.3	32.68	41.38	44.26	41.33	46.94	44.83	47.97	38.16	37.85	39.42	38.15	38.58	38.72	40.78	43.9	40.43	46.95	100	62.35	48.2	51.89	48.41	39.7	46.62	26.26	27.19	24.06	23.51	21.88	21.62	22.27	22.78	22.01	22.3
FveGLR2.18	33.79	30.43	33.26	44.66	48.9	44.32	51.48	48.59	52.66	39.72	38.46	44.14	42.42	43.14	42.13	44.57	47.9	47.49	51.42	62.35	100	48.51	52.94	55.61	46.58	51.03	28.23	30.1	28.12	27.52	25.53	24.64	24.57	25.43	24.52	25.39
FveGLR2.19	30.58	32.94	31.22	45.13	44.8	41.72	50	48.97	52.38	39.42	38.41	42.48	41.13	40.68	41.44	44.44	48.71	49.68	47.05	48.2	48.51	100	51.31	48.76	37.47	50	23.59	24.72	23.09	23.03	22.27	23.66	22.44	20.55	20.47	20.69
FveGLR2.20	35.99	38.33	35.5	54.06	51.88	47.79	56.84	57.83	59.47	47.01	44.24	48.58	46.96	49.47	50.28	50	50.84	48.22	52.79	51.89	52.94	51.31	100	54.09	40.79	54.65	30.36	30.96	25.83	25.68	22.47	26.41	26.3	25.97	24.86	25.76
FveGLR2.21	31.63	30.39	30.77	44.67	50.17	45.66	54.58	53.42	56.96	41.19	40.74	45.26	43.68	43.51	41.55	47.39	50.98	40.71	52.84	48.41	55.61	48.76	54.09	100	75.86	55.74	25.88	26.86	25.25	25.57	24.87	21.97	22.26	21.36	23.06	23.35
FveGLR2.22	29.51	26.45	27.58	37.7	42.59	38.5	49.64	45.35	47.99	37.37	40.04	45.56	43.76	38.21	35.65	40.12	42.81	52.57	44.1	39.7	46.58	37.47	40.79	75.86	100	47.73	23.99	23.71	23.11	22.1	20.62	19.08	19.23	18.79	21	20
FveGLR2.23	34.94	31.52	35.15	48.08	50.28	44.32	58.94	61.41	57.84	46.45	43.47	49.21	48.23	44.77	44.17	53.9	57.42	52.57	54.11	46.62	51.03	50	54.65	55.74	47.73	100	27.92	30.66	29.9	28.81	26.98	28.45	28.52	28.65	27.87	28.96
FveGLR3.1	24.1	21.48	22.04	28.84	29.03	29.04	29.97	31.36	28.76	29.79	28.08	28.69	28.79	23.72	28.95	26.87	28.64	23.75	27.82	26.26	28.23	23.59	30.36	25.88	23.99	27.92	100	56.79	21.87	19.07	17.67	18.08	19.18	19.58	19.78	18.26
FveGLR3.2	27.7	25.1	24.74	32.01	30.66	28.21	31.26	33.86	31.24	30.23	30.53	31.7	31.18	24.14	31.27	30.04	29.68	27.68	26.52	27.19	30.1	24.72	30.96	26.86	23.71	30.66	56.79	100	22.8	20.24	17.7	20.48	21.03	20.53	20.4	19.62
FveGLR4.1	23	21.61	21.2	28.45	28.19	25.93	28.45	30.55	28.29	27.26	28.47	29.23	28.37	22.7	26.4	29.26	27.07	31.25	24.32	24.06	28.12	23.09	25.83	25.25	23.11	29.9	21.87	22.8	100	35.15	31.78	34.12	33.57	35.09	34.61	34.5
FveGLR4.2	22.33	21.66	21.83	28.16	27.87	25.43	28.29	29.32	28.66	29.15	30.34	28.62	28.39	21.1	27.13	28.57	25.79	27.6	25.31	23.51	27.52	23.03	25.68	25.57	22.1	28.81	19.07	20.24	35.15	100	73.91	34.48	33.96	35.55	36	34.8
FveGLR4.3	20.41	19.49	20.54	24.61	24.67	24.79	25.46	26.37	25.39	24.35	25.24	22.64	22.17	17.32	22.37	24.85	21.98	22.65	22.68	21.88	25.53	22.27	22.47	24.87	20.62	26.98	17.67	17.7	31.78	73.91	100	31.53	31.46	31.82	33.61	32.87
FveGLR4.4	22.36	20.44	18.91	27.43	26.38	22.76	29.31	27.92	26.7	28.24	29.3	28.41	28.17	24.22	27.61	29.18	28.02	28.78	23.41	21.62	24.64	23.66	26.41	21.97	19.08	28.45	18.08	20.48	34.12	34.48	31.53	100	86.97	59.48	63.97	61.42
FveGLR4.5	22.41	21.39	17.96	26.91	26.12	23.52	29.35	28.01	27.55	29.23	30.89	29.23	28.7	25.72	28.07	29.57	28.46	29.63	23.65	22.27	24.57	22.44	26.3	22.26	19.23	28.52	19.18	21.03	33.57	33.96	31.46	86.97	100	58.56	59.85	57.85
FveGLR4.6	24.21	22.32	20.49	27.48	26.94	24.14	27.75	28.19	27.07	28.35	30.09	28.97	28.44	23.36	27.34	29.25	27.67	27.82	23.56	22.78	25.43	20.55	25.97	21.36	18.79	28.65	19.58	20.53	35.09	35.55	31.82	59.48	58.56	100	60.63	57.02
FveGLR4.7	22.73	21.56	18.94	28.72	26.61	23.36	29.35	28.88	27	29.01	29.78	29.15	28.52	23.55	30.36	29.36	27.43	30.52	24.02	22.01	24.52	20.47	24.86	23.06	21	27.87	19.78	20.4	34.61	36	33.61	63.97	59.85	60.63	100	87.38
FveGLR4.8	22.14	20.57	19.14	26.78	25.49	24.43	29.55	29.1	26.12	27.5	28.54	27.84	27.37	22.38	28.87	28.73	26.87	29.67	24.95	22.3	25.39	20.69	25.76	23.35	20	28.96	18.26	19.62	34.5	34.8	32.87	61.42	57.85	57.02	87.38	100

Prunus mume GLRs	PmuGLR1.1	PmuGLR1.2	PmuGLR1.3	PmuGLR1.4	PmuGLR1.5	PmuGLR2.1	PmuGLR2.2	PmuGLR2.3	PmuGLR2.4	PmuGLR2.5	PmuGLR2.6	PmuGLR2.7	PmuGLR2.8	PmuGLR2.9	PmuGLR2.10	PmuGLR2.11	PmuGLR2.12	PmuGLR2.13	PmuGLR2.14	PmuGLR3.1	PmuGLR3.2	PmuGLR3.3	PmuGLR3.4	PmuGLR3.5	PmuGLR3.6	PmuGLR3.7	PmuGLR4.1	PmuGLR4.2	PmuGLR4.3	PmuGLR4.4	PmuGLR4.5	PmuGLR4.6	PmuGLR4.7	PmuGLR4.8
PmuGLR1.1	100	87.29	86.76	75.17	85	39.53	40.72	40.33	41.11	41.6	41.34	39.43	40.84	42.13	33.33	42.26	41.91	42.06	42.13	30.39	32.09	31.21	31.35	30.11	30.81	30	29.38	29.49	27.88	29.56	30.17	28.31	27.73	27.63
PmuGLR1.2	87.29	100	94.06	82.98	87.96	37.85	39.09	38.55	38.75	39.41	39.91	35.15	38.33	41.45	32.98	40.39	40.26	41.47	41.39	29.55	29.11	29.96	28.73	27.45	30.34	30.33	28.59	26.97	26.43	27.74	28.09	26.19	26.95	27.27
PmuGLR1.3	86.76	94.06	100	83.31	88.08	39.16	39.72	39.38	40.31	40.84	40.68	37.4	39.31	40.79	34.52	41.37	41.5	41.76	41.22	30.27	30.5	30.31	29.61	28.49	30.78	30.51	29.72	28.39	27.28	28.87	28.47	27.88	27.36	27.34
PmuGLR1.4	75.17	82.98	83.31	100	83.52	32.23	32.46	32.2	32.16	33.11	34.33	29.1	32.35	33.15	26.56	32.56	33.47	34.26	33.87	24.66	25	25.2	25.24	24.22	25.8	24.2	25.11	22.79	21.82	22.94	24.93	24.96	21.6	22.34
PmuGLR1.5	85	87.96	88.08	83.52	100	39.42	39.8	39.51	39.53	41.55	41.67	39.01	40.5	40.96	33.05	40.92	41.85	42.31	40.83	29.63	32	31.75	30.44	30.16	30.7	30.92	29.62	28.42	27.93	30.05	29.12	26.23	28.74	28.18
PmuGLR2.1	39.53	37.85	39.16	32.23	39.42	100	73.25	72.9	60.89	61.9	61.19	58.94	60.89	59.59	53.46	57.35	58.59	58.48	45.99	31.09	31.09	30.93	29.78	30.48	29.99	30.11	35.07	32.2	30.29	32.01	31.23	29.9	32.82	30.76
PmuGLR2.2	40.72	39.09	39.72	32.46	39.8	73.25	100	95.54	59.31	60.6	60.52	59.18	59.98	60.31	52.39	58.24	59.47	58.47	47.07	30.41	31.41	31.47	29.85	30.52	29.9	30.87	33.91	31.66	30.89	32.27	30.79	30.09	31.5	29.38
PmuGLR2.3	40.33	38.55	39.38	32.2	39.51	72.9	95.54	100	59.05	59.78	59.91	58.82	59.18	59.72	52.45	57.95	58.99	58.09	46.68	30.88	31.56	31.39	29.89	30.44	30.15	31.22	33.95	32.08	31.17	32.57	30.94	30.37	31.19	29.3
PmuGLR2.4	41.11	38.75	40.31	32.16	39.53	60.89	59.31	59.05	100	77.85	67.08	70.71	66.81	56.83	49.4	53.95	55.64	55.33	47.15	30.8	32.64	32.05	31.52	32.16	31.01	33.33	34.49	32.78	31.72	32.61	33.01	33.12	30.97	30.08
PmuGLR2.5	41.6	39.41	40.84	33.11	41.55	61.9	60.6	59.78	77.85	100	69.35	67.42	69.07	57.02	49.45	54.7	56.63	56.78	46.63	30.12	32	32.33	30.84	30.78	30.76	31.92	34.53	32	30.68	31.87	32.16	31.53	31.23	30.31
PmuGLR2.6	41.34	39.91	40.68	34.33	41.67	61.19	60.52	59.91	67.08	69.35	100	71.85	76	56.87	51.34	54.3	55.96	55.8	47.02	30.08	30.69	32.07	30.94	31.75	29.03	29.41	33.96	32.49	30.89	31.44	32.24	31.48	30.25	28.97
PmuGLR2.7	39.43	35.15	37.4	29.1	39.01	58.94	59.18	58.82	70.71	67.42	71.85	100	82.83	51.52	48.92	52.54	51.61	53.74	42.54	28.57	30.08	30.59	28.14	29.79	28.75	32.56	29.42	29.34	28.21	28.33	29.65	30.92	26.85	25.61
PmuGLR2.8	40.84	38.33	39.31	32.35	40.5	60.89	59.98	59.18	66.81	69.07	76	82.83	100	55.8	48.08	56.23	56.57	56.72	45.92	29.92	30.93	31.56	30.15	30.15	30.27	31.72	34.09	31.79	30.1	32.17	31.49	31.53	29.48	28.44
PmuGLR2.9	42.13	41.45	40.79	33.15	40.96	59.59	60.31	59.72	56.83	57.02	56.87	51.52	55.8	100	69.42	62.81	64.9	65.2	46.75	32.15	32.19	32.96	31.48	32.6	32.23	33.23	33.25	33.12	31.84	32.74	33.01	32.39	34.38	32.74
PmuGLR2.10	33.33	32.98	34.52	26.56	33.05	53.46	52.39	52.45	49.4	49.45	51.34	48.92	48.08	69.42	100	57.85	58.16	59.84	41.04	26.45	28.13	29.83	27.28	29.06	26.37	29.42	29.62	31.87	29.41	32	28.43	30.49	29.61	28.25
PmuGLR2.11	42.26	40.39	41.37	32.56	40.92	57.35	58.24	57.95	53.95	54.7	54.3	52.54	56.23	62.81	57.85	100	71.24	72.27	45.44	32.57	32.03	32.29	30.85	31.91	31.65	32.79	33.29	32.67	32.04	31.22	32.13	31.83	32.68	30.65
PmuGLR2.12	41.91	40.26	41.5	33.47	41.85	58.59	59.47	58.99	55.64	56.63	55.96	51.61	56.57	64.9	58.16	71.24	100	87.72	47.12	31.9	31.98	32.91	30.87	31.92	31.68	32.75	32.29	32.12	30.64	31.01	31.23	31.41	34.18	31.57
PmuGLR2.13	42.06	41.47	41.76	34.26	42.31	58.48	58.47	58.09	55.33	56.78	55.8	53.74	56.72	65.2	59.84	72.27	87.72	100	46.3	32.28	32.01	32.68	31.84	32.54	32.52	34.24	31.55	32.33	30.54	30.5	31.27	31.99	34.1	31.85
PmuGLR2.14	42.13	41.39	41.22	33.87	40.83	45.99	47.07	46.68	47.15	46.63	47.02	42.54	45.92	46.75	41.04	45.44	47.12	46.3	100	31.43	30.81	31.3	31.37	30.07	31.86	31.31	31.86	32.52	30.26	31.51	30.64	31.49	31.16	30.04
PmuGLR3.1	30.39	29.55	30.27	24.66	29.63	31.09	30.41	30.88	30.8	30.12	30.08	28.57	29.92	32.15	26.45	32.57	31.9	32.28	31.43	100	59.53	53.54	48.04	51.17	52.62	59.18	29.76	28.81	27.37	28.61	26.89	28.72	28.31	27.3
PmuGLR3.2	32.09	29.11	30.5	25	32	31.09	31.41	31.56	32.64	32	30.69	30.08	30.93	32.19	28.13	32.03	31.98	32.01	30.81	59.53	100	56.44	55.76	51.61	62.07	61.16	28.95	28.39	27.14	27.81	27.71	26.57	28.81	28.6
PmuGLR3.3	31.21	29.96	30.31	25.2	31.75	30.93	31.47	31.39	32.05	32.33	32.07	30.59	31.56	32.96	29.83	32.29	32.91	32.68	31.3	53.54	56.44	100	51.89	51.16	54.25	53.56	29.48	28.21	26.96	27.91	27.98	29.06	30.34	29.08
PmuGLR3.4	31.35	28.73	29.61	25.24	30.44	29.78	29.85	29.89	31.52	30.84	30.94	28.14	30.15	31.48	27.28	30.85	30.87	31.84	31.37	48.04	55.76	51.89	100	47.16	56.32	59.91	28.64	27.1	25.4	26.18	25.03	25.5	27.91	28.74
PmuGLR3.5	30.11	27.45	28.49	24.22	30.16	30.48	30.52	30.44	32.16	30.78	31.75	29.79	30.15	32.6	29.06	31.91	31.92	32.54	30.07	51.17	51.61	51.16	47.16	100	50.06	50.23	28.94	27.88	26.44	26.8	25.39	27.38	30.86	28.26
PmuGLR3.6	30.81	30.34	30.78	25.8	30.7	29.99	29.9	30.15	31.01	30.76	29.03	28.75	30.27	32.23	26.37	31.65	31.68	32.52	31.86	52.62	62.07	54.25	56.32	50.06	100	91.68	28.55	27.86	26.53	27.6	27.12	27.4	28.29	28.09
PmuGLR3.7	30	30.33	30.51	24.2	30.92	30.11	30.87	31.22	33.33	31.92	29.41	32.56	31.72	33.23	29.42	32.79	32.75	34.24	31.31	59.18	61.16	53.56	59.91	50.23	91.68	100	28.26	29.02	27.36	28.7	26.76	28.11	28.68	28.79
PmuGLR4.1	29.38	28.59	29.72	25.11	29.62	35.07	33.91	33.95	34.49	34.53	33.96	29.42	34.09	33.25	29.62	33.29	32.29	31.55	31.86	29.76	28.95	29.48	28.64	28.94	28.55	28.26	100	61.78	59.95	60.65	36.35	36.07	37.36	34.15
PmuGLR4.2	29.49	26.97	28.39	22.79	28.42	32.2	31.66	32.08	32.78	32	32.49	29.34	31.79	33.12	31.87	32.67	32.12	32.33	32.52	28.81	28.39	28.21	27.1	27.88	27.86	29.02	61.78	100	84.63	84.3	37.28	35.53	37.48	34.47
PmuGLR4.3	27.88	26.43	27.28	21.82	27.93	30.29	30.89	31.17	31.72	30.68	30.89	28.21	30.1	31.84	29.41	32.04	30.64	30.54	30.26	27.37	27.14	26.96	25.4	26.44	26.53	27.36	59.95	84.63	100	90.57	35.88	35.92	35.05	32.01
PmuGLR4.4	29.56	27.74	28.87	22.94	30.05	32.01	32.27	32.57	32.61	31.87	31.44	28.33	32.17	32.74	32	31.22	31.01	30.5	31.51	28.61	27.81	27.91	26.18	26.8	27.6	28.7	60.65	84.3	90.57	100	36.6	36.55	36.22	31.97
PmuGLR4.5	30.17	28.09	28.47	24.93	29.12	31.23	30.79	30.94	33.01	32.16	32.24	29.65	31.49	33.01	28.43	32.13	31.23	31.27	30.64	26.89	27.71	27.98	25.03	25.39	27.12	26.76	36.35	37.28	35.88	36.6	100	72.4	35.25	33.41
PmuGLR4.6	28.31	26.19	27.88	24.96	26.23	29.9	30.09	30.37	33.12	31.53	31.48	30.92	31.53	32.39	30.49	31.83	31.41	31.99	31.49	28.72	26.57	29.06	25.5	27.38	27.4	28.11	36.07	35.53	35.92	36.55	72.4	100	35.66	33.94
PmuGLR4.7	27.73	26.95	27.36	21.6	28.74	32.82	31.5	31.19	30.97	31.23	30.25	26.85	29.48	34.38	29.61	32.68	34.18	34.1	31.16	28.31	28.81	30.34	27.91	30.86	28.29	28.68	37.36	37.48	35.05	36.22	35.25	35.66	100	72.94
PmuGLR4.8	27.63	27.27	27.34	22.34	28.18	30.76	29.38	29.3	30.08	30.31	28.97	25.61	28.44	32.74	28.25	30.65	31.57	31.85	30.04	27.3	28.6	29.08	28.74	28.26	28.09	28.79	34.15	34.47	32.01	31.97	33.41	33.94	72.94	100

Prunus persica GLRs	PpeGLR1.1	PpeGLR1.2	PpeGLR1.3	PpeGLR1.4	PpeGLR1.5	PpeGLR1.6	PpeGLR2.1	PpeGLR2.2	PpeGLR2.3	PpeGLR2.4	PpeGLR2.5	PpeGLR2.6	PpeGLR2.7	PpeGLR2.8	PpeGLR2.9	PpeGLR2.10	PpeGLR2.11	PpeGLR2.12	PpeGLR2.13	PpeGLR2.14	PpeGLR3.1	PpeGLR3.2	PpeGLR3.3	PpeGLR3.4	PpeGLR3.5	PpeGLR3.6	PpeGLR3.7	PpeGLR3.8	PpeGLR3.9	PpeGLR3.10	PpeGLR3.11	PpeGLR3.12	PpeGLR4.1	PpeGLR4.2	PpeGLR4.3	PpeGLR4.4	PpeGLR4.5	PpeGLR4.6	PpeGLR4.7	PpeGLR4.8
PpeGLR1.1	100	69.35	65.63	65.24	55.14	64.17	39.58	39.15	41.33	40.48	40.11	39.15	40.57	36.63	41.07	39.61	40.21	40.87	37.7	39.56	30.04	31.33	30.53	28.74	30.69	27.44	30.86	29.27	30.75	30.39	31.07	30.75	32.9	28.72	31.17	30.74	26.93	24.04	29.04	26.95
PpeGLR1.2	69.35	100	68.77	67.64	59.38	66.5	38.9	39.93	42.98	40.17	41.1	41.12	40.78	36.39	41.69	40.43	41.79	42.03	39.22	40.53	30.8	31.33	29.54	29.84	30.62	27.2	30.62	29.16	30.08	29.68	30.66	30.08	31.4	28.36	30.28	29.36	26.26	24.36	30.76	28.31
PpeGLR1.3	65.63	68.77	100	91.1	90.78	89.06	41.11	42.41	44.38	41.33	42.41	42.06	42.41	39.53	42.92	40.88	43.3	42.06	42.63	43.75	33.98	33.92	34.39	30.32	33.97	30.19	32.94	32.32	33.6	33.39	33.76	33.28	31.64	28.72	30.84	31.06	26.43	23.96	29.2	27.31
PpeGLR1.4	65.24	67.64	91.1	100	79.43	88.34	39.6	40.78	42.83	40.78	40.98	41.07	41.38	36.97	40.78	40	41.58	40.83	41.18	41.96	31.44	31.73	31.93	28.72	31.88	29.35	31.33	31.59	31.5	31.53	31.88	31.5	31.41	28.43	29.99	29.73	26.95	23.88	29.42	28.05
PpeGLR1.5	55.14	59.38	90.78	79.43	100	77.21	34.74	36.79	42.67	35.16	35.34	35.59	36.07	37.43	35.99	37.13	37.29	36.06	36.59	37.69	30.76	32.19	30.61	27.92	30.77	27.9	30.39	29.61	30.09	29.5	30.45	30.09	27.66	24.72	28.48	26.74	23.8	23.19	24.42	23.42
PpeGLR1.6	64.17	66.5	89.06	88.34	77.21	100	37.17	38.33	41.71	39.66	39.29	39.44	40.59	36.11	40	39.86	40.07	39.76	40.1	41.06	30.95	32.25	31.46	28.36	31.09	28.26	30.64	29.71	30.45	30.19	30.9	30.45	30.55	27.39	29.45	29.18	25.48	22.74	26.92	26.1
PpeGLR2.1	39.58	38.9	41.11	39.6	34.74	37.17	100	72.04	69.53	60.86	60.6	59.82	63.5	60.84	59.42	59.51	59.08	57.66	45.55	47.05	33.26	33.33	31.13	29.77	31.23	28.54	32.36	31.34	30.49	30.15	30.52	30.16	34.11	29.8	33.1	31.79	28.16	24.54	30.89	28.37
PpeGLR2.2	39.15	39.93	42.41	40.78	36.79	38.33	72.04	100	95.53	60.73	60.44	61.1	63.85	60.54	61.07	62.23	58.61	59.1	46.9	49.53	33.61	34.95	33.56	30.63	31.34	28.14	32.74	31.93	31.58	31.26	32	31.46	33.62	29.84	33.29	31.95	27.81	25	30.4	27.76
PpeGLR2.3	41.33	42.98	44.38	42.83	42.67	41.71	69.53	95.53	100	57.38	58.26	57.51	59.76	60.4	58.52	56.44	57.65	53.98	44.39	46.52	36.55	35.82	35.28	33.17	34.55	30.34	36.45	34.11	34.3	34.12	35.24	34.3	33.33	32.03	32.47	32.1	26.81	25.05	30.07	26.38
PpeGLR2.4	40.48	40.17	41.33	40.78	35.16	39.66	60.86	60.73	57.38	100	78.66	66.23	66.78	63.96	58.35	54.48	57.04	55.09	44.05	46.51	32.66	33.74	33.26	31.57	32.51	29.24	34.45	32.12	31.63	30.97	32.11	31.52	33.62	31.24	33.43	33.17	30.26	27.76	29.38	26.98
PpeGLR2.5	40.11	41.1	42.41	40.98	35.34	39.29	60.6	60.44	58.26	78.66	100	67.51	68.82	66.77	56.95	55.21	56.33	55.53	43.76	47.34	31.98	33.37	33.33	29.65	32.1	28.84	33.72	31.58	30.91	30.24	30.92	30.35	33.05	30.09	33.24	32.33	28.52	26.49	29.33	26.99
PpeGLR2.6	39.15	41.12	42.06	41.07	35.59	39.44	59.82	61.1	57.51	66.23	67.51	100	74.12	70.49	56.66	56.1	57.08	55.97	44.32	46.94	31.79	32.92	33.1	30.86	32.24	28.92	33.12	31.92	30.37	30.01	30.8	30.14	32.96	29.99	33.33	31.51	29.02	27.1	28.5	27.53
PpeGLR2.7	40.57	40.78	42.41	41.38	36.07	40.59	63.5	63.85	59.76	66.78	68.82	74.12	100	86.05	56.6	55.28	59.28	56.2	45.55	47.33	33.93	33.74	33.91	30.39	32.59	29.29	32.95	32.31	32.14	31.6	32.18	32.03	33.46	30.77	33.98	32.38	28.61	27.45	29.13	27.08
PpeGLR2.8	36.63	36.39	39.53	36.97	37.43	36.11	60.84	60.54	60.4	63.96	66.77	70.49	86.05	100	54.87	53.36	55.27	54.97	45.03	46.43	33.8	32.87	34.71	30.29	32.26	28.55	33	33.6	31.69	31.42	31.69	31.54	31.98	28.38	30.71	30.39	27.21	26.26	29.05	27.41
PpeGLR2.9	41.07	41.69	42.92	40.78	35.99	40	59.42	61.07	58.52	58.35	56.95	56.66	56.6	54.87	100	77.24	68.46	66.6	44.47	47.55	33.57	34.47	34.74	31.28	33.14	29.75	34.47	32.56	31.73	31.19	32.51	31.84	32.6	31.13	33.79	32.37	29.15	27.96	33.1	30.4
PpeGLR2.10	39.61	40.43	40.88	40	37.13	39.86	59.51	62.23	56.44	54.48	55.21	56.1	55.28	53.36	77.24	100	68.45	66.5	44.88	46.38	31.73	32.2	33.25	32.26	31.98	29.34	33.43	32.24	30.07	29.51	30.93	30.2	33.29	32.56	34.17	32.56	28.82	27.05	33.94	30.43
PpeGLR2.11	40.21	41.79	43.3	41.58	37.29	40.07	59.08	58.61	57.65	57.04	56.33	57.08	59.28	55.27	68.46	68.45	100	86.47	47.74	48.86	33.53	34.31	33.84	31.9	32.94	29.17	33.29	32.18	33.33	32.57	33.64	33.1	32.24	29.7	31.86	30.44	28.08	26.47	32.35	29.8
PpeGLR2.12	40.87	42.03	42.06	40.83	36.06	39.76	57.66	59.1	53.98	55.09	55.53	55.97	56.2	54.97	66.6	66.5	86.47	100	45.1	46.38	32.55	33.17	33.49	30.99	32.98	29.92	33.46	32.12	32.8	32.16	33.33	32.68	31.11	29.62	31.22	30.1	27.88	26.5	32.04	29.23
PpeGLR2.13	37.7	39.22	42.63	41.18	36.59	40.1	45.55	46.9	44.39	44.05	43.76	44.32	45.55	45.03	44.47	44.88	47.74	45.1	100	91.42	33.02	34.4	33.18	30.57	32.62	29.87	33.33	31.77	32.69	32.4	33.41	32.69	30.66	29.11	31.88	30.65	27.85	26.18	29.3	27.78
PpeGLR2.14	39.56	40.53	43.75	41.96	37.69	41.06	47.05	49.53	46.52	46.51	47.34	46.94	47.33	46.43	47.55	46.38	48.86	46.38	91.42	100	33.29	34.51	34.16	30.95	34.05	31.08	34.49	33.54	33.65	33.25	33.89	33.53	31.12	30.57	32.87	31.89	28.78	26.8	30.52	28.65
PpeGLR3.1	30.04	30.8	33.98	31.44	30.76	30.95	33.26	33.61	36.55	32.66	31.98	31.79	33.93	33.8	33.57	31.73	33.53	32.55	33.02	33.29	100	64.16	55.99	50.06	57.34	54.57	59.15	54.74	61.99	61.56	62.1	62.33	30.06	26.74	30.4	28.99	24.9	25.77	28.61	26.97
PpeGLR3.2	31.33	31.33	33.92	31.73	32.19	32.25	33.33	34.95	35.82	33.74	33.37	32.92	33.74	32.87	34.47	32.2	34.31	33.17	34.4	34.51	64.16	100	58.62	50.66	58	55.37	60.82	55.51	62.31	61.86	62.78	62.66	29.95	27	30	29.12	25.81	24.57	28	26.49
PpeGLR3.3	30.53	29.54	34.39	31.93	30.61	31.46	31.13	33.56	35.28	33.26	33.33	33.1	33.91	34.71	34.74	33.25	33.84	33.49	33.18	34.16	55.99	58.62	100	49.33	54.25	51.06	57.32	52.17	55.16	54.78	55.83	55.38	29.1	25.39	30.04	28.43	25.59	25.19	29.16	28.52
PpeGLR3.4	28.74	29.84	30.32	28.72	27.92	28.36	29.77	30.63	33.17	31.57	29.65	30.86	30.39	30.29	31.28	32.26	31.9	30.99	30.57	30.95	50.06	50.66	49.33	100	46.78	46.13	47.86	45.07	48.66	48	49	48.66	28.71	26.12	28.47	28.05	23.88	24.12	29.58	28.55
PpeGLR3.5	30.69	30.62	33.97	31.88	30.77	31.09	31.23	31.34	34.55	32.51	32.1	32.24	32.59	32.26	33.14	31.98	32.94	32.98	32.62	34.05	57.34	58	54.25	46.78	100	95.07	93.28	81.56	65.47	65.36	65.17	66.25	29.62	26.49	28.71	27.76	22.79	22.77	27.95	28.15
PpeGLR3.6	27.44	27.2	30.19	29.35	27.9	28.26	28.54	28.14	30.34	29.24	28.84	28.92	29.29	28.55	29.75	29.34	29.17	29.92	29.87	31.08	54.57	55.37	51.06	46.13	95.07	100	90.44	79.09	62.11	62.02	62.03	63.11	28.43	24.47	26.67	25.93	22.41	22.09	26.02	27.39
PpeGLR3.7	30.86	30.62	32.94	31.33	30.39	30.64	32.36	32.74	36.45	34.45	33.72	33.12	32.95	33	34.47	33.43	33.29	33.46	33.33	34.49	59.15	60.82	57.32	47.86	93.28	90.44	100	81.02	66.54	66.71	66.46	67.42	31.85	26.98	28.85	28.49	23.49	23.63	30.01	29.51
PpeGLR3.8	29.27	29.16	32.32	31.59	29.61	29.71	31.34	31.93	34.11	32.12	31.58	31.92	32.31	33.6	32.56	32.24	32.18	32.12	31.77	33.54	54.74	55.51	52.17	45.07	81.56	79.09	81.02	100	61.21	60.99	61.03	61.43	29.22	27.31	28.73	28.27	24.9	22.99	27.74	27.15
PpeGLR3.9	30.75	30.08	33.6	31.5	30.09	30.45	30.49	31.58	34.3	31.63	30.91	30.37	32.14	31.69	31.73	30.07	33.33	32.8	32.69	33.65	61.99	62.31	55.16	48.66	65.47	62.11	66.54	61.21	100	98.03	96.87	98.4	28.78	26.74	29.36	28.99	23.41	23.62	26.98	25.41
PpeGLR3.10	30.39	29.68	33.39	31.53	29.5	30.19	30.15	31.26	34.12	30.97	30.24	30.01	31.6	31.42	31.19	29.51	32.57	32.16	32.4	33.25	61.56	61.86	54.78	48	65.36	62.02	66.71	60.99	98.03	100	96.9	98.47	28.48	26.23	28.9	28.42	23.84	23.65	26.16	24.61
PpeGLR3.11	31.07	30.66	33.76	31.88	30.45	30.9	30.52	32	35.24	32.11	30.92	30.8	32.18	31.69	32.51	30.93	33.64	33.33	33.41	33.89	62.1	62.78	55.83	49	65.17	62.03	66.46	61.03	96.87	96.9	100	97.3	29.28	26.7	29.78	29.37	23.81	23.93	27.39	25.9
PpeGLR3.12	30.75	30.08	33.28	31.5	30.09	30.45	30.16	31.46	34.3	31.52	30.35	30.14	32.03	31.54	31.84	30.2	33.1	32.68	32.69	33.53	62.33	62.66	55.38	48.66	66.25	63.11	67.42	61.43	98.4	98.47	97.3	100	29.03	27.12	29.64	29.11	23.54	23.62	26.51	25.28
PpeGLR4.1	32.9	31.4	31.64	31.41	27.66	30.55	34.11	33.62	33.33	33.62	33.05	32.96	33.46	31.98	32.6	33.29	32.24	31.11	30.66	31.12	30.06	29.95	29.1	28.71	29.62	28.43	31.85	29.22	28.78	28.48	29.28	29.03	100	62.26	62.6	63.47	31.07	29.45	37.14	34.34
PpeGLR4.2	28.72	28.36	28.72	28.43	24.72	27.39	29.8	29.84	32.03	31.24	30.09	29.99	30.77	28.38	31.13	32.56	29.7	29.62	29.11	30.57	26.74	27	25.39	26.12	26.49	24.47	26.98	27.31	26.74	26.23	26.7	27.12	62.26	100	78.24	77.96	32.22	30.79	35.61	33.15
PpeGLR4.3	31.17	30.28	30.84	29.99	28.48	29.45	33.1	33.29	32.47	33.43	33.24	33.33	33.98	30.71	33.79	34.17	31.86	31.22	31.88	32.87	30.4	30	30.04	28.47	28.71	26.67	28.85	28.73	29.36	28.9	29.78	29.64	62.6	78.24	100	90.58	33.8	31.51	37.39	33.53
PpeGLR4.4	30.74	29.36	31.06	29.73	26.74	29.18	31.79	31.95	32.1	33.17	32.33	31.51	32.38	30.39	32.37	32.56	30.44	30.1	30.65	31.89	28.99	29.12	28.43	28.05	27.76	25.93	28.49	28.27	28.99	28.42	29.37	29.11	63.47	77.96	90.58	100	32.41	30.57	36.62	33.11
PpeGLR4.5	26.93	26.26	26.43	26.95	23.8	25.48	28.16	27.81	26.81	30.26	28.52	29.02	28.61	27.21	29.15	28.82	28.08	27.88	27.85	28.78	24.9	25.81	25.59	23.88	22.79	22.41	23.49	24.9	23.41	23.84	23.81	23.54	31.07	32.22	33.8	32.41	100	66.96	31.58	32.03
PpeGLR4.6	24.04	24.36	23.96	23.88	23.19	22.74	24.54	25	25.05	27.76	26.49	27.1	27.45	26.26	27.96	27.05	26.47	26.5	26.18	26.8	25.77	24.57	25.19	24.12	22.77	22.09	23.63	22.99	23.62	23.65	23.93	23.62	29.45	30.79	31.51	30.57	66.96	100	29.91	31.11
PpeGLR4.7	29.04	30.76	29.2	29.42	24.42	26.92	30.89	30.4	30.07	29.38	29.33	28.5	29.13	29.05	33.1	33.94	32.35	32.04	29.3	30.52	28.61	28	29.16	29.58	27.95	26.02	30.01	27.74	26.98	26.16	27.39	26.51	37.14	35.61	37.39	36.62	31.58	29.91	100	71.29
PpeGLR4.8	26.95	28.31	27.31	28.05	23.42	26.1	28.37	27.76	26.38	26.98	26.99	27.53	27.08	27.41	30.4	30.43	29.8	29.23	27.78	28.65	26.97	26.49	28.52	28.55	28.15	27.39	29.51	27.15	25.41	24.61	25.9	25.28	34.34	33.15	33.53	33.11	32.03	31.11	71.29	100

Note: Pink background represents the most identity of gene pair.

Table S5 Particular analysis characteristics for the best theoretic of candidates duplication gene pairs in Rosaceae GLRs.

Duplicated Pair (Pear)	Duplicated model	Identities(%)	Number of conserved flanking protein-coding genes	*dN/dS*
PbrGLR1.1-PbrGLR1.2	Segmental	99.6	14	0.375
PbrGLR1.1-PbrGLR1.3	Segmental	67.14	7	0
PbrGLR2.1-PbrGLR2.4	Tandem	59.46	3	
PbrGLR2.4-PbrGLR2.7	Tandem	56.26	3	
PbrGLR2.1-PbrGLR2.3	Segmental	66.79	61	0.267
PbrGLR2.7-PbrGLR2.2	Segmental	60.44	61	0.267
PbrGLR2.3-PbrGLR2.2	Tandem	73.04	6	
PbrGLR2.2-PbrGLR2.5	Tandem	59.68	6	
PbrGLR2.5-PbrGLR2.6	Tandem	72.77	6	
PbrGLR2.6-PbrGLR2.8	Tandem	54.06	6	
PbrGLR2.8-PbrGLR2.9	Tandem	93.01	6	
PbrGLR2.7-PbrGLR2.10	Dispersed	62.87		
PbrGLR2.7-PbrGLR2.12	Dispersed	58.96		
PbrGLR2.12-PbrGLR2.15	Tandem	79.19	4	
PbrGLR2.15-PbrGLR2.13	Tandem	89.21	4	
PbrGLR2.13-PbrGLR2.11	Tandem	63.64	4	
PbrGLR2.15-PbrGLR2.14	Dispersed	92.89		
PbrGLR3.1-PbrGLR3.2	Segmental	99.26	24	0.505
PbrGLR3.2-PbrGLR3.3	Dispersed	56.82		
PbrGLR3.3-PbrGLR3.5	Dispersed	56.83		
PbrGLR3.5-PbrGLR3.7	Tandem	49.38	3	
PbrGLR3.7-PbrGLR3.4	Tandem	48.07	3	
PbrGLR3.3-PbrGLR3.9	Segmental	60.8	8	0.364
PbrGLR3.9-PbrGLR3.8	Tandem	62.51	3	
PbrGLR3.8-PbrGLR3.6	Tandem	92.65	3	
PbrGLR4.1-PbrGLR4.2	Tandem	93.35	2	
PbrGLR4.2-PbrGLR4.3	Segmental	82	11	0.167
PbrGLR4.2-PbrGLR4.5	Segmental	34.16	8	0.749
PbrGLR4.5-PbrGLR4.6	Tandem	61.49	2	
PbrGLR4.5-PbrGLR4.4	Dispersed	34.75		

Duplicated Pair (Strawberry)	Duplicated model	Identities(%)	Number of conserved flanking protein-coding genes	*dN/dS*
FveGLR1.1-FveGLR1.2	Tandem	57.86	2	
FveGLR1.3-FveGLR1.1	Dispersed	57.19		
FveGLR2.1-FveGLR2.16	Tandem	32.14	6	
FveGLR2.16-FveGLR2.3	Tandem	46.23	6	
FveGLR2.3-FveGLR2.4	Tandem	49.92	6	
FveGLR2.4-FveGLR2.6	Tandem	67.07	6	
FveGLR2.6-FveGLR2.20	Tandem	59.47	6	
FveGLR2.1-FveGLR2.2	Dispersed	69.43		
FveGLR2.1-FveGLR2.5	Dispersed	50.32		
FveGLR2.5-FveGLR2.23	Dispersed	61.41		
FveGLR2.23-FveGLR2.21	Tandem	55.74	8	
FveGLR2.21-FveGLR2.9	Tandem	45.26	8	
FveGLR2.9-FveGLR2.22	Tandem	45.56	8	
FveGLR2.22-FveGLR2.10	Tandem	43.76	8	
FveGLR2.23-FveGLR2.18	Tandem	51.03	8	
FveGLR2.18-FveGLR2.17	Tandem	62.35	8	
FveGLR2.17-FveGLR2.19	Tandem	48.2	8	
FveGLR2.9-FveGLR2.12	Dispersed	59.47		
FveGLR2.12-FveGLR2.11	Tandem	69.14	2	
FveGLR2.4-FveGLR2.14	Dispersed	62.5		
FveGLR2.14-FveGLR2.13	Dispersed	60.8		
FveGLR2.13-FveGLR2.15	Tandem	76.11	2	
FveGLR3.1-FveGLR3.2	Dispersed	56.79		
FveGLR4.1-FveGLR4.2	Segmental	35.15	7	0.735
FveGLR4.2-FveGLR4.3	Tandem	73.91	2	
FveGLR4.3-FveGLR4.8	Dispersed	32.87		
FveGLR4.8-FveGLR4.5	Tandem	57.85	2	
FveGLR4.8-FveGLR4.7	Segmental	87.38	42	0.151
FveGLR4.5-FveGLR4.4	Segmental	86.97	42	0.151
FveGLR4.4-FveGLR4.7	Tandem	63.97	2	

Duplicated Pair (Plum)	Duplicated model	Identities(%)	Number of conserved flanking protein-coding genes	*dN/dS*
PmuGLR1.2-PmuGLR1.3	Dispersed	94.06		
PmuGLR1.3-PmuGLR1.5	Dispersed	88.08		
PmuGLR1.5-PmuGLR1.1	Tandem	85	2	
PmuGLR1.5-PmuGLR1.4	Dispersed	83.52		
PmuGLR2.1-PmuGLR2.2	Tandem	73.25	9	
PmuGLR2.2-PmuGLR2.4	Tandem	59.31	9	
PmuGLR2.4-PmuGLR2.5	Tandem	77.85	9	
PmuGLR2.5-PmuGLR2.6	Tandem	69.35	9	
PmuGLR2.6-PmuGLR2.8	Tandem	76	9	
PmuGLR2.8-PmuGLR2.10	Tandem	48.08	9	
PmuGLR2.10-PmuGLR2.3	Tandem	52.45	9	
PmuGLR2.3-PmuGLR2.9	Tandem	59.72	9	
PmuGLR2.8-PmuGLR2.7	Dispersed	82.83		
PmuGLR2.9-PmuGLR2.12	Dispersed	64.9		
PmuGLR2.12-PmuGLR2.13	Tandem	87.72	2	
PmuGLR2.13-PmuGLR2.11	Dispersed	72.27		
PmuGLR3.1-PmuGLR3.2	Dispersed	59.53		
PmuGLR3.2-PmuGLR3.6	Dispersed	62.07		
PmuGLR3.6-PmuGLR3.4	Tandem	56.32	2	
PmuGLR3.6-PmuGLR3.7	Dispersed	91.68		
PmuGLR3.6-PmuGLR3.3	Dispersed	54.25		
PmuGLR3.3-PmuGLR3.5	Tandem	51.16	2	
PmuGLR4.1-PmuGLR4.4	Tandem	60.65	4	
PmuGLR4.4-PmuGLR4.2	Tandem	84.3	4	
PmuGLR4.2-PmuGLR4.3	Tandem	84.63	4	
PmuGLR4.2-PmuGLR4.5	Dispersed	37.28		
PmuGLR4.5-PmuGLR4.8	Tandem	33.41	2	
PmuGLR4.2-PmuGLR4.7	Segmental	37.48	8	0.684
PmuGLR4.7-PmuGLR4.8	Tandem	72.94	2	

Duplicated Pair (Peach)	Duplicated model	Identities(%)	Number of conserved flanking protein-coding genes	*dN/dS*
PpaGLR1.1-PpaGLR1.3	Tandem	67.64	6	
PpaGLR1.3-PpaGLR1.5	Tandem	91.1	6	
PpaGLR1.5-PpeGLR1.6	Tandem	90.78	6	
PpaGLR1.6-PpeGLR1.4	Tandem	89.06	6	
PpaGLR1.4-PpeGLR1.2	Tandem	67.64	6	
PpaGLR2.1-PpaGLR2.2	Tandem	72.04	9	
PpaGLR2.2-PpaGLR2.5	Tandem	60.44	9	
PpaGLR2.5-PpaGLR2.4	Tandem	78.66	9	
PpaGLR2.4-PpaGLR2.6	Tandem	66.23	9	
PpaGLR2.6-PpaGLR2.7	Tandem	74.12	9	
PpaGLR2.7-PpaGLR2.10	Tandem	55.28	9	
PpaGLR2.10-PpaGLR2.3	Tandem	56.44	9	
PpaGLR2.3-PpaGLR2.9	Tandem	58.52	9	
PpaGLR2.7-PpaGLR2.8	Dispersed	86.05		
PpaGLR2.11-PpaGLR2.12	Tandem	86.47	2	
PpaGLR2.9-PpaGLR2.11	Dispersed	68.46		
PpaGLR2.13-PpaGLR2.14	Tandem	91.42	2	
PpaGLR3.1-PpaGLR3.2	Dispersed	64.16		
PpaGLR3.2-PpaGLR3.4	Dispersed	50.66		
PpaGLR3.4-PpaGLR3.3	Tandem	49.33	2	
PpaGLR3.2-PpaGLR3.11	Dispersed	62.78		
PpaGLR3.11-PpaGLR3.12	Dispersed	97.3		
PpaGLR3.12-PpaGLR3.5	Tandem	66.25	6	
PpaGLR3.5-PpaGLR3.8	Tandem	81.56	6	
PpaGLR3.8-PpaGLR3.7	Tandem	81.02	6	
PpaGLR3.7-PpaGLR3.9	Tandem	66.54	6	
PpaGLR3.9-PpaGLR3.6	Tandem	62.11	6	
PpaGLR4.1-PpaGLR4.2	Tandem	62.26	4	
PpaGLR4.2-PpaGLR4.3	Tandem	78.24	4	
PpaGLR4.3-PpaGLR4.4	Tandem	90.58	4	
PpaGLR4.3-PpaGLR4.5	Dispersed	33.8		
PpaGLR4.5-PpaGLR4.6	Tandem	66.96	2	
PpaGLR4.1-PpaGLR4.8	Segmental	34.34	10	0.816
PpaGLR4.7-PpaGLR4.8	Tandem	71.29	2	

Note: The blue background in the table representative the tandem duplication string.

Table. S6 The orthology of *GLR* genes in four Rosaceae species

Fve	Pmu	Ppe	Pbr	Pmu	Ppe	Pbr	Fve	Ppe	Pbr	Fve	Pmu	Pbr	Fve	Pmu	Ppe
1.1	\	\	\	1.1	\	\	\	1.1	\	\	\				
1.2	1.2(13)	1.2(37)	1.1(28)-1.2(8)-1.3(7)	1.2	1.2(46)	1.1(16)	1.2(13)	1.2	1.2(74)	1.2(37)	1.2(46)	1.1	1.2(28)	1.2(16)	1.2(74)
1.3	\	\	\	1.3	\	\	\	1.3	\	\	\	1.2	1.2(8)	1.2(10)	1.2(12)
				1.4	\	\	\	1.4	\	\	\	1.3	1.2(7)	1.2(12)	1.2(10)
				1.5	\	\	\	1.5	\	\	\				
								1.6	\	\	\				

Fve	Pmu	Ppe	Pbr	Pmu	Ppe	Pbr	Fve	Ppe	Pbr	Fve	Pmu	Pbr	Fve	Pmu	Ppe
2.1	\	\	\	2.1	\	\	\	2.1	2.1(471)-2.3(61)	\	\	2.1	2.22 (163)	2.3(395)	2.1(471)
2.2	\	\	\	2.2	\	2.3(50)	2.10(12)	2.2	\	\	\	2.2	\	\	\
2.3	\	\	\	2.3	2.3(621)	2.4(384)	\	2.3	\	\	2.3(621)	2.3	2.21(34)	2.2(50)	2.1(61)
2.4	\	\	\	2.4	\	\	\	2.4	\	\	\	2.4	\	\	\
2.5	\	\	\	2.5	\	\	\	2.5	2.7(471)	\	2.6(621)	2.5	\	\	\
2.6	\	\	\	2.6	2.5(621)	\	\	2.6	\	\	\	2.6	\	2.8(50)	\
2.7	\	\	\	2.7	\	\	\	2.7	\	\	\	2.7	\	\	2.5(471)
2.8	\	\	\	2.8	\	2.6(50)	2.22(12)	2.8	\	\	\	2.8	\	2.9(50)	\
2.9	\	\	\	2.9	\	2.8(50)	\	2.9	\	\	\	2.9	\	\	\
2.10	2.2(196)	2.10(13)	\	2.10	\	\	\	2.10	\	2.10(13)	\	2.10	\	\	\
2.11	2.13(144)	2.12(250)	\	2.11	\	\	\	2.11	\	\	\	2.11	\	\	\
2.12	\	\	\	2.12	\	\	\	2.12	\	2.11(250)	2.13(621)	2.12	\	\	\
2.13	\	\	\	2.13	2.12(621)	\	2.11(129)	2.13	\	\	\	2.13	\	\	\
2.14	\	\	\	2.14	\	\	\	2.14	\	\	\	2.14	\	\	\
2.15	\	\	\									2.15	\	\	\
2.16	\	\	\												
2.17	\	\	\												
2.18	\	\	\												
2.19	\	\	\												
2.20	\	\	\												
2.21	\	\	2.3(34)												
2.22	2.8(12)	2.3(13)	2.1(163)												
2.23	\	\	\												

Fve	Pmu	Ppe	Pbr	Pmu	Fve	Ppe	Pbr	Ppe	Pmu	Pbr	Fve	Pbr	Ppe	Pmu	Fve
3.1	3.2(7)	3.1(208)-3.2(8)-3.6(30)	3.1(15)-3.2(14)	3.1	3.1(71)	3.1(81)-3.2(8)-3.6(8)	3.1(23)-3.2(36)-3.9(7)	3.1	3.1(81)	3.2(97)	3.1(208)	3.2	3.1(97)	3.1(36)	3.1(14)
3.2	3.2(50)	3.2(52)-3.6(8)	3.9(7)-3.10(6)	3.2	3.2(135)-3.1(7)	3.1(14)-3.2(282)-3.6(10)	3.3(46)-3.9(9)-3.1(8)	3.2	3.1(8)-3.2(283)	3.3(44)-3.9(10)-3.10(8)	3.2(52)-3.1(8)	3.3	3.2(44)-3.1(9)	3.2(46)	\
mrna23938	3.5(36)	3.4(60)	3.4(48)	3.5	mrna23938 (36)	3.4(51)	3.4(95)	3.4	3.5(115)	3.4(84)	mrna23938(127)	3.4	3.4(84)	3.5(95)	mrna23938(48)
				3.3	\	\	\	3.3	\	\	\	3.1	3.1(7)	3.1(23)	3.1(13)
				3.4	\	\	\	3.5	\	\	\	3.5	\	\	\
mrna13028	3.4/3.6(84)-3.2(9)-3.1(6)	3.6(215)-3.2(10)-3.1(7)	3.9(81)-3.10(43)	Pm26785	mrna13028(84)	3.6(105)		3.6	Pm026785(712)-3.2(10)-3.1(8)	3.9(102)-3.10(55)	mrna13028(343)-3.1(10)-3.2(8)	3.9	3.6(102)-Ppe3.1(7)-3.2(10)	3.1(7)-3.2(9)-3.6(102)	mrna13028(81)
				3.6	\	\	3.10(51)-3.9(14)	3.7	\	\	\	3.6	\	\	\
				3.7	\	\	\	3.8	\	\	\	3.7	\	\	\
								3.9	\	\	\	3.8	\	\	\
								3.10	\	\	\	3.10	3.2(8)-3.6(55)	3.2(8)-3.6(55)	mrna13028(6)
								3.11	\	\	\				
								3.12	\	\	\				

Fve	Pmu	Ppe	Pbr	Pmu	Ppe	Fve	Pbr	Ppe	Pbr	Fve	Pmu	Pbr	Fve	Pmu	Ppe
4.1	\	\	\	4.1	4.7(9)	4.8(8)	\	4.1	4.2(100)-4.3(63)	4.5(8)	\	4.1	\	\	\
4.2	\	\	\	4.2	\	\	\	4.2	4.5(7)	\	\	4.2	4.8(6)	\	4.1(100)-4.8(11)
				4.3	4.8(9)	\	4.3(23)-4.5(7)	4.3	\	\	\	4.3	\	4.3(23)	4.1(63)
				4.4	\	\	\	4.4	\	\	\				
								4.5	\	\	\				
4.3	4.5(185)	4.6(193)	4.4(42)	4.5	4.6(1074)	4.3(185)	4.4(503)	4.6	4.4(478)	4.3(193)	4.5(1074)	4.4	4.3(42)	4.5(503)	4.6(478)
4.4	\	\	\	4.6	\	\	\	4.7	\	\	4.1(9)	4.5	4.8(98)	4.3(7)	4.2(7)
4.5	\	4.1(8)	\	4.7	\	\	\	4.8	4.2(11)	\	4.8(375)-4.3(9)	4.6	\	\	\
4.6	\	\	\	4.8	4.8(375)	\	\								
4.7	\	\	\												
4.8	4.1(8)	\	4.2(6)-4.5(98)												

Note: In order to show concise, we simplify the gene name, the GLR is saved. Number in bracket represent that nunber of homologous gene pairs in the synteny bulk between two Rosaceae species. "\" repesent that none homologous gene pairs between two Rosaceae species. Aplurality of data in a square repesent that one gene have multiple synteny bulks at the same time. The orange background repesent that the homologous gene pair was determined.

Table S7. Statistics percentage of tandem genes in each family.

Species	Short name	GLR0			GLR1&2			GLR3			GLR4		
		Number of tandem genes	Number of Total genes	Percentage of tandem genes	Number of tandem genes	Number of Total genes	Percentage of tandem genes	Number of tandem genes	Number of Total genes	Percentage of tandem genes	Number of tandem genes	Number of Total genes	Percentage of tandem genes
Coccomyxa subellipsoidea C-169	Csu	0	1	0%									
Chlamydomonas reinhardtii	Cre	0	3	0%									
Volvox carteri	Vca	0	2	0%									
Ostreococcus lucimarinus	Olu												
Micromonas pusilla CCMP1545	Mpu												
Physcomitrella patens	Ppa	0	2	0%									
Selaginella moellendorffii	Smo	0	2	0%									
Amborella trichopoda	Atr				6	8	75.00%	0	2	0%	4	4	100.00%
Spirodela polyrhiza	Spo				6	8	75.00%	0	3	0%	0	1	0%
Oryza sativa	Osa				13	14	92.86%	0	5	0%	0	1	0%
Brachypodium distachyon	Bdi				10	14	71.43%	0	5	0%			
Setaria italica	Sit				11	12	91.67%	3	8	37.50%			
Sorghum bicolor	Sbi				13	14	92.86%	7	9	77.78%			
Zea mays	Zma				2	5	40.00%	4	8	50.00%			
Aquilegia coerulea	Aco				12	15	80.00%	4	4	100.00%	8	9	88.89%
Beta vulgaris	Bvu				2	4	50.00%	4	6	66.67%	2	3	66.67%
Actinidia chinensis	Ach				6	8	75.00%	4	9	44.44%	6	11	54.55%
Salanum lycopersicum	Sly				5	6	83.33%	3	5	60.00%	2	2	100.00%
Solanum tuberosum	Stu				2	4	50.00%	2	2	100.00%	2	4	50.00%
Vitis vinifera	Vvi				9	9	100.00%	2	5	40.00%	2	3	66.67%
Citrus sinensis	Csi				10	11	90.91%	4	6	66.67%	2	3	66.67%
Theobroma cacao	Tca				9	12	75.00%	4	6	66.67%	8	9	88.89%
Eucalyptus grandis	Egr				42	49	85.71%	0	4	0%	0	5	0%
Brassica rapa	Bra				10	18	55.56%	4	11	36.36%			
Eutrema salsugineum	Esa				2	8	25.00%	2	7	28.57%			
Arabidopsis thaliana	Ath				11	13	84.62%	2	7	28.57%			
Arabidosis lyrata	Aly				12	18	66.67%	0	8	0%			
Capsella rubella	Cru				6	12	50.00%	2	7	28.57%			
Capsella grandiflora	Cgr				5	11	45.45%	2	7	28.57%			
Cucumis sativus	Csa				6	11	54.55%	3	7	42.86%	8	9	88.89%
Medicago truncatula	Mtr				2	3	66.67%	2	6	33.33%	9	11	81.82%
Glycine max	Gma				2	4	50.00%	7	16	43.75%	9	14	64.29%
Phaseolus vulgaris	Pvu				3	3	100.00%	4	9	44.44%	4	4	100.00%
Fragaria vesca	Fve				21	26	80.77%	2	2	100.00%	6	8	75.00%
Pyrus bretchneideri	Pbr				13	18	72.22%	6	10	60.00%	4	6	66.67%
Prunus persica	Ppe				19	20	95.00%	8	12	66.67%	8	8	100.00%
Prunus mume	Pmu				13	19	68.42%	4	7	57.14%	8	8	100.00%
Linum usitatissimum	Lus				0	5	0%	8	13	61.54%	4	4	100.00%
Poulus trichocarpa	Ptr				35	36	97.22%	4	8	50.00%	15	16	93.75%
Salix purpurea	Spu				14	19	73.68%	7	14	50.00%	14	20	70.00%
Manihot esculenta	Mes				0	4	0%	2	9	22.22%	3	3	100.00%
Ricinus communis	Rco				7	7	100.00%	2	5	40.00%	2	5	40.00%
Total		0	10	0%	339	448	75.67%	112	252	44.44%	130	171	76.02%

Table S8 Statistics on absence conserve domain for four Rosaceae species and *Arabidopsis* .

Species	PF01094	PF00060	GlnH1	GlnH2	M1	M2	M3	M4
***Arabidopsis thaliana* (20)**	0	0	0	5% (1)	0	5% (1)	15% (3)	0
***Pyrus brechneideri* (34)**	2.94% (1)	0	0	2.94% (1)	0	5.88% (2)	5.88% (2)	2.94% (1)
***Fragaria vesca* (36)**	61.11% (22)	0	2.78% (1)	5.56% (2)	11.11% (4)	16.67% (6)	16.67% (6)	5.56% (2)
***Prunus persica* (40)**	0	0	7.3%(3)	2.5% (1)	0	10% (4)	0	2.5% (1)
***Prunus mume* (34）**	0	0	2.94% (1)	2.94% (1)	2.94% (1)	2.94% (1)	8.82% (3)	2.94% (1)
***Rosaceae* (144)**	15.97% (23)	0	3.47% (5)	3.47% (5)	3.47% (5)	9.03% (13)	7.64% (11)	3.47% (5)

Note: The numbers in brackets indication that the number of lack of the corresponding domain in GLR genes for each plant species.

Table S9 Statistics on absence conserve domain for each Rosaceae species and arabidopsis in detail to subfamily

Species	Subfamily	PF01094	PF00060	GlnH1	GlnH2	M1	M2	M3	M4
***Arabidopsis thaliana* (20)**	Group1&2	0	0	0	5% (1)	0	5% (1)	15% (3)	0
	Group3	0	0	0	0	0	0	0	0
***Pyrus brechneideri* (34)**	Group1&2	2.94% (1)	0	0	2.94% (1)	0	5.88% (2)	2.94% (1)	2.94% (1)
	Group3	0	0	0	0	0	0	2.94% (1)	0
	Group4	0	0	0	0	0	0	0	0
***Fragaria vesca* (36)**	Group1&2	61.11% (22)	0	2.78% (1)	5.56% (2)	8.33% (3)	11.11% (4)	11.11% (4)	5.56% (2)
	Group3	0	0	0	0	0	2.78% (1)	2.78% (1)	0
	Group4	0	0	0	0	2.78% (1)	2.78% (1)	2.78% (1)	0
***Prunus persica* (40)**	Group1&2	0	0	0	2.5% (1)	0	2.5% (1)	0	2.5% (1)
	Group3	0	0	2.5% (1)	0	0	2.5% (1)	0	0
	Group4	0	0	5% (2)	0	0	5% (2)	0	0
***Prunus mume* (34）**	Group1&2	0	0	2.94% (1)	2.94% (1)	2.94% (1)	2.94% (1)	5.88% (2)	2.94% (1)
	Group3	0	0	0	0	0	0	2.94% (1)	0
	Group4	0	0	0	0	0	0	0	0
***Rosaceae* (144)**	Group1&2	15.97% (23)	0% (0)	1.39% (2)	3.47% (5)	2.78% (4)	5.56% (8)	4.86% (7)	3.47% (5)
	Group3	0% (0)	0% (0)	0.69% (1)	0% (0)	0% (0)	1.39% (2)	2.08% (3)	0% (0)
	Group4	0% (0)	0% (0)	1.39% (2)	0% (0)	0.69% (1)	2.08% (3)	0.69% (1)	0% (0)

Note: The numbers in brackets indication that the number of lack of the corresponding domain in GLR genes for each plant species.

Table S10. Statistics percentage of lost ATD genes in each family.

Species	Short name	GLR0			GLR1&2			GLR3			GLR4		
		Number of lost ATD genes	Number of Total genes	Percentage of lost ATD genes	Number of lost ATD genes	Number of Total genes	Percentage of lost ATD genes	Number of lost ATD genes	Number of Total genes	Percentage of lost ATD genes	Number of lost ATD genes	Number of Total genes	Percentage of lost ATD genes
Coccomyxa subellipsoidea C-169	Csu	1	1	100%									
Chlamydomonas reinhardtii	Cre	3	3	100%									
Volvox carteri	Vca	2	2	100%									
Ostreococcus lucimarinus	Olu												
Micromonas pusilla CCMP1545	Mpu												
Physcomitrella patens	Ppa	0	2	0%									
Selaginella moellendorffii	Smo	0	2	0%									
	Pab	3	13	23%									
	Pta	0	13	0%									
Amborella trichopoda	Atr				0	8	0.00%	0	2	0.00%	0	4	0.00%
Spirodela polyrhiza	Spo				0	8	0.00%	0	3	0.00%	0	1	0.00%
Oryza sativa	Osa				5	14	35.71%	0	5	0.00%	0	1	0.00%
Brachypodium distachyon	Bdi				3	14	21.43%	0	5	0.00%			
Setaria italica	Sit				3	12	25.00%	0	8	0.00%			
Sorghum bicolor	Sbi				2	14	14.29%	0	9	0.00%			
Zea mays	Zma				1	5	20.00%	0	8	0.00%			
Aquilegia coerulea	Aco				0	15	0.00%	0	4	0.00%	0	9	0.00%
Beta vulgaris	Bvu				2	4	50.00%	0	6	0.00%	0	3	0.00%
Actinidia chinensis	Ach				3	8	37.50%	2	9	22.22%	0	11	0.00%
Salanum lycopersicum	Sly				0	6	0.00%	0	5	0.00%	0	2	0.00%
Solanum tuberosum	Stu				0	4	0.00%	0	2	0.00%	1	4	25.00%
Vitis vinifera	Vvi				0	9	0.00%	0	5	0.00%	0	3	0.00%
Citrus sinensis	Csi				2	11	18.18%	0	6	0.00%	0	3	0.00%
Theobroma cacao	Tca				2	12	16.67%	0	6	0.00%	5	9	55.56%
Eucalyptus grandis	Egr				4	49	8.16%	0	4	0.00%	1	5	20.00%
Brassica rapa	Bra				4	18	22.22%	0	11	0.00%			
Eutrema salsugineum	Esa				0	8	0.00%	0	7	0.00%			
Arabidopsis thaliana	Ath				0	13	0.00%	0	7	0.00%			
Arabidosis lyrata	Aly				1	18	5.56%	1	8	12.50%			
Capsella rubella	Cru				0	12	0.00%	0	7	0.00%			
Capsella grandiflora	Cgr				0	11	0.00%	0	7	0.00%			
Cucumis sativus	Csa				2	11	18.18%	0	7	0.00%	0	9	0.00%
Medicago truncatula	Mtr				1	3	33.33%	0	6	0.00%	0	11	0.00%
Glycine max	Gma				2	4	50.00%	1	16	6.25%	2	14	14.29%
Phaseolus vulgaris	Pvu				3	3	100.00%	0	9	0.00%	0	4	0.00%
Fragaria vesca	Fve				22	26	84.62%	0	2	0.00%	0	8	0.00%
Pyrus bretchneideri	Pbr				1	18	5.56%	0	10	0.00%	0	6	0.00%
Prunus persica	Ppe				0	20	0.00%	0	12	0.00%	0	8	0.00%
Prunus mume	Pmu				2	19	10.53%	0	7	0.00%	0	8	0.00%
Linum usitatissimum	Lus				1	5	20.00%	0	13	0.00%	1	4	25.00%
Poulus trichocarpa	Ptr				0	36	0.00%	0	8	0.00%	0	16	0.00%
Salix purpurea	Spu				1	19	5.26%	0	14	0.00%	0	20	0.00%
Manihot esculenta	Mes				0	4	0.00%	0	9	0.00%	1	3	33.33%
Ricinus communis	Rco				7	7	100.00%	0	5	0.00%	2	5	40.00%
Total		9	36	25.00%	74	448	16.52%	4	252	1.59%	13	171	7.60%

Table S11. Amino acid sequence identity with each GLR subfamily in analysed angiosperm species.

Scientific name	Short name	GLR1&2	GLR3	GLR4
Amborella trichopoda	Atr	52.06%	76.96%	54.21%
Spirodela Polyrhiza	Spo	38.63%	55.60%	/
Oryza sativa	Osa	26.86%	56.85%	/
Brachypodium distachyon	Bdi	31.40%	58.49%	/
Setaria italica	Sit	25.75%	65.40%	/
Sorghum bicolor	Sbi	22.33%	61.68%	/
Zea mays	Zma	30.73%	61.59%	/
Aquilegia coerulea	Aco	39.33%	63.53%	47.03%
Beta vulgaris	Bvu	29.31%	62.85%	58.71%
Actinidia chinensis	Ach	27.16%	43.66%	30.07%
Solanum lycopersicum	Sly	63.72%	62.46%	27.78%
Solanum tuberosum	Stu	43.30%	52.09%	55.22%
Vitis vinifera	Vvi	44.80%	45.03%	28.71%
Citrus sinensis	Csi	35.62%	64.10%	39.69%
Theobroma cacao	Tca	35.85%	65.74%	27.48%
Eucalyptus grandis	Egr	25.40%	64.46%	67.24%
Brassica rapa FPsc v1.3	Bra	28.01%	61.91%	/
Eutrema salsugineum	Esa	38.49%	55.40%	/
Arabidopsis thaliana	Ath	38.60%	60.17%	/
Arabidopsis lyrata v1.0	Aly	29.58%	56.98%	/
Capsella rubella	Cru	36.41%	59.34%	/
Capsella grandiflora	Cgr	28.83%	60.32%	/
Cucumis sativus	Csa	32.52%	61.50%	41.45%
Medicago truncatula	Mtr	46.72%	61.66%	42.55%
Glycine max	Gma	28.50%	59.41%	28.59%
Phaseolus vulgaris	Pvu	57.71%	73.54%	36.79%
Fragaria vesca	Fve	25.11%	48.79%	40.44%
Pyrus bretchneideri	Pbr	43.85%	64.18%	50.18%
Prunus persica	Ppe	37.17%	68.67%	48.78%
Prunus mume	Pmu	30.74%	48.70%	48.81%
Linum usitatissimum	Luc	55.16%	56.83%	40.70%
Populus trichocarpa	Ptr	46.83%	60.52%	46.03%
Salix purpurea	Spu	37.78%	65.94%	24.62%
Manihot esculenta	Mes	44.51%	64.76%	24.02%
Ricinus communis	Rco	51.82%	64.07%	25.82%
Average		37.45%	60.38%	40.65%
With all GLRs genes		10.80%	29.76%	13.76%

Note: "/" represent absent or only one GLR genes in this subfamily.

Table S12. Nonsynonymous to synonymous substitution rates of GLR genes.

Order	d_N/d_S		
	**GLR1&2**	**GLR3**	**GLR4**
**Amborellales**	0.05346	/	0.09583
**Alismatales**	0.06328	0.03811	/
**Poales**	0.08634	0.07064	/
**Ranunculales (Stem eudicotyledons)**	0.18343	0.04954	0.13211
**Caryophyllales**	0.19432	0.03574	0.14215
**Asterids**	0.23537	0.05159	0.14462
**Vitales**	0.23358	0.04574	0.140157
**Sapindales**<br>**Malvales**<br>**Myrtales**	0.27134	0.04413	0.18598
**Brassicales**	0.213864	0.05152	/
**Cucurbitales**	0.22549	0.04841	0.16482
**Fabales**	0.21486	0.04549	0.18546
**Rosales**	0.26345	0.04418	0.18249
**Malpighiales**	0.28481	0.06047	0.17567

Note: "/" represent absent or only one GLR genes in this subfamily.

Table S13. The primers of pear GLRs for qRT-PCR.

Primer	Gene Name
CGTCGAGATCACACAGGATGAA	PbrGLR1.1&1.2-F
TCTGTTCGTTTGTCGAGGAGG	PbrGLR1.1&1.2-R
AGAAGCTAGCCATCCTCTCGAT	PbrGLR1.3-F
TGTCAAGAATCAACCCGACATGA	PbrGLR1.3-R
TTTGTCATTGGACAGAAGTCGCA	PbrGLR2.1-F
AAGTCCGAGAGCGCCATTTC	PbrGLR2.1-R
ACAGAACACAACAATCCCGGT	PbrGLR2.2-F
AGAGGAAGCACGGGAGGTAT	PbrGLR2.2-R
CTCAGGCCTTTCAGACCACC	PbrGLR2.3-F
GCTGTTGCGGAACTCTGTCT	PbrGLR2.3-R
AGCGGCAGTCTTGGCCTTG	PbrGLR2.4-F
TCCAGCCAGTTGCTATCAGG	PbrGLR2.4-R
CAATCCGACCATGAATGTGATT	PbrGLR2.5-F
GAAGTCGCCCATCGACAAGAC	PbrGLR2.5-R
AGCACTAACACAACGGAAGTCA	PbrGLR2.6-F
ATCATCGTAAGTGCCAGCCA	PbrGLR2.6-R
ATGGAGGTAGGGTATGCTTG	PbrGLR2.7-F
TCCGAACACATCCAGTTTAAC	PbrGLR2.7-R
TGGTATGCTTGGATAATGACTGA	PbrGLR2.8-F
CGAAGATGTTGACCGGGACT	PbrGLR2.8-R
TCGGCATGATGAAGGCAGAT	PbrGLR2.9-F
TCTTTGACAAGCTGAGGACCAT	PbrGLR2.9-R
AATGCCAGTAGTCTTGGACCT	PbrGLR2.10-F
CCACGCTTCACTGGAACTAG	PbrGLR2.10-R
AGACCGCACTTACCATGCT	PbrGLR2.11-F
AGAGCTTGACGACGGCCTTG	PbrGLR2.11-R
TATGTTGGCTTCAAAGCAGGT	PbrGLR2.12-F
AAAATCGTGCGTGAGAAGCG	PbrGLR2.12-R
GCTAGGTTTGTAGTGGTCATCTGG	PbrGLR2.13-F
TCAGTAACGACTGGGTTGAGC	PbrGLR2.13-R
ACCAGCTGCTGCTTGTTTATT	PbrGLR2.14&2.15-F
CAGCACTAGCTCCCGATGTA	PbrGLR2.14&2.15-R
CATTGGAGCGTTAAAGTACATGG	PbrGLR3.1&3.2-F
AGCGCCGTGACTGACAATAAC	PbrGLR3.1&3.2-R
TTCAAGCTCTGCAATTCATG	PbrGLR3.3-F
TGGCTGCAAATGACAATAAG	PbrGLR3.3-R
ATGGAGGTATTGCTGATGAATAGG	PbrGLR3.4-F
GCAGCTAAAATTGCCGGCTT	PbrGLR3.4-R
CCCAGGTTGCGCAAAATAACT	PbrGLR3.5-F
ACCTGTGACCAACCACATCT	PbrGLR3.5-R
TGCCGTACGAGTTTGTACCT	PbrGLR3.6&3.8-F
GAAAATGCCTGTGACACCCC	PbrGLR3.6&3.8-R
TGAAGATGGTTGCAGAAAACGTG	PbrGLR3.7-F
GTAGCGAGCTGCCTCTTAGG	PbrGLR3.7-R
AGTATAGTTTGGCTCCAGGTGT	PbrGLR3.9-F
CCACTGTAATTTGAGTCCTGCT	PbrGLR3.9-R
CACTGTAGCGGCAACGGATT	PbrGLR3.10-F
ACCATTTGAGGCAAACCCACT	PbrGLR3.10-R
ATGTCTCAGTTTGCTCTTCTTTTT	PbrGLR4.1&4.2-F
GAGGGCTTCCTCTGAGAGT	PbrGLR4.1&4.2-R
AATTCGTCCTAGAGGTTCAATCA	PbrGLR4.3-F
AGGGCTCCCTCTGAGAGTTT	PbrGLR4.3-R
GAGCAACAGCTGCAGCAATAA	PbrGLR4.4-F
TCGCCATCGGAAATGACCAA	PbrGLR4.4-R
TCTGCTGCTGAAGAATTGATAAAG	PbrGLR4.5-F
CATCGGCGTTGCATTAGTGG	PbrGLR4.5-R
TGCTGCTTATGCCGCTAAAGAATTA	PbrGLR4.6-F
AGAACCGTCTGCTGCCATTC	PbrGLR4.6-R
GGAGCTGAGAGATTCCGGTG	PbrACT-F
TACGGTCTGCAATACCTGGGAA	PbrACT-R
GCTGACAAAACCCCGAAAA	PbrCYP-F
CGATGAAATGTGGAGCCCTTA	PbrCYP-R
GGTGTGAAGCAGATGATTTG	PbrEF1α-F
TCACCCTCAAACCCAGATAT	PbrEF1α-R
TGGTGTGAACGAGAAGGAAT	PbrGAPDH-F
CCCTCAACAATCCCAAACC	PbrGAPDH-R
TGGGCTTTGCTCCTCTTAC	PbrTub-2-F
CCTTCGTGCTCATCTTACC	PbrTub-2-R

MIQE checklist

1. Experimental design

Expression analysis of pear GLRs in different tissues (Group 1), vascular tissues development (Group 2) and shoot tip sprouting (Group 3). Each groups consisting of four independent experiments.

2. Sample

A total of 18 samples in three groups (Group1-3). Tissue expression pattern analysis (Group 1) consisting of seven tissues or organs include root, stem, leaf, seed, fruit, pistil and pollen (S1-S7). Vascular tissues development analysis (Group 2) consisting of three development phases of phloem, cambium and xylem (S8-S16). Shoot tip sprouting analysis consisting of sprouting shoot tip and dormant shoot tip (S17, S18). Detail description of samples S1-S18 is shown in Table1. The samples were frozen and storage as soon as were collected by liquid nitrogen. Samples storage duration not more than 1 day, then were extracted nucleic acid.

3. Nucleic acid extraction and reverse transcription

Total RNA was extracted using the Trizol reagent (Takara, Japan) according to the manufacturer's method. RNA samples were assessed with OD 260/280 > 2.0 and OD 260/230 > 1.8 (Table 2). The RNA integrity of samples were detected by electrophoresis traces, the result showed that all samples contain 28 s and 18 s and no dispersion band (Fig. 1), indicated that it is has not occurred the degradation and integrity in nucleic acid extraction of all samples. Equal amounts of total RNA (2 μg) in all samples were treated with DNAase I (TaKaRa) for 10 min at 65 ^{o}C to eliminate genomic DNA contamination, and then used for cDNA synthesis using a PrimeScript^{TM} RT Reagent Kit (Perfect Real Time; TaKaRa). Purified cDNA samples were diluted 1:20 with RNase-free water before use as templates in the qRT-PCR process. RNA extraction and cDNA synthesis from all samples was performed with three biological replicates. Then, one reference gene which the primer spanned an intron as described previously[1], *PbrEF1α*, were used to verify all cDNA samples' quality by PCR before carrying out qRT-PCR. There was no genomic DNA contamination in cDNA templates (Fig. S1). Neither primer dimers nor unexpected products were found. The results suggested

that all cDNA were of sufficient quality for qRT-PCR.

4. RT-qPCR target information

RT-qPCR primers for the pear GLRs were designed using primer-blast tool (http://www.ncbi.nlm.nih.gov/tools/primer-blast/) with melting temperature within 56-62 °C and a primer length of 18-26 bp. There is no homology to pseudogenes or other unexpected targets in primer blast assay. Detail description of primer information is shown in Table 3. Each primer position and spanned exon or intron is illustrated in Fig. 3.

5. RT-qPCR protocol

To assay the gene expression in pear, 20 μl of mixture corresponds to 5 ng of total cDNA in one PCR reaction system (Takara SYBR PrimeScript RT-PCR Kit for Perfect Real Time). The reaction was carried out in qRT-PCR equipment (Roche LightCycler® 480II) according to the method of the manufacturer (Takara). Reaction mixtures were incubated for 10 min at 95 °C for pre-incubation followed by 45 amplification cycles of 15 s at 95 °C, 15 s at 60 °C and 20 s at 72 °C. After that, a dissociation curve was generated at 60-95 °C. Three biological replicates were amplified for all samples. Lin-RegPCR was used to calculate the efficiency of the primers in RT-qPCR (Ramakers *et al.*, 2003). Expression levels GLR genes in all samples were determined by their quantification cycle values (Cqs).

6. RT-qPCR validation

To ensure amplification specificity in qRT-PCR, melting curve analysis showed a single distinctive peak, revealing that no primer dimers, nonspecific amplification and targeted splice variants were generated (Fig. 4A). This hypothesis is corroborated by electrophoresis analysis of the size of PCR products showed that only one single amplicon band was generated by corresponds to GLR genes (Fig. 4B). The estimated PCR amplification efficiency of these GLRs varied from 95.1% to 105.4%, and the correlation coefficients (R^2) ranged between 0.980 and 1.000 (Table 3). The linear dynamic range covers 4 orders of magnitude and ideally extends to 5 $\log_{10}$ concentrations. Neither primer dimers nor unexpected products were found. The qRT-PCR system was demonstrated to be efficient and

specific for pear GLR gene amplification. However, *PbrGLR2.8*, *PbrGLR2.9* and *PbrGLR2.11* could not be detected by RT-qPCR in all tested samples in this study. The similar scenarios are exhibited in additional two PCR results used difference pair primers which high quality evaluation in primer-blast tool. Thus, it is possible that their expressions are too low to be detected. In order to verify this hypothesis, we used Nested PCR to improve the sensitivity of detection (Fig. 5A). We found that a weak target band of *PbrGLR2.8* and *PbrGLR2.9* were detected in the stem tissue after second round of PCR, whereas *PbrGLR2.11* was still imperceptibly (Fig. 5B). Therefore, we concluded that *PbrGLR2.8* and *PbrGLR2.9* is expressed indeed, but the expression level is too low to detect by normal RT-PCR, whereas *PbrGLR2.11* should be pseudogene in pear or the expression of which is too low to be detected, or expressed in specific tissues, or expressed under specific conditions.

7. Data analysis

In order to identify reference genes in this study, we selected ten reference genes (*PbrCYP*, *PbrUBI*, *PbrTUB*, *PbrAPT*, *PbrGAPDH*, *PbrEF1α*, *PbrPP2A*, *Pbr18S rRNA*, *PbrUBQ* and *PbrACT*), which has identified to be high quality reference genes for gene expression analysis in pear as described previously[1], as candidate reference genes. All candidate reference genes showed a moderately range of *C*t values within 22-28 in the 18 tested sample pools, except for *Pbr18SrRNA* (Fig. 6A). Three RT-qPCR analysis programs, geNorm[2] (Fig. 6B, C), NormFinder[3] (Fig. 6D) and BestKeeper[4] (Fig. 6E), were applied for systematic determinations. The results showed that two reference genes are enough to normalize GLRs expression corresponds to analysis groups. *PbrTub-2* and *PbrGAPDH* were the most stable reference genes across tissue expression pattern analysis; *PbrTub-2* and *PbrEF1α* were the most stable reference genes during stem vascular tissues development; *PbrACT* and *PbrCYP* were the most stable reference genes in shoot tip sprouted analysis (Fig. 6F). The $\Delta\Delta$Cq method was used for determining differences in pear GLR mRNA levels between samples. All NTCs in every plate have not amplification products ensured that the system is not contaminated.

Table 1. Description of 18 samples for qRT-PCR in *Pyrus bretschneideri* Rehd.

Sample no.	Sample type	Growth condition
**S1**	Root	Roots, leaves, and stems were harvested after seedling with 50 days, grown in greenhouse, 25°C; fruits and seeds were harvested 12 weeks after flowering; pistils were collected a few days before anthesis; pollen was incubated for 3 hours in liquid culture medium, 25 ^{o}C
**S2**	Stem	
**S3**	Leaf	
**S4**	Seed	
**S5**	Fruit	
**S6**	Pistil	
**S7**	Pollen	
**S8**	Phloem-stage I	Phloem, cambium and xylem were harvested from the stem which above cotyledons of 90 days-old pear tree
**S9**	Cambium-stage I	
**S10**	Xylem-stage I	
**S11**	Phloem-stage II	Phloem, cambium and xylem were harvested from current growth branches of 8 years-old pear tree in sprouted phases (April)
**S12**	Cambium-stage II	
**S13**	Xylem-stage II	
**S14**	Phloem-stage III	Phloem, cambium and xylem were harvested from last year growth branches of 8 years-old pear tree in sprouted phases (April)
**S15**	Cambium-stage III	
**S16**	Xylem-stage III	
**S17**	Sprouting shoot tip	Shoot tips were collected from 8-year-old pear tree which at sprouted phase (April)
**S18**	Dormant shoot tip	Shoot tips were collected from 8-year-old pear tree which at dormant phase (February)

Note: The samples were frozen as soon as were collected.

Table 2 RNA quality analysis in 18 samples.

#1	OD 260/280	OD 260/230	Conc. (ng/μl)	#2	OD 260/280	OD 260/230	Conc. (ng/μl)
S1	2.16	2.06	1737	S1	2.02	1.84	1905
S2	2.11	1.84	1663	S2	2.31	1.85	1784
S3	2.35	1.84	1769	S3	2.38	1.95	1218
S4	2.23	1.89	2301	S4	2.26	2.00	1438
S5	2.01	1.92	1302	S5	2.49	2.04	1370
S6	2.31	1.80	1915	S6	2.21	1.93	1574
S7	2.19	1.93	1576	S7	2.06	1.98	1336
S8	2.30	2.01	526	S8	2.17	1.83	525
S9	2.25	1.81	897	S9	2.26	2.09	802
S10	2.13	1.85	620	S10	2.26	2.00	731
S11	2.11	1.85	774	S11	2.24	1.93	1052
S12	2.26	1.86	959	S12	2.10	1.85	973
S13	2.12	1.80	512	S13	2.20	1.83	717
S14	2.14	1.99	1100	S14	2.17	1.86	1076
S15	2.28	1.90	1012	S15	2.13	1.92	634
S16	2.22	2.08	716	S16	2.26	1.83	899
S17	2.30	1.89	1851	S17	2.19	1.81	2103
S18	2.23	2.04	1738	S18	2.26	1.93	2061
**#3**	**OD 260/280**	**OD 260/230**	**Conc. (ng/μl)**	**#4**	**OD 260/280**	**OD 260/230**	**Conc. (ng/μl)**
S1	2.34	1.99	2104	S1	2.47	1.89	2461
S2	2.14	1.91	2282	S2	2.09	2.08	1493
S3	2.17	2.10	1091	S3	2.34	2.09	1599
S4	2.18	2.08	2047	S4	2.50	1.92	1237
S5	2.20	2.00	1754	S5	2.24	2.08	1526
S6	2.35	1.96	1178	S6	2.36	2.04	2396
S7	2.09	2.10	2332	S7	2.10	2.10	1949
S8	2.10	1.90	834	S8	2.26	2.03	1096
S9	2.11	1.96	672	S9	2.22	2.03	503
S10	2.28	1.93	969	S10	2.30	1.94	668
S11	2.29	1.91	833	S11	2.23	2.06	590
S12	2.22	1.84	577	S12	2.12	2.09	998
S13	2.23	1.89	742	S13	2.26	1.99	790
S14	2.18	1.90	985	S14	2.24	1.99	512
S15	2.11	1.97	780	S15	2.29	1.91	758
S16	2.18	1.83	1031	S16	2.28	1.86	964
S17	2.16	1.91	1723	S17	2.26	2.05	1212
S18	2.18	1.92	1103	S18	2.25	1.95	1588

Note: "#" representative independently biological reduplicate.

Table 3 Description of GLR gene primers for qRT-PCR in pear

Gene name	Primer sequence (5'-3')	Tm (℃)	Amplicon size (bp)	PCR efficiency (%)	Correlation coefficient (r^2)
PbrGLR1.1&1.2-F	CGTCGAGATCACACAGGATGAA	60.1	206	99.7	0.992
PbrGLR1.1&1.2-R	TCTGTTCGTTTGTCGAGGAGG	60.0			
PbrGLR1.3-F	AGAAGCTAGCCATCCTCTCGAT	60.4	99	99.4	0.996
PbrGLR1.3-R	TGTCAAGAATCAACCCGACATGA	60.2			
PbrGLR2.1-F	TTTGTCATTGGACAGAAGTCGCA	61.0	125	99.0	0.998
PbrGLR2.1-R	AAGTCCGAGAGCGCCATTTC	60.7			
PbrGLR2.2-F	ACAGAACACAACAATCCCGGT	60.1	127	96.2	0.994
PbrGLR2.2-R	AGAGGAAGCACGGGAGGTAT	60.0			
PbrGLR2.3-F	CTCAGGCCTTTCAGACCACC	60.3	113	97.8	0.997
PbrGLR2.3-R	GCTGTTGCGGAACTCTGTCT	60.6			
PbrGLR2.4-F	AGCGGCAGTCTTGGCCTTG	61.7	278	96.6	0.985
PbrGLR2.4-R	TCCAGCCAGTTGCTATCAGG	60.0			
PbrGLR2.5-F	CAATCCGACCATGAATGTGATT	58.2	263	98.3	0.995
PbrGLR2.5-R	GAAGTCGCCCATCGACAAGAC	60.9			
PbrGLR2.6-F	AGCACTAACACAACGGAAGTCA	60.1	138	98.3	0.994
PbrGLR2.6-R	ATCATCGTAAGTGCCAGCCA	59.5			
PbrGLR2.7-F	ATGGAGGTAGGGTATGCTTG	56.4	213	95.2	0.982
PbrGLR2.7-R	TCCGAACACATCCAGTTTAAC	56.4			
PbrGLR2.8-F	CAAACTCAGGCATGGTATGCT	59.1	230	/	/
PbrGLR2.8-R	TCATACGCCCACAATCCGAACA	60.2			
PbrGLR2.9-F	TCGGCATGATGAAGGCAGAT	61.8	208	/	/
PbrGLR2.9-R	TCTTTGACAAGCTGAGGACCAT	60.3			
PbrGLR2.10-F	AATGCCAGTAGTCTTGGACCT	57.3	116	97.5	0.990
PbrGLR2.10-R	CCACGCTTCACTGGAACTAG	57.0			
PbrGLR2.11-F	AGACCGCACTTACCATGCT	58.7	115	/	/
PbrGLR2.11-R	AGAGCTTGACGACGGCCTTG	60.7			
PbrGLR2.12-F	TATGTTGGCTTCAAAGCAGGT	59.4	279	95.1	0.986
PbrGLR2.12-R	AAAATCGTGCGTGAGAAGCG	57.9			
PbrGLR2.13-F	GCTAGGTTTGTAGTGGTCATCTGG	57.5	113	99.1	0.980
PbrGLR2.13-R	TCAGTAACGACTGGGTTGAGC	56.5			
PbrGLR2.14&2.15-F	ACCAGCTGCTGCTTGTTTATT	58.0	156	96.8	0.980
PbrGLR2.14&2.15-R	CAGCACTAGCTCCCGATGTA	57.9			
PbrGLR3.1&3.2-F	CATTGGAGCGTTAAAGTACATGG	58.6	129	96.5	0.998
PbrGLR3.1&3.2-R	AGCGCCGTGACTGACAATAAC	58.7			
PbrGLR3.3-F	TTCAAGCTCTGCAATTCATG	55.3	126	96.3	0.986
PbrGLR3.3-R	TGGCTGCAAATGACAATAAG	56.5			
PbrGLR3.4-F	ATGGAGGTATTGCTGATGAATAGG	58.7	215	95.5	0.983
PbrGLR3.4-R	GCAGCTAAAATTGCCGGCTT	60.7			
PbrGLR3.5-F	CCCAGGTTGCGCAAAATAACT	60.0	194	96.3	0.989
PbrGLR3.5-R	ACCTGTGACCAACCACATCT	58.1			
PbrGLR3.6&3.8-F	TGCCGTACGAGTTTGTACCT	61.3	263	96.0	0.986
PbrGLR3.6&3.8-R	GAAAATGCCTGTGACACCCC	59.3			
PbrGLR3.7-F	TGAAGATGGTTGCAGAAAACGTG	61.1	289	98.5	0.996
PbrGLR3.7-R	GTAGCGAGCTGCCTCTTAGG	59.5			
PbrGLR3.9-F	AGTATAGTTTGGCTCCAGGTGT	59.9	245	98.0	0.987
PbrGLR3.9-R	CCACTGTAATTTGAGTCCTGCT	57.8			
PbrGLR3.10-F	CACTGTAGCGGCAACGGATT	61.0	135	96.7	0.988
PbrGLR3.10-R	ACCATTTGAGGCAAACCCACT	60.4			
PbrGLR4.1&4.2-F	ATGTCTCAGTTTGCTCTTCTTTTT	57.8	254	105.4	0.986
PbrGLR4.1&4.2-R	GAGGGCTTCCTCTGAGAGT	59.7			
PbrGLR4.3-F	AATTCGTCCTAGAGGTTCAATCA	58.3	170	96.7	0.981
PbrGLR4.3-R	AGGGCTCCCTCTGAGAGTTT	59.6			
PbrGLR4.4-F	GAGCAACAGCTGCAGCAATAA	58.8	260	95.2	0.985
PbrGLR4.4-R	TCGCCATCGGAAATGACCAA	57.4			
PbrGLR4.5-F	TCTGCTGCTGAAGAATTGATAAAG	56.6	167	102.8	1.000
PbrGLR4.5-R	CATCGGCGTTGCATTAGTGG	58.5			
PbrGLR4.6-F	TGCTGCTTATGCCGCTAAAGAATTA	61.6	202	98.8	0.982
PbrGLR4.6-R	AGAACCGTCTGCTGCCATTC	58.4			

Note: "/" representative no expression was detected in this study.

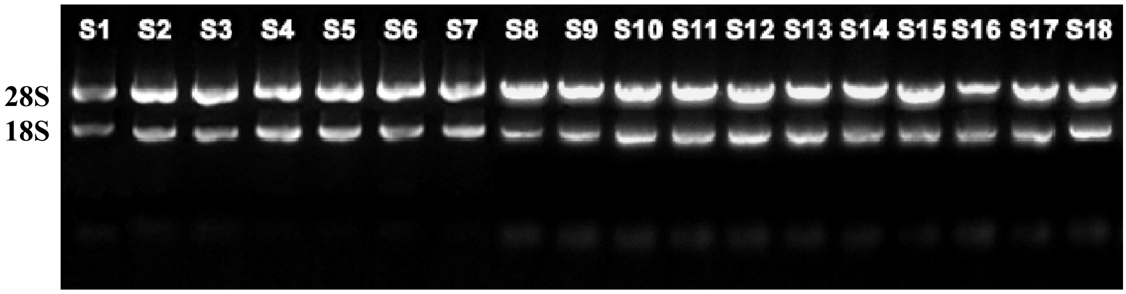

Fig. 1 Representative images of integrity RNA were extracted from 18 samples. Lane number corresponding order in Table 1.

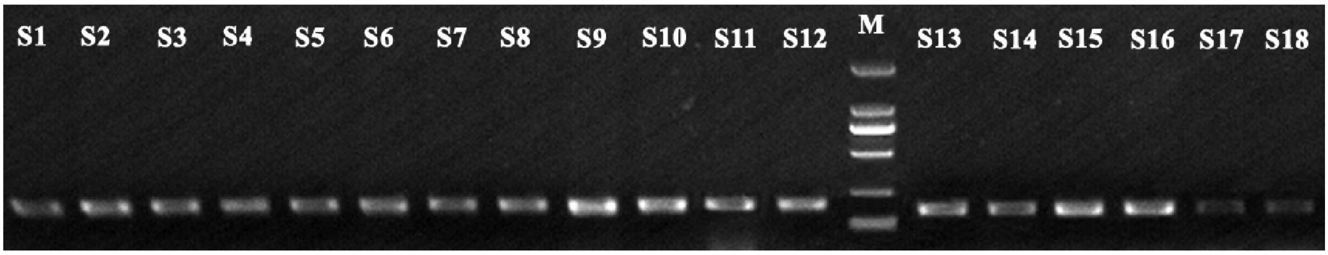

Fig. 2 Amplification results of *PbEF1α* with all samples cDNA to assay RNA extraction quality. Lane number corresponding order in Table 1. M: DL 2000 marker (in ascending order: 100, 250, 500, 750, 1000 and 2000 bp).

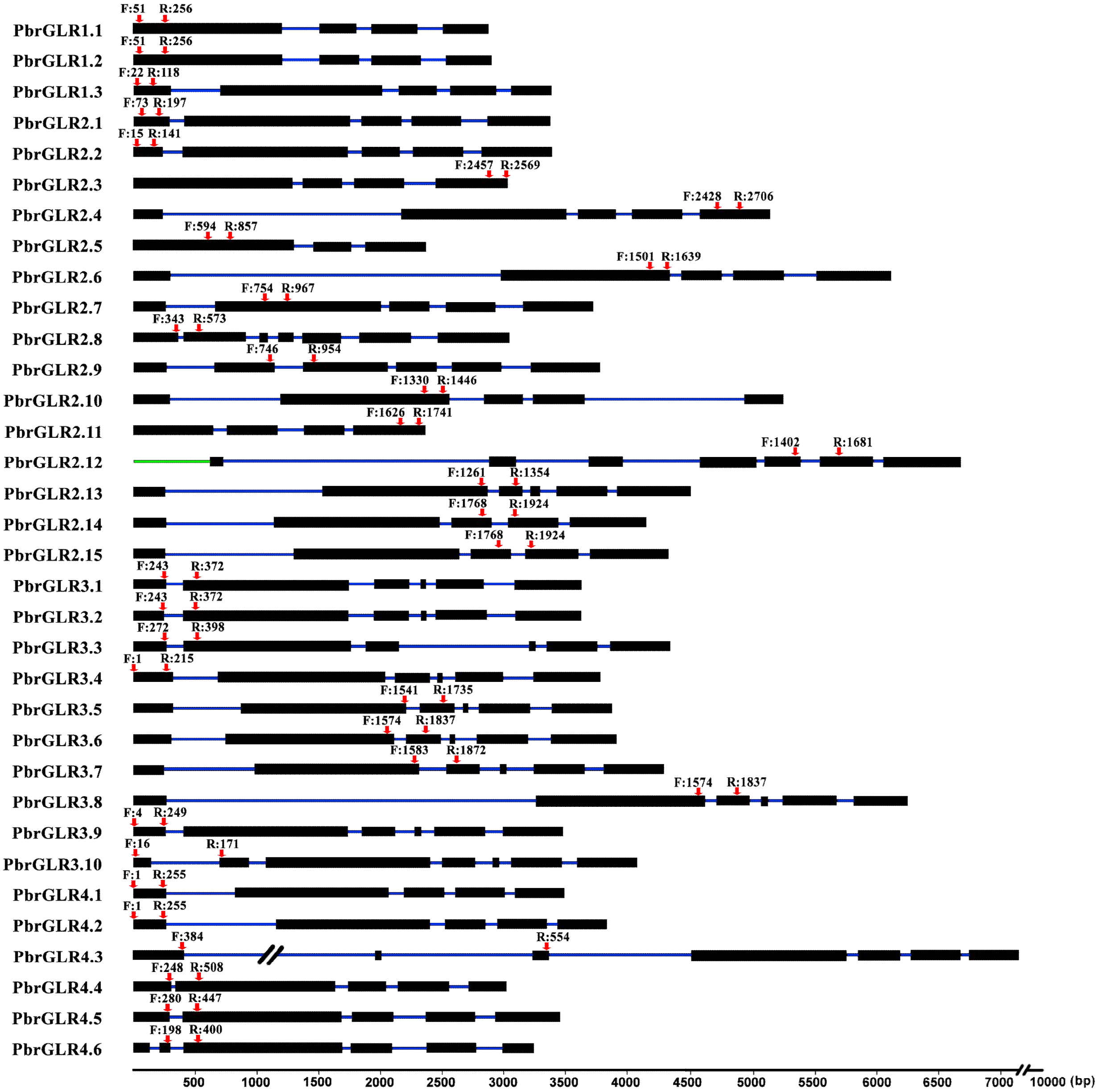

Fig. 3 Graphic representation gene structure and primer location of pear GLR genes in genomic. The black boxes, blue and green lines in the gene structure diagram represent exons, introns and UTR, respectively. The arrows refer to the primer on the gene location, i.e. F: Forward primer, R: Reverse primer, and follow their number on behalf of the CDS location of primer.

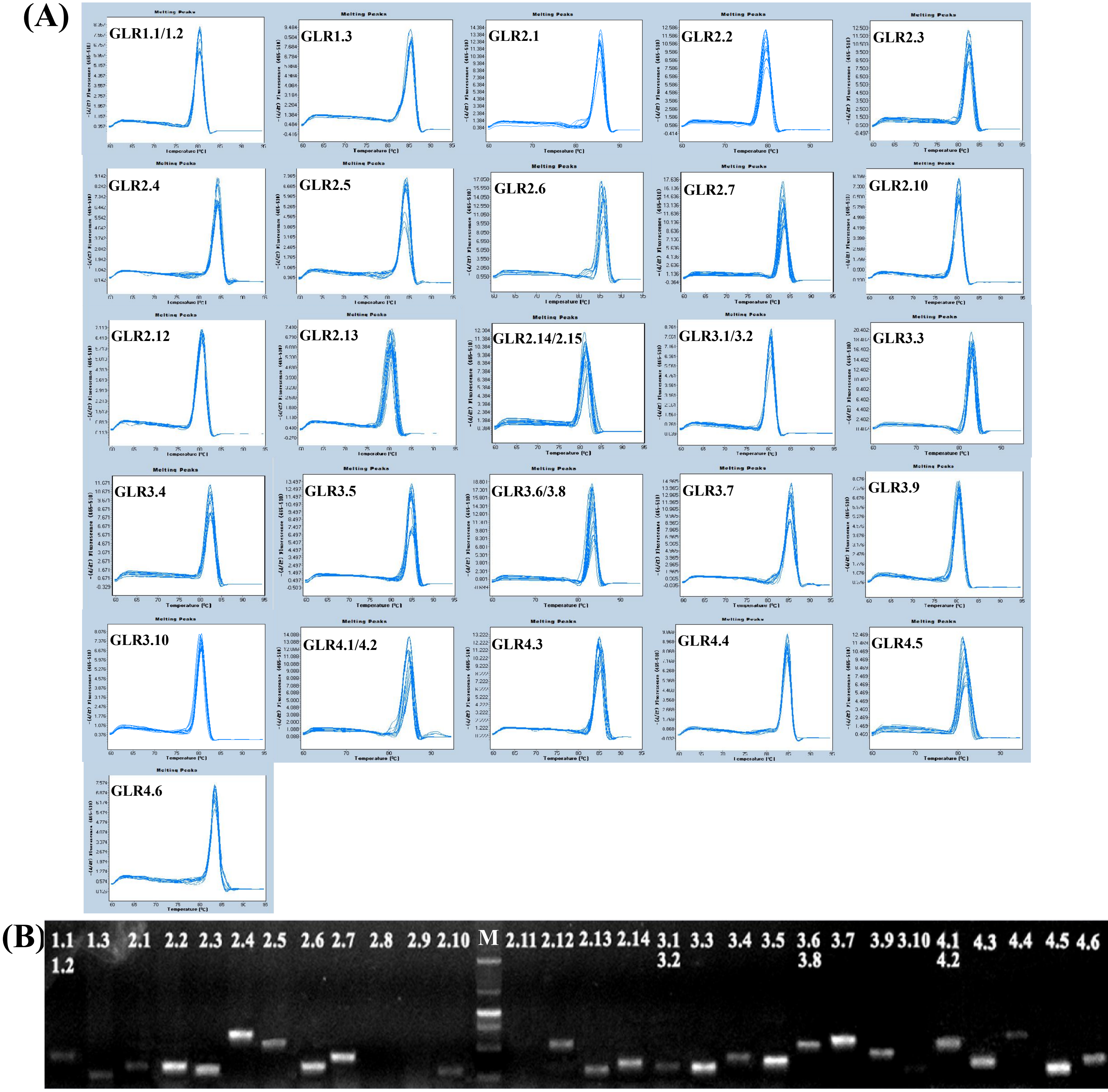

Fig. 4 Confirmation of primer specificity and amplicon size in qRT-PCR. (A) Melting curves of *PbrGLR* genes showing single peaks. (B) Amplification results of *PbrGLR* genes correspond to the predicted size. M: DL 2000 marker (in ascending order: 100, 250, 500, 750, 1000 and 2000 bp). Agarose gel (2.0%) showing specific RT-qPCR products of the expected size for each gene.

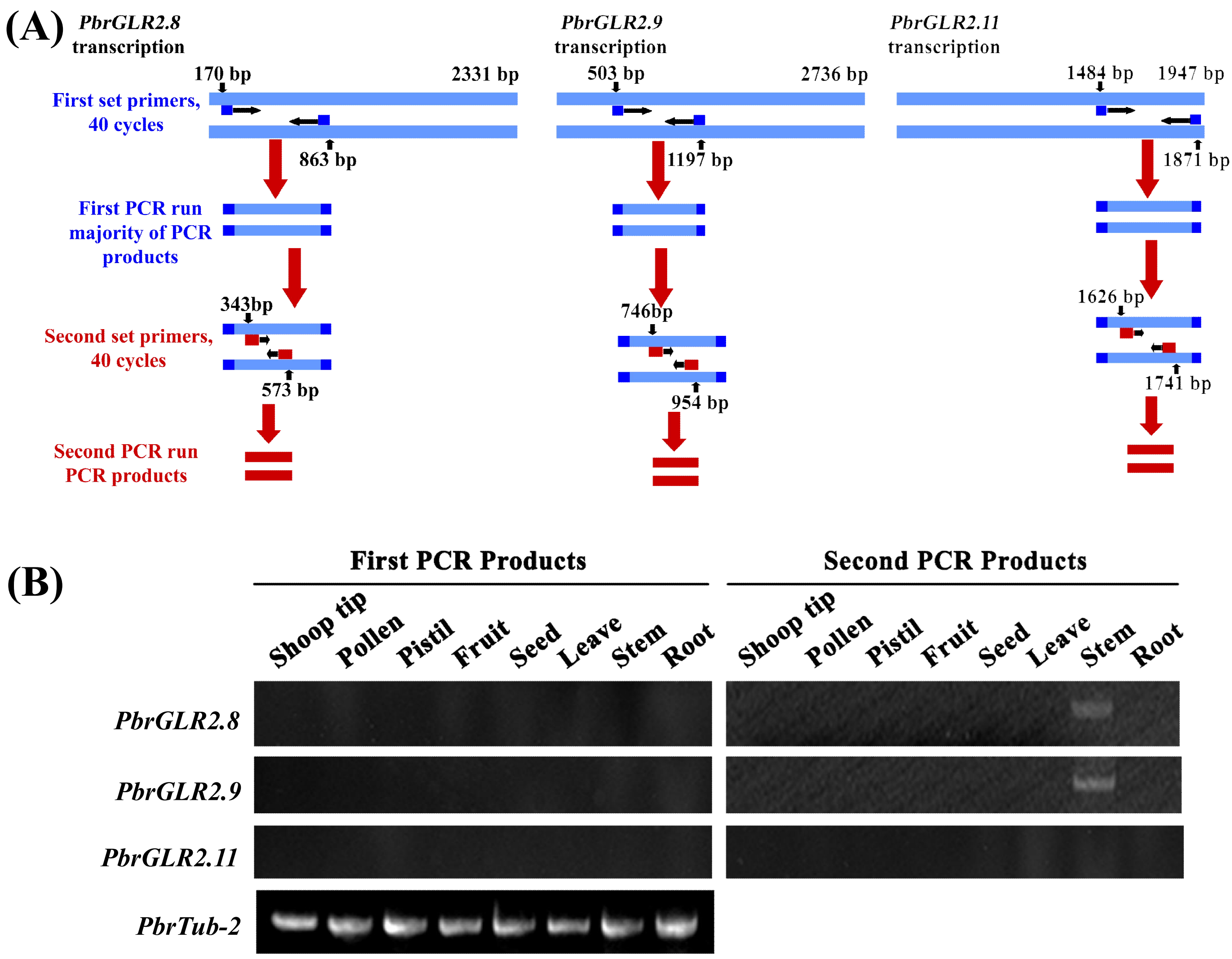

Fig. 5 Nested PCR to improve the sensitivity of detection in *PbrGLR2.8, PbrGLR2.9* and *PbrGLR2.11* mRNA levels.

(A) Schematic diagram of Nested PCR in the detection of *PbrGLR2.8, PbrGLR2.9* and *PbrGLR2.11*. **(B)** Electrophoresis of Nested PCR products. An amplified *PbrTub-2* gene was used as an internal control in Nested PCR.

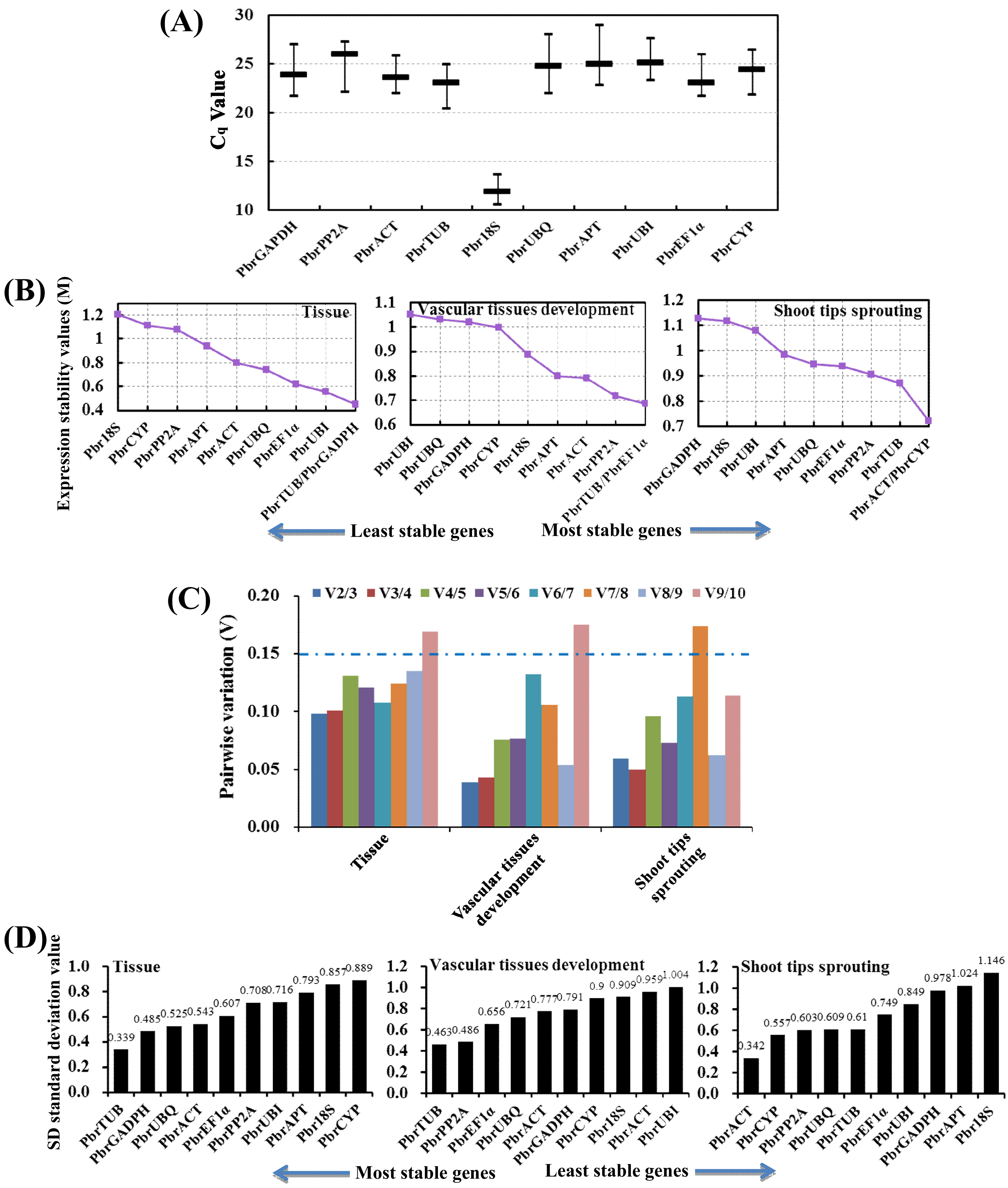

(A)
Cq Value
PbrGAPDH
PbrPP2A
PbrACT
PbrTUB
Pbr18S
PbrUBQ
PbrAPT
PbrUBI
PbrEF1α
PbrCYP
(B)
Expression stability values (M)
Tissue
Vascular tissues development
Shoot tips sprouting
Pbr18S
PbrCYP
PbrPP2A
PbrAPT
PbrACT
PbrUBQ
PbrEF1α
PbrUBI
PbrTUB/PbrGADPH
PbrGADPH
PbrTUB/PbrEF1α
PbrTUB
PbrACT/PbrCYP
Least stable genes
Most stable genes
(C)
V2/3
V3/4
V4/5
V5/6
V6/7
V7/8
V8/9
V9/10
Pairwise variation (V)
Tissue
Vascular tissues development
Shoot tips sprouting
(D)
SD standard deviation value
Tissue
Vascular tissues development
Shoot tips sprouting
Most stable genes
Least stable genes

(E)

Sample \ Rank	1	2	3	4	5	6	7	8	9	10
Tissue	*PbrTUB* (0.151)	*PbrGADPH* (0.232)	*PbrEF1α* (0.353)	*PbrUBQ* (0.416)	*PbrUBI* (0.447)	*PbrACT* (0.483)	*PbrCYP* (0.555)	*PbrPP2A* (0.654)	*PbrAPT* (0.661)	*Pbr18S* (0.696)
Vascular tissues development	*PbrTUB* (0.063)	*PbrEF1α* (0.171)	*PbrACT* (0.202)	*PbrPP2A* (0.243)	*PbrCYP* (0.288)	*PbrAPT* (0.342)	*Pbr18S* (0.397)	*PbrGADPH* (0.473)	*PbrUBQ* (0.557)	*PbrUBI* (0.611)
Shoot tips sprouting	*PbrACT* (0.094)	*PbrCYP* (0.165)	*PbrPP2A* (0.313)	*PbrTUB* (0.341)	*PbrEF1α* (0.381)	*PbrAPT* (0.506)	*PbrUBQ* (0.541)	*PbrUBI* (0.566)	*Pbr18S* (0.596)	*PbrGADPH* (0.658)

(F)

Sample groups	Optimal reference gene				
	geNorm		Normfinder	BestKeeper	Common gene
**Tissue**	*PbrTUB*	*PbrGADPH*	*PbrTUB*	*PbrTUB*	*PbrTUB/PbrGADPH*
**Vascular tissues developmen**	*PbrTUB*	*PbrEF1α*	*PbrTUB*	*PbrTUB*	*PbrTUB/PbrEF1α*
**Shoot tips sprouting**	*PbrACT*	*PbrCYP*	*PbrACT*	*PbrACT*	*PbrACT/PbrCYP*

Fig. 6 Systematic determinations reference gene in tissue samples, vascular tissues development samples and shoot tips samples in pear.

(A) Expression levels of 10 candidate reference genes tested in 18 samples of three sample groups in pear. The mean Cq values of 10 candidate reference genes in all pear samples. The boxes represent mean Cq values. The bars indicate the maximum and minimum values. **(B)** Expression stability analysis of 10 reference genes in the three sample groups by geNorm software. A lower average expression stability value indicates more stable expression. **(C)** Pairwise variation (V) calculated by geNorm to analyze the optimal number of 10 pear reference genes in three sample groups. The average pairwise variations (V_n/V_{n+1}) were analyzed to measure the effect of adding the reference gene on the RT-qPCR normalization for all samples. **(D)** Expression stability analysis of 10 reference genes in the three sample groups by BestKeeper. SD standard deviation. A lower average expression stability value indicates more stable expression. **(E)** Ranking of candidate reference genes in order of their expression stability as calculated by NormFinder. **(F)** Optimal and worst pear reference genes in nine sample groups by three methods.

Reference

1 Chen, J. *et al.* Identification and testing of reference genes for gene expression analysis in pollen of Pyrus bretschneideri. *Scientia Horticulturae* **190**, 43-56 (2015).

2 Vandesompele, J. *et al.* Accurate normalization of real-time quantitative RT-PCR data by geometric averaging of multiple internal control genes. *Genome biology* **3**, research0034

(2002).

3 Pfaffl, M. W., Tichopad, A., Prgomet, C. & Neuvians, T. P. Determination of stable housekeeping genes, differentially regulated target genes and sample integrity: BestKeeper–Excel-based tool using pair-wise correlations. *Biotechnology letters* **26**, 509-515 (2004).

4 Andersen, C. L., Jensen, J. L. & Ørntoft, T. F. Normalization of real-time quantitative reverse transcription-PCR data: a model-based variance estimation approach to identify genes suited for normalization, applied to bladder and colon cancer data sets. *Cancer research* **64**, 5245-5250 (2004).
